# General environmental heterogeneity as the explanation of sexuality? Comparative study shows that ancient asexual taxa are associated with both biotically and abiotically homogeneous environments

**DOI:** 10.1002/ece3.3716

**Published:** 2017-12-12

**Authors:** Jan Toman, Jaroslav Flegr

**Affiliations:** ^1^ Faculty of Science Laboratory of Evolutionary Biology Department of Philosophy and History of Sciences Charles University Prague Czech Republic

**Keywords:** ancient asexuals, asexual reproduction, Frozen evolution theory, habitat heterogeneity, sexual reproduction

## Abstract

Ecological theories of sexual reproduction assume that sexuality is advantageous in certain conditions, for example, in biotically or abiotically more heterogeneous environments. Such theories thus could be tested by comparative studies. However, the published results of these studies are rather unconvincing. Here, we present the results of a new comparative study based exclusively on the ancient asexual clades. The association with biotically or abiotically homogeneous environments in these asexual clades was compared with the same association in their sister, or closely related, sexual clades. Using the conservative definition of ancient asexuals (i.e., age >1 million years), we found eight pairs of taxa of sexual and asexual species, six differing in the heterogeneity of their inhabited environment on the basis of available data. The difference between the environmental type associated with the sexual and asexual species was then compared in an exact binomial test. The results showed that the majority of ancient asexual clades tend to be associated with biotically, abiotically, or both biotically and abiotically more homogeneous environments than their sexual controls. In the exploratory part of the study, we found that the ancient asexuals often have durable resting stages, enabling life in subjectively homogeneous environments, live in the absence of intense biotic interactions, and are very often sedentary, inhabiting benthos, and soil. The consequences of these findings for the ecological theories of sexual reproduction are discussed.

## 1 INTRODUCTION

### 1.1 Paradox of sexual reproduction

Sexual reproduction (sensu amphimixis, the alternation of meiosis and syngamy) is one of the most enigmatic phenomena in evolutionary biology (see, e.g., Bell, 1982; Maynard Smith, 1978; Meirmans & Strand, 2010; Williams, 1975), mainly because it brings many obvious disadvantages in comparison with asexual reproduction—the well‐known twofold cost of sex being only the first and most obvious one (see, e.g., Lehtonen, Jennions, & Kokko, 2012). None of these disadvantages apply to all sexual species because of the highly variable nature of their reproduction. However, under many circumstances, the disadvantages apply profoundly (Lehtonen et al., 2012). Thus, sexual reproduction, its overwhelming predominance, and its long‐term maintenance in eukaryotes remain an enigma that call for explanation.

Many main concepts and their countless variants were proposed to explain the paradox of sexual reproduction (reviewed, e.g., in Bell, 1982, 1985; Kondrashov, 1993; Maynard Smith, 1978; Meirmans & Strand, 2010; Otto, 2009; Sharp & Otto, 2016; Williams, 1975). The genetic advantages of sex for sexually reproducing populations or individuals are highlighted by concepts such as the Weismann's idea of sex generating variability, later delimited as the hypothesis of Vicar of Bray (Bell, 1982), Fisher–Muller's accelerated evolution of sexual species (Fisher, 2003; Muller, 1932), breaking free of neighboring deleterious mutations (Crow, 1970), reduction of the spread of genomic parasites (Sterrer, 2002), advantage of diploidy (Lewis & Wolpert, 1979), repair of DNA (Bernstein & Bernstein, 2013), restoration of epigenetic signals (Gorelick & Carpinone, 2009), eventually stochastic and deterministic variants of Muller's ratchet hypothesis (Kondrashov, 1982; Muller, 1964). These concepts are not mutually exclusive and underwent their own evolution during the last decades, leading to some convincing scenarios of the spread of sexuality and its long‐term predominance (see, e.g., Keightley & Otto, 2006; Otto, 2009; Otto & Lenormand, 2002; Sharp & Otto, 2016).

Ecological theories of sexual reproduction, on the other hand, stress the assumption that sex provides some ecological advantage to sexual species. Certain trends can be clearly found in the geographic distribution of sexual reproduction, as was recently summarized by Hörandl (2006, 2009) or Vrijenhoek and Parker (2009). Moreover, primarily asexual prokaryotes are abundant and, as will be shown later in this study, clear examples of short‐term and long‐term secondarily asexual eukaryotic taxa have been identified. Sex is obviously not universally advantageous. It was also suggested that some advantages of sexual reproduction postulated by “genetic theories” could be achieved by automixis (Gorelick & Carpinone, 2009; Neiman & Schwander, 2011; but see also Keightley & Otto, 2006; Otto, 2009; Otto & Lenormand, 2002; Sharp & Otto, 2016).

However, ecological theories of sexual reproduction need not contradict the benefits of sex identified by “genetic theories.” In fact, “genetic theories” that consider adaptiveness are necessarily related to ecological phenomena, and most “ecological theories” have important genetic components as well (see Otto, 2009; Otto & Lenormand, 2002; Sharp & Otto, 2016). The difference lies mainly in their target of interest. “Ecological theories” focus on the direct, ecological, conditions that facilitate the evolution, spread, and long‐term predominance of sex. Therefore, it might be more correct to designate them as ecology‐dependent (in contrast to ecology‐independent theories mentioned above). In any case, the final answer to the “greatest paradox of evolutionary biology” probably lies in the group of ecological theories of sexual reproduction, respectively, in some form of theoretical synthesis that incorporates the assumptions of both genetic and ecological theories of sex (Otto, 2009; Otto & Lenormand, 2002; Scheu & Drossel, 2007; Sharp & Otto, 2016; Song, Drossel, & Scheu, 2011; West, Lively, & Read, 1999).

### 1.2 Ecological theories of sexual reproduction and their predictions

“Ecological theories” such as the Red Queen theory (Hamilton, Axelrod, & Tanese, 1990), the evolutionary arm‐races hypothesis (Dawkins & Krebs, 1979), and the fast‐sexual‐response hypothesis of Maynard Smith (1993) emphasize the sexually reproducing organisms’ advantage when interacting with other organisms that are able to dynamically react in a coevolutionary manner. According to these “biotic heterogeneity advantage” theories (see Table 1), sexual species should prosper in spatially and temporally biotically heterogeneous environments, that is, environments with many biotic interactions from competitors, predators, and parasites (see Table 2). In the presence of such intensive biotic interactions, sexual species are expected to be especially favored because they maintain high‐genetic polymorphism and could quickly react to the counter‐adaptations of their evolutionary opponents by a simple change of allele frequencies in the population. The speed, not the depth, of adaptation is more important in these environments (Maynard Smith, 1993).

**Table 1:** Ecological theories of sexual reproduction

“Biotic heterogeneity advantage” theories	E.g. Red Queen theory (Hamilton, et al. 1990), evolutionary arm‐races hypothesis (Dawkins & Krebs, 1979), fast‐sexual‐response hypothesis (Maynard Smith, 1993)
“Abiotic heterogeneity advantage” theories	E.g. Lottery and Sisyphean genotypes hypothesis (Williams, 1975), elbow room hypothesis (Maynard Smith, 1978), tangled bank hypothesis (Bell, 1982), hypothesis of fluctuating selection (Smith, 1980), hypothesis of reduced response to fluctuating selection (Roughgarden, 1991)
“Overall heterogeneity advantage” theories	E.g. hypothesis of genetic polymorphism in fluctuating environments (Williams, 1975), frozen plasticity theory (Flegr, 2013), concept of density‐dependent–independent population regulation (Scheu & Drossel, 2007; Song, et al. 2011)

A classification of ecological theories of the maintenance of sexual reproduction presented in this paper. Given the extraordinary plethora of proposed concepts, this summary cannot be exhaustive nor complete. Only the major concepts as they were originally proposed are included.

**Table 2:** Biotically and abiotically heterogeneous environments

	*Biotically heterogeneous environments*	*Abiotically heterogeneous environments*
Characteristics	Environments with numerous and/or intensive biotic interactions among competitors and hosts and their predators/parasites that are characteristic by dynamic coevolutionary reactions	Spatiotemporally abiotically very variable environments, i.e. patchy, diverse, changeable, unpredictable, and with unequally distributed resources
Examples	Tropical rainforests, low‐latitude coral reefs, ancient lakes, habitats with climax communities or generally with species‐rich complex ecosystems	Temporary, ephemeral or exposed habitats, dynamically changing freshwater environments, coastal habitats, biomes of high latitudes and/or altitudes

Main characteristics of biotically and abiotically heterogeneous environments in the optics of ecological theories of sexual reproduction and examples of habitats that are characteristic by strong biotic and abiotic heterogeneity.

Another group of ecological theories of sexual reproduction comprises, for example, the lottery and Sisyphean genotypes hypothesis (Williams, 1975), elbow room hypothesis (Maynard Smith, 1978), tangled bank hypothesis (Bell, 1982), hypothesis of fluctuating selection (Smith, 1980), and hypothesis of reduced response to fluctuating selection (Roughgarden, 1991). These “abiotic heterogeneity advantage” theories (see Table 1) see the main advantage of sexual reproduction in the higher fitness that sexual individuals or species achieve in abiotically heterogeneous environments—environments that are abiotically variable in space and/or time, that is, diverse, unpredictable, and with unequally distributed resources (see Table 2). An abiotic environment does not co‐evolutionarily react to the evolutionary moves of its inhabitants, potentially allowing them to deeply adapt to it under certain circumstances, for example, under conditions of slow, long‐term changes. Under these circumstances, the asexual species might have an advantage because, for example, they do not suffer from segregation and recombination loads (Crow, 1970). However, the spatial and temporal heterogeneity of an environment is expected to usually ensure the advantage of sexual species.

The heterogeneity of the environment, both biotic and abiotic, can be comprehended as the sum of heterogeneity in space (in the sense of variability, e.g., patchiness) and time (in the sense of instability, especially when the change is unpredictable). Both spatial and temporal heterogeneity could be the consequences of both biotic and abiotic factors (Li & Reynolds, 1995). The temporal and spatial aspects of heterogeneity, even though differing substantially at first sight, could act remarkably similarly in terms of favoring sexual species (Kondrashov, 1993; Neiman & Schwander, 2011; Otto, 2009; Otto & Lenormand, 2002; Scheu & Drossel, 2007; Sharp & Otto, 2016; Song et al., 2011). In principle, the most important factor is always whether the environment inhabited by the offspring differs in its character (i.e., selective pressures) from the environment inhabited by their parents.

**Figure 1:**
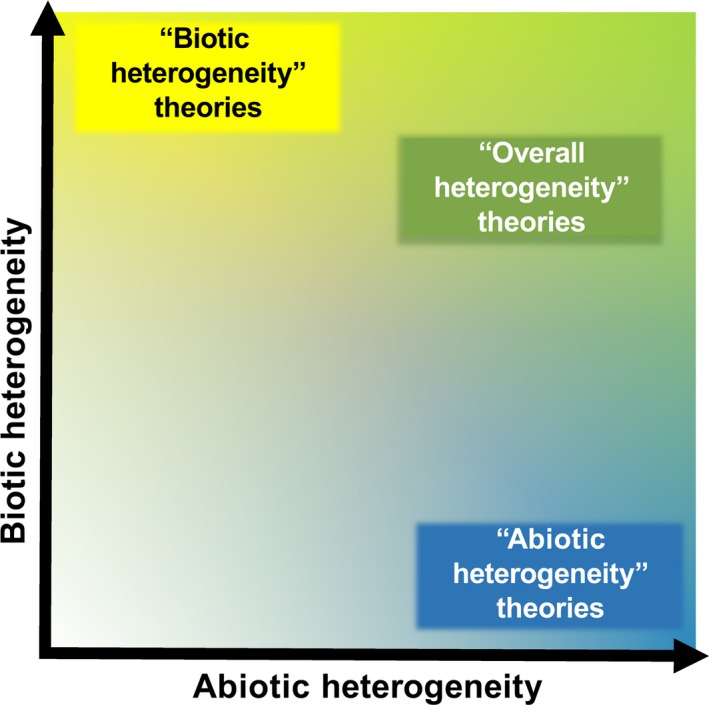
Ecological theories of sexual reproduction and their predictions regarding environmental heterogeneity. Diagram illustrating predictions of ecological theories of sexual reproduction regarding environmental heterogeneity. “Biotic” theories consider highly biotically heterogeneous environments (y axis, yellow) to be those that promote sexuality over asexual reproduction. “Abiotic” theories, on the other hand, highlight abiotically heterogeneous environments (x axis, blue) in this regard. Excluding more complicated models, abiotic heterogeneity has no role in “biotic” theories and *vice versa*. This is in stark contrast with several concepts that consider both kinds of environmental heterogeneity important for promoting sexual reproduction (green). Color saturation indicates hypothetical advantage of sexual organisms over asexuals in given conditions according to each group of theories

The “biotic” and “abiotic” theories of sexual reproduction mentioned above have different predictions regarding the character of the environment that will be advantageous for sexual and asexual species. According to the major source of the environmental heterogeneity, it is therefore essentially possible to differentiate between these two groups of ecological theories of sexual reproduction. However, the predictions of different theories are not absolutely disparate—one could easily devise examples of environments suitable for asexual species according to both groups of theories, for example, stable extreme environments. Similarly, the individual theories of sexual reproduction are far from being disparate; they are usually interconnected in their basic principles, they intermingle and complement each other (Meirmans & Strand, 2010; Otto, 2009; Otto & Lenormand, 2002; Scheu & Drossel, 2007; Sharp & Otto, 2016; Song et al., 2011). Moreover, biotic and abiotic parts of the environmental heterogeneity, as well as other factors, are usually interconnected and influence and complement each other in their effects on the advantage of sexual or asexual reproduction (Glesener & Tilman, 1978; see also Otto & Lenormand, 2002; Otto, 2009; Sharp & Otto, 2016). It is therefore possible that the differentiation of “biotic” and “abiotic” ecological theories of sex is important in theory, but not important in the real world, and that sexual organisms do have an advantage in environments that are both biotically and abiotically relatively heterogeneous (i.e., overall heterogeneous environments, see Table 1 and Figure 1).

The fitness values of alleles of sexual species are often frequency‐ and contextually dependent (on other alleles of the same gene, alleles of other genes, or particular traits). Such alleles (as well as alleles that are pleiotropically or epistatically interconnected with them) are not easily fixated or eliminated. Therefore, sexual species usually maintain high‐genetic polymorphism that enables them to readily react to momentary changes of environment (by the changes in the frequency of already present alleles). However, the same factor (frequency‐ and contextually dependent fitness values of alleles) is expected to slow‐down or, eventually, stop this response as soon as the frequency of present alleles significantly change. Therefore, it is possible that sexual species, in contrast to asexual ones, are usually not able to fully adapt to transient environmental changes; they mostly retain some genetic polymorphism that helps them escape extinction when the conditions quickly return to normal. It was suggested by Williams (1975 pp. 145–146, 149–154, 169) and explicitly discussed by Flegr (2008, 2010, 2013) that the resulting lower ability of sexual species to fully adapt to transient environmental changes may bring them, paradoxically, a major advantage in randomly fluctuating environments, that is, in environments expressing large (biotic or abiotic) heterogeneity in time.

According to this (meta‐)hypothesis (see Table 1 and Figure 1), asexuals would prevail in stable or predictively slowly changing, possibly extreme, environments of low‐temporal heterogeneity (Flegr, 2013). Given the similarities between the effect of temporal and spatial heterogeneity mentioned above, this notion can be readily extended to encompass both temporal and spatial heterogeneity. Similar remarks were made, for example, by Williams (1975, p. 153) and Roughgarden (1991), while a combination of several aspects of heterogeneity was implicitly also proposed as the explanation of the presence of sexual reproduction by Glesener and Tilman (1978) and some interpreters of the Red Queen theory (e.g., Butlin, Schön, & Martens, 1999) or the tangled bank hypothesis (e.g., Bell, 1982; Scheu & Drossel, 2007; Song et al., 2011).

Otto (2009) and Sharp and Otto (2016) identified a plethora of factors that enable the spread and long‐term predominance of sex in computer simulations, spatiotemporal heterogeneity, and varying selection pressures being among the most important. Moreover, the assumption of Flegr (2008, 2010, 2013) that the contextually dependent fitness value of alleles is a major factor in maintaining high long‐term genetic variability of sexual populations seems to be empirically supported (see Otto, 2009). This hypothesis was also supported by the results of certain experimental studies, for example, long‐term patterns of fitness and genetic variability (Renaut, Replansky, Heppleston, & Bell, 2006) or dynamics of adaptation (Colegrave, Kaltz, & Bell, 2002; Kaltz & Bell, 2002) in sexually and asexually reproducing *Chlamydomonas*. Furthermore, it is in accordance with theoretical modeling (Scheu & Drossel, 2007; Song et al., 2011) and empirical testing (e.g., Bluhm, Scheu, & Maraun, 2016) of concepts that consider density‐dependent and independent population regulating factors as the main factors favoring sexual or asexual reproduction.

### 1.3 Comparing the ecology of sexual and asexual groups

Most of the organisms that live on Earth, Archaea and Bacteria, are primarily asexual. The primary asexuality is a plesiomorphic trait and therefore does not need any special explanation. In contrast, most of the known species, eukaryotes, are primarily sexual (Speijer, Lukes, & Elias, 2015) while only some eukaryotic lineages switched to secondary asexual reproduction (de Meeus, Prugnolle, & Agnew, 2007; Speijer et al., 2015; Van Dijk, 2009). It is therefore possible to compare the environmental biotic heterogeneity and abiotic heterogeneity of such secondary asexual clades with that of their sexual relatives to test particular ecological hypotheses of sexual reproduction.

Most studies aimed at testing and discriminating between individual ecological theories of sexual reproduction on the basis of their predictions about the environmental correlates of sexual and asexual lineages showed largely inconclusive results. Often their aim was to test particular theoretical concepts: lottery hypothesis and Sisyphean genotypes hypothesis (Hörandl, 2009; Williams, 1975), elbow room hypothesis (Garcia & Toro, 1992; Koella, 1993), Red Queen theory (Burt & Bell, 1987; Neiman & Koskella, 2009), fast‐sexual‐response hypothesis (Becerra, Brichette, & Garcia, 1999), hypothesis of optimal responsibility to fluctuating selection (Griffiths & Butlin, 1995; Schön & Martens, 2004), hypothesis of prevention of loss of genetic variability under fluctuating selection (Hörandl, 2009; Maynard Smith, 1993; Vrijenhoek & Parker, 2009), or tangled bank hypothesis (Burt & Bell, 1987; Domes, Scheu, & Maraun, 2007; Griffiths & Butlin, 1995; Maraun, Norton, Ehnes, Scheu, & Erdmann, 2012; Vrijenhoek, 1984); or at least they were later interpreted as such. The most extensive comparison not focused on testing one particular theoretical concept was performed by Bell (1982) on multicellular animals (Metazoa). It mostly supported the tangled bank hypothesis. Experiments aimed at discriminating the selective pressures of biotically (see, e.g., Fischer & Schmid‐Hempel, 2005) or abiotically (see, e.g., Becks & Agrawal, 2010) heterogeneous and homogeneous environments were also performed, mostly pointing to the conclusion that heterogeneous environments select higher rates of recombination or sexual reproduction. However, particular mechanisms that favor higher levels of sex are hard to determine in these cases that are, moreover, often based on facultatively sexual organisms.

The main problem of the comparative studies mentioned above may be the inclusion of both old and young asexual taxa. Most secondary asexual groups probably are not evolutionarily viable in the long term, as could be deduced from the distribution of asexual lineages on the “tree of life.” With the exception of several ancient asexuals (AAs), they form only the terminal twigs—species and genera (Butlin, 2002). This pattern is probably the consequence of the opportunistic nature of their transition to asexual reproduction and subsequent failure in species selection (Nunney, 1989), or the higher persistence of sexual lineages in the process of stability‐based sorting (Toman & Flegr, 2017). Moreover, at least some young asexual lineages could, in fact, consist of short‐lived clones continuously cleaved from maternal sexual population (Janko, Drozd, Flegr, & Pannell, 2008; Vrijenhoek & Parker, 2009). Alternatively, they could be sustained by an occasional hybridization with related sexual lineages (Butlin, Schön, & Martens, 1998; van Raay & Crease, 1995; Turgeon & Hebert, 1994) or an infrequent transfer of genetic material from “host species” in hybridogenetic and gynogenetic lineages (Bogart, Bi, Fu, Noble, & Niedzwiecki, 2007; Mantovani, Passamonti, & Scali, 2001). In sum, young asexuals do not have to exhibit the properties that would allow them to survive in the long term, the reasons of their temporary success might, in contrast to the AA lineages, differ from case to case, and, contrary to the mainstream view, they could in fact bring a significant noise into the studies of long‐term maintenance of sexual (and secondary asexual) reproduction.

### 1.4 Aims of the study

The main aim of this study was to map the environmental heterogeneity of well‐supported AA groups and identifies possible trends in its differences from the environmental heterogeneity of their closely related sexual clades. In the first part of the study, we compiled data on the environmental heterogeneity of AAs and their sexual controls. In the second, analytical, part of the study, we used the data to test whether AAs more often inhabit (1) generally less heterogeneous environments, (2) less biotically heterogeneous environments, or (3) less abiotically heterogeneous environments. To this end, we used paired exact tests to compare the ecological demands of sexual species and AA species within unrelated clades of eukaryotic organisms. In the third, exploratory, part of the study, we searched for particular environmental properties and organismal adaptations that are common among the AA members of the pairs.

As we outlined in the previous section, the phenomenon of asexual “terminal twigs contra ancient asexuals” is still somewhat controversial, and its real existence is being discussed (see, e.g., Janko, Drozd, & Eisner, 2011; Neiman, Meirmans, Meirmans, Schlichting, & Mousseau, 2009; Schön, Martens, & Rossi, 1996; Schwander & Crespi, 2009). Regardless of these discussions, it is obvious that out of all the secondary asexual clades only the AAs have been able to survive or even diversify in an asexual state for millions of years (Judson & Normark, 1996; Neiman et al., 2009; Normark, Judson, & Moran, 2003; Schurko, Neiman, & Logsdon, 2009; Schwander & Crespi, 2009). This is the main reason that our study is based exclusively on AAs as they already proven to be evolutionarily viable in the long term.

However, it is worth mentioning that the focus on AAs puts forward another serious difficulty: These clades were separated from their sister sexual lineages a long time ago (at least 1 million years ago, see Materials and Methods), and both sexual and asexual lineages thus underwent considerable time periods of independent evolution. Therefore, both lineages independently acquired numerous adaptations that distinguished them but need not be related to the mode of their reproduction. Singular case studies comparing AAs and their sexual sister lineage thus are not expected to have a strong predictive value in the long‐term maintenance of asexual reproduction. On the other hand, a comparative study enables us to compare several such pairs of AAs and sexual controls and reveal possible common adaptations of AAs related to their long‐term survival in an asexual state.

## 2 MATERIALS AND METHODS

### 2.1 Identification of ancient asexuals and their sexual controls

#### 2.1.1 Ancient asexual groups

The definition of the “ancient asexual group” is rather vague. Some researchers consider a lineage to be AA if it reproduces obligately asexually for at least 50,000 generations or 0.5 million years (Law & Crespi, 2002a); some prefer one million generations (Schwander, Henry, & Crespi, 2011), yet others just speak about “millions of years” (Judson & Normark, 1996; Normark et al., 2003). It was even suggested that AAs are not substantially different from other asexuals and their delimitation is more or less arbitrary (Neiman et al., 2009). It is not the aim of this study to argue for the substantial difference of AAs from other asexuals or against it. We focus only on groups that were proven to survive exclusively in an asexual state for a considerable amount of time. Thus, regardless of the discussion on the fundamental distinction of young and old asexual taxa, in the current study we defined AAs conservatively as those secondary asexual eukaryotic lineages that reproduce obligately asexually with a great deal of certainty for at least one million years (see Table S1 for details).

At the beginning, we identified well‐supported AA groups with the help of literary sources. We started with published secondary literature such as Judson and Normark (1996), Normark et al. (2003), Neiman et al. (2009), Schurko et al. (2009), Schwander and Crespi (2009), and Speijer et al. (2015), investigated cited primary literature and other novel primary literal sources concerning putative AA groups. We also investigated other possible AAs proposed in the primary literature and some lineages traditionally believed to be long‐term asexual. The evidence for confirmation or rejection of putative AAs included organismal, life history, palaeontological, biogeographical, molecular, individual genetic, and population genetic data and also other indices of ancient asexuality proposed in the AA literature listed above. The list of supported and contested AA candidates, as well as reasons for our decision, is summarized in Table S1. Only well‐supported AA groups were included in our comparative study.

#### 2.1.2 Sexual controls

In the next step, we identified ecologically comparable sexual sister lineages for the eight AA groups using literary sources. In those individual cases in which the phylogenetic relations between the sexual and asexual lineages were not entirely clear, we used the closest possible comparable clades (see Table S2 for details). Three of the AA groups were monophyletic (Bdelloidea, Darwinulidae, and *Vittaria*). The remaining AA groups were polyphyletic, that is, they included several related monophyletic asexual sublineages with interstitial sexual lineages. We treated each of these groups as single unit in the analysis. In these cases, we compared every individual AA lineage with its sexual control in the monophyletic subtaxa of the polyphyletic AA group and based our conclusions on the prevailing trend (i.e., over 50% of the cases; however, all actual trends were much more convincing, see Table 3) in the whole polyphyletic group. With the exception of *Timema*, the internal phylogenetic relationships of the studied polyphyletic AA groups were more or less unclear. Where possible, we proceeded using the most probable relationships (Bdelloidea, Darwinulidae, Oribatidae, Nematalycidae and Proteonematalycidae, Grandjeanicidae, and Oehserchestidae, see Table S2). In the cases with several equally probable alternative phylogenetic relationships of AA and sexual lineages (both in monophyletic/*Vittaria/*, and polyphyletic/*Alicorhagia* and *Stigmalychus*, Pomerantziidae, *Vittaria*,* Lasaea*/AA taxa), we compared AA lineages with alternative sexual controls to determine the consistency of the trend in the association of AA lineages or sexual controls with biotically and/or abiotically more heterogeneous environments (all trends were consistent over all alternative sexual controls, see Table 3).

**Table 3:** The heterogeneity of an environment of studied taxa

Ancient asexual taxon	Sexual control	Abiotically more homogenous than control	Biotically more homogenous than control
Bdelloidea	Monogononta	YesTend to be associated with marginal habitats and predominate there over sexual control (Pejler, 1995; Ricci, 1987; Ricci & Balsamo, 2000; Welch, Ricci, & Meselson, 2009), predominate over sexual control in polar habitats (Dartnall, 1983; Janiec, 1996; Jungblut, Vincent, & Lovejoy, 2012; Pejler, 1995; Sohlenius & Bostrom, 2005) + anhydrobiosis (Pilato, 1979; Ricci, 2001); predominate over sexual control in soil (Devetter & Scholl, 2014; Donner, 1975; Pejler, 1995; Scholl & Devetter, 2013); predominate over sexual control in hot springs at temperatures above 40°C (Issel, 1900, 1901; McDermott & Skorupa, 2011; Pax & Wulfert, 1941)	YesTend to be associated with marginal habitats and predominate there over sexual control (Pejler, 1995; Ricci, 1987; Ricci & Balsamo, 2000; Welch et al., 2009); aquatic representatives are exclusively benthic and sedentary in contrast to sexual control (Koste & Shiel, 1986; Ricci & Balsamo, 2000); predominate over sexual control in soil (Devetter & Scholl, 2014; Donner, 1975; Pejler, 1995; Scholl & Devetter, 2013); predominate over sexual control in polar habitats (Dartnall, 1983; Janiec, 1996; Jungblut et al., 2012; Pejler, 1995; Sohlenius & Bostrom, 2005); predominate over sexual control in hot springs at temperatures above 40°C (Issel, 1900, 1901; McDermott & Skorupa, 2011; Pax & Wulfert, 1941); absent in ancient lakes in contrast to sexual control (Martens & Schön, 2000; Schön & Martens, 2004); no typical predators and parasites (filtration, grazing etc.) in comparison with the sexual control (Ricci & Balsamo, 2000); getting rid of parasites (Wilson, 2011; Wilson & Sherman, 2010) and escaping from competitors, predators and parasites (Ladle, Johnstone, & Judson, 1993) via Bdelloidea‐specific anhydrobiosis; high tolerance to irradiation (Gladyshev & Meselson, 2008) and starving (Ricci & Perletti, 2006) because of Bdelloidea‐specific anhydrobiosis
Darwinuloidea	Cypridoidea	No DifferenceTend to be associated with marginal habitats, springs and interstitial (Pieri, Martens, Stoch, & Rossetti, 2009; Pinto, Rocha, & Martens, 2005; Schön, et al. 1998; Schön, et al. 2009) + torpor (Carbonel, et al. 1988; Delorme & Donald, 1969; Retrum, Hasiotis, & Kaesler, 2011), but the same applies to some degree also to the sexual control; Darwinuloidea does not dominate in hot springs over its sexual control (Brues, 1932; Jana & Sarkar, 1971; Klie, 1939; Külköylüoğlu, Meisch, & Rust, 2003; Moniez, 1893; Wickstrom & Castenholz, 1985)	YesTend to be associated with marginal habitats, springs and interstitial, but the same applies to some degree also to the sexual control (Pieri et al., 2009; Pinto et al., 2005; Schön et al., 1998, 2009); no typical predators and parasites (filtration) in comparison with the sexual control (Dole‐Olivier, et al. 2000); able to escape from competitors, predators and parasites because of torpor, but the same applies also to the sexual control (Carbonel et al., 1988; Delorme & Donald, 1969; Retrum et al., 2011); little parasitized, but the same applies to some degree also to the sexual control (Bruvo et al., 2011; Schön et al., 2009); aquatic representatives are exclusively benthic and sedentary in contrast to sexual control (Dole‐Olivier et al., 2000; Pokorný, 1965; Rossetti, Pinto, & Martens, 2011; Schön et al., 2009); riverine and lacustrine representatives predominantly inhabit hypoxic depths with few competitors, predators and parasites (Rossi, Todeschi, Gandolfi, Invidia, & Menozzi, 2002; Schön et al., 2009; Smith, Kamiya, & Horne, 2006); little predated (Ranta, 1979); highly tolerant to starving (Rossi et al., 2002); absent in ancient lakes with numerous competitors, predators and parasites in contrast to sexual control (Martens, 1998; Schön & Martens, 2004); does not dominate in extremely cold (Bunbury & Gajewski, 2009; Külköylüoğlu & Vinyard, 2000; McLay, 1978; Tudorancea, Green, & Huebner, 1979) or hot (Brues, 1932; Jana & Sarkar, 1971; Klie, 1939; Külköylüoğlu et al., 2003; Moniez, 1893; Wickstrom & Castenholz, 1985) environments in comparison with sexual control
Ancient asexual Oribatidae	Compared sexual Oribatidae	YesTend to be associated with soil in contrast to sexual controls and their predominance rises with the depth of soil horizon (Devetter & Scholl, 2014; Karasawa & Hijii, 2008; Krivolutsky & Druk, 1986; Maraun et al., 2009; Norton & Palmer, 1991); only few arboreal representatives in comparison with sexual controls (Karasawa & Hijii, 2008; Maraun et al., 2009); predominantly inhabit abiotically more stable forest soils in comparison with meadows (Krivolutsky & Druk, 1986; Siepel, 1994), but see also Devetter and Scholl (2014)	YesTend to be associated with soil in contrast to sexual controls and their predominance rises with the depth of soil horizon (Karasawa & Hijii, 2008; Maraun et al., 2009; Norton & Palmer, 1991); only few arboreal representatives (Karasawa & Hijii, 2008; Maraun et al., 2009); dominantly not typical predators and parasites (decomposition, fungivory, lichens, microorganisms), but the same applies also to the sexual controls (Norton & Behan‐Pelletier, 2009); predominantly inhabit stable environments with unstructured resources (Domes, et al. 2007; Maraun, et al. 2012); but do not prevail in the environment with less parasites and predators (Cianciolo & Norton, 2006)
Ancient asexual Endeostigmata	Compared sexual Endeostigmata	YesTend to be associated with soil, and, in contrast to sexual controls, especially its deep horizons (Darby, Neher, Housman, & Belnap, 2011; Neher, Lewins, Weicht, & Darby, 2009; Norton & Behan‐Pelletier, 2009; Norton et al. 1993; Oconnor, 2009; Walter, 2001, 2009); all hypothetical sister sexual lineages of *Alicorhagia* + *Stigmalychus* are much more ecologically disparate, including life in abiotically changeable environments (Darby et al., 2011; Neher et al., 2009; Norton & Behan‐Pelletier, 2009; Norton et al., 1993; Oconnor, 2009; Walter, 2001, 2009); ecological patterns analogical to Oribatidae but poorly explored (Norton & Behan‐Pelletier, 2009; Norton et al., 1993; Walter, 2009)	YesTend to be associated with soil, and, in contrast to sexual controls, especially its deep horizons (Darby et al., 2011; Neher et al., 2009; Norton & Behan‐Pelletier, 2009; Norton et al., 1993; Oconnor, 2009; Walter, 2001, 2009); dominantly not typical predators and parasites (decomposition, fungivory, microorganisms), but the same applies also to the sexual controls internal to the clade Endeostigmata (Walter, 2009); all hypothetical sister sexual lineages of *Alicorhagia* + *Stigmalychus* are much more ecologically disparate, including strategies with high degree of interspecific interactions (predators, parasites etc.) (Darby et al., 2011; Neher et al., 2009; Norton & Behan‐Pelletier, 2009; Norton et al., 1993; Oconnor, 2009; Walter, 2001, 2009); ecological patterns analogical to Oribatidae but poorly explored (Norton & Behan‐Pelletier, 2009; Norton et al., 1993; Walter, 2009)
Ancient asexual Trombidiformes	Compared sexual Trombidiformes	YesTend to be associated with soil, and, in contrast to sexual controls, especially its deep horizons (Bochkov & Walter, 2007; Darby et al., 2011; Kethley, 1989; Neher et al., 2009; Walter et al. 2009); all hypothetical sister sexual lineages are much more ecologically disparate, including life in abiotically changeable environments (Darby et al., 2011; Neher et al., 2009; Norton et al., 1993; Walter et al., 2009); ecological patterns analogical to Oribatidae but poorly explored (Norton & Behan‐Pelletier, 2009; Norton et al., 1993; Walter et al., 2009)	YesTend to be associated with soil, and, in contrast to sexual controls, especially its deep horizons (Bochkov & Walter, 2007; Darby et al., 2011; Kethley, 1989; Neher et al., 2009; Walter et al., 2009); no typical predators and parasites (decomposition, fungivory, microorganisms) in comparison with sexual controls (Darby et al., 2011; Neher et al., 2009; Norton et al., 1993; Walter et al., 2009); all hypothetical sister sexual lineages are much more ecologically disparate, including strategies with high degree of interspecific interactions (predators, parasites etc.) (Darby et al., 2011; Neher et al., 2009; Norton et al., 1993; Walter et al., 2009); ecological patterns analogical to Oribatidae but poorly explored (Norton & Behan‐Pelletier, 2009; Norton et al., 1993; Walter et al., 2009)
*Vittaria appalachiana*	Related sexual species	YesDistributed in higher latitude in comparison with sexual controls (Farrar, 1978, 1998), but associated exclusively with geologically and ecologically highly stable habitats (caves, excesses etc.) in contrast to sexual controls (Farrar, 1978, 1990, 1998); sexual controls are associated with exposed habitats (epiphytic on trees or decomposing wood) (Farrar, 1978, 1990; Farrar & Mickel, 1991)	YesAssociated with habitats characterized by minimal competition due to low light levels in contrast to sexual controls (Farrar, 1978, 1998); distributed in higher latitude in comparison with sexual controls (Farrar, 1978, 1998); highly vulnerable to parasitization and competition (Caponetti, Whitten, & Beck, 1982)
Ancient asexual *Timema*	Sister sexual species	No DifferenceNo difference in their phenotype in comparison with sexual controls (Sandoval, Carmean, & Crespi, 1998); areas of 2/3 AA species extend to higher latitudes than their sexual controls (Law & Crespi, 2002a,b), but other species of the genus (including short‐term asexual and sexual species) have even northern distribution (Law & Crespi, 2002b)	No Difference2/3 AA species have narrower food niche in comparison with sexual controls (Law & Crespi, 2002b); 2/3 AA species has separate areas from remaining species (Law & Crespi, 2002b; Sandoval et al., 1998) in contrast with sexual and short‐term asexual representatives of the genus (Law & Crespi, 2002b), but see Law and Crespi (2002a); areas of 2/3 AA species extend to higher latitudes than their sexual controls (Law & Crespi, 2002a,b), but other species of the genus (including short‐term asexual and sexual species) have even more northern distribution (Law & Crespi, 2002b)
Ancient asexual *Lasaea*	Sexual *Lasaea*	No DifferenceAncient asexual representatives have global distribution including high latitudes, whereas the distribution of sexual species is limited to the shores of Australia and Tasmania (Ó Foighil & Smith, 1995; Ó Foighil & Thiriot‐Quievreux, 1999; Taylor & Ó Foighil, 2000); associated with tidal zone, but the same applies both to AA and sexual *Lasaea* lineages (Morton et al. 1957); the ability to slow down metabolism and survive up to 12 days outside water, but the same applies both to AA and sexual *Lasaea* lineages (Morton et al., 1957)	No DifferenceAncient asexual representatives have global distribution including high latitudes, whereas the distribution of sexual species is limited to the shores of Australia and Tasmania (Ó Foighil & Smith, 1995; Ó Foighil & Thiriot‐Quievreux, 1999; Taylor & Ó Foighil, 2000); all AA representatives (but also one of two sexual species in the genus, *Lasaea colmani*) are exclusively benthic and directly developing without the presence of ancestral planktonic larva (Ó Foighil, 1989; Ó Foighil & Eernisse, 1988; Rosewater, 1975); associated with diverse community of invertebrates, cyanophyta and algae including algal species directly eroding Lasaea's shell, but the same applies both to AA and sexual *Lasaea* lineages (Morton et al., 1957); not typical predator or parasite (filtration), but the same applies both to AA and sexual *Lasaea* lineages (Morton et al., 1957)

Comparison of the biotic and abiotic heterogeneity of an environment inhabited by the studied ancient asexuals and their sexual controls. Detailed evaluation of the habitat heterogeneity is given in each pair to support our decision of which member of the pair inhabits a biotically or abiotically more heterogeneous environment.

### 2.2 Determination of environmental heterogeneity

Using relevant literary resources, we collected and analyzed data on the (biotically or abiotically more heterogeneous or homogeneous) character of environments inhabited by the studied groups (the data are summarized in Table 3). Biotic and abiotic environmental heterogeneity clearly have a nontrivial relationship to each other (see Discussion), but it is essentially possible to distinguish them.

It is also worth mentioning that an environmental heterogeneity, both biotic and abiotic, is an emergent property stemming from different factors and different adaptations in various AAs. An environmental heterogeneity of microscopic and macroscopic organisms, or more generally organisms living on different spatiotemporal scales, eventually organisms with completely different ecological strategies (terrestrial, benthic, planktonic, parasitic etc.), could not be quantified and rated on a single universal scale. However, individual AAs and their ecologically comparable sexual controls can be compared on the basis of particular factors that indicate a higher or a lower biotic or abiotic environmental heterogeneity of their particular environment. These factors are summarized in Table 4 (see Supporting information Materials and Methods for details). Resulting binary data were possible to analyze statistically.

**Table 4:** Factors determining biotic and abiotic environmental heterogeneity

Biotic heterogeneity		
Higher	Lower	References
Complex ecosystems with high degree of competition, predation, and parasitism; e.g. ancient lakes	Simple ecosystems low degree of competition, predation, and parasitism; for example, ephemeral, marginal, extreme habitats	Martens (1998); Martens and Schön (2000); Schön and Martens (2004) versus Bell (1982); Tobler, Schlupp, de Leon, Glaubrecht, and Plath (2007)
Unpredictable changes (predator‐prey cycles etc.)	Predictable changes (predator–prey cycles etc.)	Dawkins and Krebs (1979); Tokeshi (1999)
Tight and specific association with prey or host; e.g. predatory or parasitic lifestyle	Loose association with prey or host; for example, filtering or micropredatory lifestyle	Dawkins and Krebs (1979)
No adaptations to avoid competition, predation, and parasitism; e.g. durable resting stages	Adaptations to avoid competition, predation, and parasitism; for example, durable resting stages	Dawkins and Krebs (1979); Wilson (2011)
Planktonic or nektonic lifestyle	Benthic or sedentary lifestyle	Emiliani (1982, 1993a,b); Suttle, Chan, and Cottrell (1990); Bratbak, Egge, and Heldal (1993); Fuhrman (1999); Wommack and Colwell (2000); Fisher, Wieltschnig, Kirschner, and Velimirov (2003); Bettarel, Bouvy, Dumont, and Sime‐Ngando (2006); Filippini, Buesing, Bettarel, Sime‐Ngando, and Gessner (2006); Suttle (2005), Suttle (2007)
Not inhabiting soil, or only shallow soil horizons	Inhabitancy of soil, especially deep soil horizons	Wallwork (1970); Elliott, Anderson, Coleman, and Cole (1980); Murphy and Tate (1996); Drake, Choi, Haskell, and Dobbs (1998); Fisher et al. (2003); Lavelle and Spain (2003); Paul (2007)
Lower latitudes	Higher latitudes	Rohde (1986); Rohde and Heap (1998); Tokeshi (1999)
Shallower parts of water column	Deeper parts of water column	Etter, Rex, Chase, and Quattro (2005)
Abiotic heterogeneity		
Temporally changeable (on ecological timescales), spatially very heterogeneous, diverse and unstable habitats with unequally distributed resources; e.g. ephemeral and marginal habitats	Temporally stable, spatially homogeneous habitats with equally distributed resources; for example, caves, ground water reservoirs or soil environment (especially deeper soil horizons or soils of certain biomes)	Wallwork (1970); Farrar (1978); Farrar (1990); Farrar (1998); Krivolutsky and Druk (1986); Siepel (1994),; Siepel (1996); Pejler (1995); Lavelle and Spain (2003); Coleman, Crossley, and Hendrix (2004); Quesada et al. (2004); Paul (2007); Devetter and Scholl (2014)
Unpredictable changes	Predictable changes (e.g., cyclical)	Tokeshi (1999)
No adaptations to avoid temporary adverse abiotic conditions or enable migration; e.g. durable resting stages	Adaptations to avoid temporary adverse abiotic conditions or enable migration; for example, durable resting stages	Wilson (2011)
Extreme yet spatiotemporally changeable habitats; for example, nunataqs, desiccating ponds, bark surface	Temporally stable extreme habitats; e.g. hot springs or subsurface cavities	Bell (1982)
Lower latitudes and altitudes	Higher latitudes and altitudes	Hörandl (2006, 2009); Vrijenhoek and Parker (2009)
Freshwater habitats and coastal areas	Deeper parts of water column	Etter et al. (2005); Sheldon (1996)

Summary of factors that were evaluated to determine a higher or a lower environmental heterogeneity of AAs in comparison with their sexual controls. Note that the factors are not universal (a terrestrial organism cannot be benthic/nektonic etc.) and cannot be compared across all studies organisms. See Supporting information Materials and Methods for commentary and detailed description on how we determined biotic and abiotic environmental heterogeneity.

### 2.3 Statistics

Collected data were analyzed using the R v. 3.1.2 software environment (R_Core_Team, 2014). We used an exact test suggested by R. A. Fisher, specifically a one‐tailed binomial test, the only statistical technique which has a sufficiently high statistical power able to reject null hypothesis when we have extremely low N (theoretically a minimum of five). Using this technique, we tested three hypotheses: In case, the heterogeneity of habitats of AAs and their sexual controls differ, then asexual members of the pairs inhabit predominantly (1) biotically or abiotically, (2) biotically, and (3) abiotically more homogeneous environments.

Only in two AA groups (*Lasaea*,* Timema*), we were unable to identify any consistent differences in the heterogeneity of the environments inhabited by their sexual and asexual lineages. The most probable explanation of the absence of such a difference is a lack of empirical data. As the tested hypothesis makes predictions only about those pairs of species that differ in the heterogeneity of their habitats (and the binomial test analyses only binary variables, i.e., “less vs. more heterogeneous group,” not “equally heterogeneous groups,” see, e.g., McDonald, 2014), *Lasaea* and *Timema* were not included in the first round of our statistical analysis. The same applies for the abiotic heterogeneity of the environment of Darwinulidae.

To test the robustness of our results, we also ran more conservative second and third rounds of statistical analysis, including pairs with no reported difference in heterogeneity of habitats (1) as if they differed in the opposite direction than was predicted by our hypotheses and (2) as if they differed in the opposite direction but with only a 1/3 probability of positive outcome, that is, assuming a 2/3 probability of negative or indifferent result. The ecology, relevant adaptations, and environmental correlates of all eight pairs of AAs and their sexual controls were thoroughly examined in the exploratory part of the study, see Discussion.

## 3 RESULTS

We conclude that eight of the putative AA groups do fulfill our strict criteria of ancient asexuality: bdelloid rotifers (Bdelloidea), darwinulid ostracods (Darwinulidae), several lineages of oribatid mites (Oribatidae), several lineages of mites from the suborder Endeostigmata and order Trombidiformes, shoestring fern *Vittaria appalachiana* (Farrar & Mickel), three species of stick insects from the genus *Timema*, and several lineages of the bivalve genus *Lasaea*; see Table S1. Their sister or closely related ecologically comparable sexual groups were identified consequently with the help of relevant literature; see Table S2.

The comparison of the character of environments inhabited by the AAs and their sexual controls in the cases that differed in this factor showed that AAs inhabit biotically or abiotically (six of six, *p* = .016), biotically (six of six, *p* = .016), and abiotically (five of five, *p* = .031) more homogeneous environments. All these results are statistically significant. In cases in which the indifferent pairs were included in the analysis as negative observations, results became statistically insignificant (six of eight, *p* = .145; six of eight, *p* = .145; respectively, five of eight, *p* = .363). However, in cases in which the probability of positive result was set on 1/3 (leaving 2/3 probability of negative or indifferent result, however, see Discussion), results became marginally significant (six of eight, *p* = .02; six of eight, *p* = .02; respectively, five of eight, *p* = .088). Details of the results are summarized in Table 3 and the Supporting information Review of AA ecology.

In the exploratory part of the study, we searched for the traits that could be typical for ancient asexual organisms. We identified several properties and adaptations that are common to a considerable number of studied AAs, see Table 5. The most notable are durable resting stages, life in benthos and soil, and life in the absence of intense biotic interactions. On the other hand, widely discussed alternative means of genetic exchange and association with other species in a “domesticated” state were not found to be very frequent among putative AAs.

**Table 5:** Specific ecological properties and adaptations of AA taxa

	Alternative exchange of genetic information	Durable resting stages	Sedentary life and life in benthos	Life in the soil	Absence of life strategies with intensive biotic interactions
Bdelloidea	X	X	X	X	X
Darwinuloidea		X	X	X	X
Ancient asexual Oribatidae				X	X
Ancient asexual Endeostigmata				X	X
Ancient asexual Trombidiformes				X	X
*Vittaria appalachiana*					X
Ancient asexual *Timema*					X
Ancient asexual *Lasaea*		?	X		X

The distribution of specific environmental properties and organismal adaptations associated with studied AA taxa. Significance of these findings is discussed below.

## 4 DISCUSSION

In contrast with other comparative studies in the field, the presented one is based exclusively on the AA taxa. Moreover, biotic and abiotic environmental heterogeneity have been distinguished. We conclude that all six of the six AA groups that meet inclusion criteria of our initial statistical analysis (i.e., age >1 million years, reported differences in a heterogeneity of a habitat of AA and its sexual control) inhabit biotically more homogeneous environments and all five of the five‐ones inhabit abiotically more homogeneous environments when compared with their sexual controls. No AA group lives in an environment abiotically or biotically more heterogeneous than its sexual control.

In the cases excluded from the initial analysis (abiotic heterogeneity in Darwinulidae and both biotic and abiotic heterogeneity in *Timema* and *Lasaea*), it was not possible to distinguish whether the heterogeneity is lower in the AA group or in the sexual control. As expected, the observed results are not very robust due to an extremely low number of pairs of species for which the reliable ecological data are available (six). In the case of paired of species with no reported differences in heterogeneity of habitats were added to the analysis as negative observations, results became insignificant. Setting the probability of positive result to 1/3 (i.e., simulating 2/3 probability of negative or insignificant result) led to marginally significant results in the same case. However, this last test of the robustness of our results should be taken only as tentative because the direct assessment of the probability of indifferent result was beyond the possibilities of today's comparative studies. Nevertheless, even stepping aside from *p*‐values, our results show a clear trend of AA association with biotically and abiotically homogeneous environments, both in general and in comparison with their sexual controls.

The associations with biotically and abiotically more homogeneous environments overlap almost perfectly. Thus, the results of the comparative analysis clearly indicate that either the AA groups tend to be associated with overall (both biotically and abiotically) homogeneous environments or that these two types of heterogeneity are so strongly correlated that it is impossible to decide in favor of theories of sexual reproduction that stress the key role of biotic or abiotic heterogeneity. In general, our results obtained on AAs support, but of course do not prove, the hypotheses that consider both biotic and abiotic heterogeneities acting as one factor in their effect on organisms (Flegr, 2010, 2013; Roughgarden, 1991; Scheu & Drossel, 2007; Song et al., 2011; Williams, 1975 pp. 145–146, 149–154, 169).

Despite the widespread apprehension that the long independent evolution of AAs and their sexual controls would hamper any ecological comparative analysis of the type presented here (leading to the preference of studying young asexual lineages, see Introduction), we found that both groups usually inhabit quite similar and considerably homogeneous environments. This can, in fact, complicate analyses in the opposite way by making the determination of differences in a habitat heterogeneity impossible (as was the case of *Timema* and *Lasaea*, see Table 3). On the other hand, their common ancestor's association with the homogeneous environments could have been a preadaptation to the successful and long‐term transfer to asexual reproduction in the AAs. This tendency is obvious especially in Darwinuloidea–Cypridoidea, but it can also be seen in Bdelloidea‐Monogononta, Oribatidae, and Endeostigmata (see Table 3).

It is interesting in this regard that many contested AAs (see Table S1) also inhabit considerably homogeneous environments—for example,. arbuscular mycorrhizal fungi of the order Glomales (Croll & Sanders, 2009), tardigrades (Mobjerg et al., 2011; Pilato, 1979), nematode genus *Meloidogyne* (Castagnonesereno et al., 1993), ostracods *Heterocypris incongruens* (Ramdohr) and *Eucypris virens* (Jurine) (Butlin et al., 1998; Martens, 1998), bristle fern *Trichomanes intricatum* (Farrar) (Farrar, 1992), basidiomycete fungal families Lepiotaceae and Tricholomataceae (Currie, Mueller, & Malloch, 1999; Currie, Scott, Summerbell, & Malloch, 1999), ambrosia fungi Ophiostomatales (Farrell et al., 2001), or brine shrimp “*Artemia parthenogenetica*” (Bowen & Sterling) (Vanhaecke, Siddall, & Sorgeloos, 1984)—and their adaptations are similar to those of the AAs included in this study (see below).

### 4.1 What environmental properties and organismal adaptations are associated with AA taxa?

Besides the tendency to inhabit biotically and abiotically homogeneous environments, we discovered several properties and adaptations that are common to a considerable number of studied AAs, occur in AAs more often than in their sexual controls, and could be the particular adaptations enabling their long‐term survival in the environments mentioned above (see Table 5). The occurrence of these properties can, of course, be of little significance as we did not study their distribution throughout the near phylogeny. It is, however, interesting to mention them for the purposes of further research as universally distributed adaptations potentially connected to the mode of reproduction was not expected to be found in our sample because of markedly different ecological strategies of the studied AAs.

#### 4.1.1 Alternative exchange of genetic information

Alternative ways of exchange of genetic information could theoretically substitute sexual reproduction and thus were repeatedly proposed as the key adaptation to asexuality (Boschetti, Pouchkina‐Stantcheva, Hoffmann, & Tunnacliffe, 2011; Butlin, Schön, & Griffiths, 1998; Debortoli et al., 2016; Gladyshev & Meselson, 2008; Schwander, 2016). However, we identified this factor only once in the AAs included in our study (i.e., in one of eight cases), namely in Bdelloidea that experience intensive horizontal gene transfer (Boschetti et al., 2011; Debortoli et al., 2016; Gladyshev & Meselson, 2008). Another mechanism of genetic exchange, parasexuality (sensu Pontecorvo, 1954), was proposed in some contested ancient asexuals—Glomales (Croll & Sanders, 2009), Tricholomataceae and Lepiotaceae (Mikheyev, Mueller, & Abbot, 2006), and certain protists (Birky, 2009). However, considering only the well‐supported AAs, these mechanisms have limited distribution.

#### 4.1.2 Durable resting stages and subjectively homogeneous environment

The character of the environment is probably subjectively experienced rather differently by its inhabitants with their specific adaptations and by a human observer. In case that a particular organism reacts to the adverse change of environmental conditions by entrenching itself in the resting or durable persistent stages (e.g., anabiosis), then, as a result, it de facto does not subjectively experience the unfavorable conditions at all. Its objectively heterogeneous environment becomes subjectively much more homogeneous. It was even proposed that the presence of durable resting stages may, because of the reduced strength of selective pressures affecting these organisms in the long term, lead to an evolutionary stasis (Pilato, 1979).

This subjectivity of experienced environment probably addresses especially its abiotic factors, for example desiccation, which is survived in the anabiotic stages by Bdelloidea (Pilato, 1979; Ricci, 2001), or freeze and desiccation, which is survived in a state of torpor by Darwinulidae and some of their sexual relatives (Carbonel, Colin, Danielopol, Loffler, & Neustrueva, 1988). Similar durable stages could also be found in some contested AAs, namely “*Artemia parthenogenetica*” (Vanhaecke et al., 1984) and tardigrades (Mobjerg et al., 2011). Moreover, AA *Lasaea* is able to become mostly inactive and rests during the adverse conditions for some time as well (Morton, Boney, & Corner, 1957). On the other hand, at least in Bdelloidea, the anhydrobiosis may serve as the escape from biotic stresses too—especially parasites, both directly (the individual gets rid of parasites during desiccation) and indirectly (by enabling the escape from parasites in space and time), as was proposed by Wilson (2011). The distribution of durable resting stages among well‐supported AAs looks rather scarce (three of eight cases). However, these 2–3 groups comprise all studied AAs associated with significantly (objectively) abiotically heterogeneous habitats.

An underestimation of this phenomenon might be another reason why most researchers did not come to unambiguous conclusions in their comparative analyses of the ecology of sexual and asexual organisms. For example, many “extreme” environments may not be abiotically very homogeneous, whereas some environments that were designated as abiotically heterogeneous, for example, in the famous Bell's (1982) study (periodical ponds, dendrotelms etc.), could be very subjectively homogeneous for local inhabitants (e.g., anhydrobiotic Bdelloidea). After all, the heterogeneity of the environment depends on the adaptation of the observer, including the presence or absence of the durable stages.

#### 4.1.3 Sedentary life and life in benthos

At least three well‐supported AA groups (Bdelloidea, Darwinulidae, and *Lasaea*) are exclusively benthic or sessile in contrast to their sexual relatives (Dole‐Olivier, Galassi, Marmonier, & Des Chatelliers, 2000; Ó Foighil, 1989; Ricci & Balsamo, 2000). Some species of rotifer group Monogononta (sexual control for Bdelloidea) (Pejler, 1995) and ostracod group Cypridoidea (sexual control for Darwinulidae) (Martens, Schön, Meisch, & Horne, 2008) are planktonic; one of the two sexual lineages in genus *Lasaea* has planktonic larvae (Ó Foighil, 1988).

It was proposed that benthic or sessile life may significantly reduce the biotic heterogeneity of an environment affecting such organisms by effectively hampering and reducing the spread of parasites (Emiliani, 1993a,b), which is often considered to be one of the most dynamic and influential components of the organisms’ environment. It is true that, somehow paradoxically, paleontological studies (Jablonski, 1986) show increased extinction rates of species without planktonic larvae. However, the main reason for this is probably better colonizing abilities that are usually, but not always, associated with indirect development (Ó Foighil, 1989).

In a similar way to resting stages, the distribution of benthic or sedentary lifestyle among well‐supported AAs looks rather scarce on the first sight (three of eight cases). However, these three groups comprise all studied AAs that are (at least partially) associated with aquatic habitats. Moreover, it is interesting that numerous contested aquatic AAs are also exclusively benthic: flatworm *Schmidtea polychroa* (Schmidt) (Pongratz, Storhas, Carranza, & Michiels, 2003), New Zealand mudsnail *Potamopyrgus antipodarum* (Gray) (Neiman, Jokela, & Lively, 2005), and ostracods *Heterocypris incongruens* (Ramdohr) and *Eucypris virens* (Jurine) (Butlin et al., 1998; Martens, 1998).

#### 4.1.4 Life in the soil

Another adaptation widely distributed among AA groups is the inhabitancy of soil, especially deeper parts of the soil horizon. This tendency can be seen mainly in the AA mites from groups Oribatidae, Endeostigmata, and Trombidiformes, although their sexual relatives have some soil representatives too (Karasawa & Hijii, 2008; Maraun et al., 2009; Walter, 2009). Bdelloidea and Darwinuloidea tend to be associated with semiterrestrial habitats (Schön, Rossetti, & Martens, 2009). Moreover, AA Bdelloidea dominate among the soil rotifers above any of their sexual relatives (Pejler, 1995). Most representatives of Darwinulidae inhabit soil (respectively interstitial) too, although this applies also to some of their sexual relatives (Schön et al., 2009). Taken together, five of eight studied AA groups have numerous soil‐inhabiting representatives and show a tendency to inhabit soil.

Living in soil may, in a similar way to life in benthos, reduce the capacity of parasites to spread (sensu Emiliani, 1993a,b). The soil environment is three‐dimensional in its nature. Environments of surface organisms usually have some vertical dimension as well; however, this feature is pronounced much stronger in soil. Especially on smaller spatial scales characteristic for rotifers, ostracods, mites, fungi, and other putative AAs, the environment of soil organisms consists of tortuous system of pores and crevices. The shortest way from point A to point B in soil is only rarely a straight line. Under normal circumstances (i.e., population densities comparable to surface environments), this feature probably reduces any interactions of soil organisms and thus negatively affect parasitization, predation, and competition (Drake, Choi, Haskell, & Dobbs, 1998; Elliott, Anderson, Coleman, & Cole, 1980; Fisher, Wieltschnig, Kirschner, & Velimirov, 2003; Lavelle & Spain, 2003; Murphy & Tate, 1996; Paul, 2007; Pilato, 1979). However, it should be noted that this may change under high population densities (especially in surface layers of the soil or during some special occasions, such as periodic inflow of resources, and swarming) and therefore should be subject of further research. Besides, soil is an abiotically very stable environment shielding its inhabitants from fluctuations in temperature and humidity, as well as from UV radiation, and could be very favorable for asexuals also for this reason (Krivolutsky & Druk, 1986; Pilato, 1979; Siepel, 1994). In sum, the inhabitancy of soil habitats may eventually erase many of the hypothetical evolutionary advantages of sexuality and enable its inhabitants, or at least those who are not blocked to do so by some evolutionary constraints, to change their mode of reproduction to asexual. This, however, remains a speculation until a more extensive survey of soil organisms’ mode of reproduction is made.

Other explanations have also been proposed for the asexuals’ association with soil habitats. Oribatidae could suffer less intense selective pressures in the soil than in the arboreal environment where they have to respond to the coevolving lichens, their main food source (Maraun et al., 2009). Asexuality can be also more advantageous in soil because of the difficulties with seeking out sexual partners, less effective pheromone dispersal etc. (Karasawa & Hijii, 2008). Nevertheless, numerous contested AAs are soil inhabitants too: Glomales (Croll & Sanders, 2009), tardigrades (Jorgensen, Mobjerg, & Kristensen, 2007; Pilato, 1979), and *Meloidogyne* (Castagnonesereno et al., 1993).

It is also interesting in this regard that the selective pressures of biotic and abiotic environments in soil were proposed to be so weak they can ultimately (in a similar way to the presence of durable resting stages) lead to an evolutionary stasis (Pilato, 1979). This applies especially to Bdelloidea (Poinar & Ricci, 1992; Ricci, 1987) and Darwinulidae (Martens, Horne, & Griffiths, 1998; Schön, Butlin, Griffiths, & Martens, 1998; Schön et al., 2009) but, to some degree also to Oribatidae (Heethoff et al., 2007; Krivolutsky & Druk, 1986; Norton, 1994) and other AA mites (Norton, Kethley, Johnston, & O'Connor, 1993; Walter, 2009; Walter, Lindquist, Smith, Cook, & Krantz, 2009). Some evidence of evolutionary stasis can be seen in five of eight studied AA groups. Taking into account the contested AA groups, it can be found in Glomales (Redecker, Kodner, & Graham, 2000; Remy, Taylor, Hass, & Kerp, 1994) and tardigrades (Jorgensen et al., 2007; Pilato, 1979).

#### 4.1.5 Absence of life strategies with intensive biotic interactions

It is noticeable that there are practically no typical predators and parasites among the AAs we studied—this property is characteristic for all eight studied groups. Remarkably often they feed on dead organic matter or are autotrophic; parasites are almost absent, and in the case of a predatory lifestyle, they are phytophagous or filtering (see Table 3). One possible explanation is that they are unable to keep up in the coevolutionary race with their sexual hosts or prey. Thus, they may be successful in the long term, especially in the case of a predatory lifestyle, only if they adopt (or are preadapted to) such nonspecific ecological strategies. This, however, also applies to some of their sexual relatives and generally remains a hypothesis to be tested.

#### 4.1.6 Succumbing to domestication and delegation of concern for its own benefit to another biological entity

The tendency for asexual reproduction is particularly interesting in the contested AA fungi domesticated by ants (Formicidae) and bark beetles (Scolytinae). The ant symbionts are from the basidiomycete groups Tricholomataceae and Lepiotaceae (Mueller, Rehner, & Schultz, 1998), whereas bark beetles domesticate the ambrosia fungi of the ascomycete group Ophiostomatales (Farrell et al., 2001). The association is particularly close in the ants. They care for the fungi intensively, remove fungal predators and parasites, and the founding queen always carries filamentous bacteria, which synthetize an antidote against the main fungal pathogen—ascomycete *Escovopsis* (Currie et al., 1999, 1999) —not to mention the stable temperature and humidity in the nest. By doing so, they provide a very favorable, biotically and abiotically stable environments. Moreover, there is some evidence that they prevent fungi from their already minimal attempts at sexual reproduction. On the other hand, the situation may be more complicated because some of these fungi create sexual structures predominantly in the presence of ants (Mueller, 2002).

This phenomenon provides an alternative view on some aspects of human agriculture. Many plants raised by humans are sustained in agriculture by asexual reproduction (vegetative reproduction, fragmentation, or grafting), or at least self‐pollinating, which probably facilitates their breeding but increases their susceptibility to parasites and pathogens, the problem that must be continuously fought by their symbiont—humans (Flegr, 2002). Life in association with another organism that takes care of the symbiont can also be found in the contested AA group Glomales (Croll & Sanders, 2009) and various prokaryotic and eukaryotic endosymbionts, see, for example, Douglas (2010). However, it has not been found in any of the eight well‐supported AA groups we studied, and its effect on the long‐term maintenance of asexual reproduction thus remains only speculative.

## 5 CONCLUSIONS

The analytical part of this study, that is, the comparative analysis of the environment of AAs and their sexual relatives, mostly supported the hypothesis that AA groups are associated with overall (biotically and abiotically) more homogeneous environments in comparison with their sister or closely related ecologically comparable clades. This result was significant in two of three statistical tests we conducted, and only the most conservative approach did not come to a statistically significant result. This outcome consequently supported the theoretical concepts that postulate the essential advantage of sexual species in heterogeneous environments and consider the (biotic and abiotic, temporal and spatial) heterogeneity of the environment affecting the organisms to be one factor that can exhibit itself in many ways (Flegr, 2010, 2013; Roughgarden, 1991; Scheu & Drossel, 2007; Song et al., 2011; Williams, 1975 pp. 145–146, 149–154, 169). Particular ecological adaptations, from which durable resting stages, life in the absence of intense biotic interactions, and the association with soil and benthic habitats are most notable, might represent special cases of the general AAs’ association with overall homogeneous environments.

Therefore, the general notion that proposed theories of sexual reproduction (see Introduction) need not exclude each other, that the effects proposed by some or all of them might intertwine and affect individuals and evolutionary lineages simultaneously, or that they even may, ultimately, represent only different aspects of one more general explanation, seems to be supported by our results. Moreover, overall environmental heterogeneity, regardless of its complicated conceptualization and study, seems to be a suitable candidate for this hypothetical general explanation.

Most putative AA lineages are still critically understudied. One way of elaborating the foundations laid out by this study would be comparing the heterogeneity of environments in a broader spectrum of AA lineages as soon as more lineages are discovered or confirmed (e.g., the protist lineages proposed by Speijer et al., 2015). It would also be very desirable to investigate the ecology of *Lasaea*,* Timema*, and Darwinulidae in greater detail. Additionally, it would be appropriate to focus on the interaction of biotic and abiotic environmental heterogeneities and their effect on organisms. According to Flegr (2008, 2010, 2013), sexual groups should exhibit more pronounced evolutionary conservation of niches in comparison with asexuals—on the whole, they are expected to stick closely around the phenotype of their common ancestor. This hypothesis could be tested by comparing the variance of properties of individual species within an AA and its related sexual clade. It would be also possible to test whether particular sexual species are able to survive under a wider range of conditions of the heterogeneous environment due to their high genetic variability and hypothetical “elastic” reaction on selection, as was suggested by Flegr (2008, 2010, 2013).

## DATA AND MATERIALS

All data generated or analyzed during this study are included in this published article and its supporting information files.

## CONFLICT OF INTERESTS

The authors have no conflict of interests to declare.

## AUTHORS’ CONTRIBUTIONS

JT gathered the data, prepared the figures and tables and was the greatest contributor in writing the manuscript. JF contributed the analysis tools. Both JT and JF conceived and designed the study, analyzed the data and reviewed the drafts of the manuscript. All authors read and approved the final manuscript.
