# Supplementary material for: General environmental heterogeneity as the explanation of sexuality? Comparative study shows that ancient asexual taxa are associated with both biotically and abiotically homogeneous environments
Source: Ecol Evol. 2017 Dec 12;8(2):973–91. doi: 10.1002/ece3.3716 (PMC5773305; doi:10.1002/ece3.3716)
Supplement: Supplementary file 1 [file ECE3-8-973-s001.pdf]

Supporting information: 2 supplemental tables, Supplemental Material and Methods and Supplemental review of AA ecology

Table S1: Ancient asexual taxa

List of explicitly and implicitly proposed ancient asexual taxa along with their AA status according to the actual state of knowledge. Evidence for and against ancient asexuality was evaluated in each group and only well supported AAs were included in our study. The two criteria for inclusion were (1) obligately asexual reproduction, i.e. the absence of common or rare/cryptic sexual events, or derived forms of genetic exchange, for (2) at least 1 million years.

**Taxon**	**Ancient asexuality**	**Explicitly proposed in**	**Evidence against**	**Evidence for**
Confirmed ancient asexuals				
Rotifera: **Bdelloidea**	YES	Maynard Smith, 1986	**Domesticated horizontally transferable transposons** (Arkhipova & Meselson, 2005; Gladyshev & Arkhipova, 2007; Gladyshev, Meselson & Arkhipova, 2007); **enigmatic mentions of observed males** (Welch et al-, 2009); **evidence of allele sharing** (Signorovitch et al., 2015)	**Large and diverse clade** (Segers, 2007, 2008); **monophyletic** (Melone & Ricci, 1995; Wallace et al., 1996; Welch & Meselson, 2000); **asexual radiation** (Ricci, 1987; Birky et al., 2005; Pouchkina-Stantcheva et al., 2007); **frequency of hypothetical males would be so low they would be effectively asexual** (Birky, 2010); **very old** (Poinar & Ricci, 1992; Waggoner & Poinar, 1993; Welch & Meselson, 2000); **apomixis** (Hsu, 1956a, b); **accumulation of moderately deleterious mutations** (Barraclough et al., 2007); **Meselson effect** (Welch & Meselson, 2000; Welch et al., 2004b); **degenerated tetraploids** (Welch et al., 2004a, 2009; Welch et al., 2008; Hur et al., 2009); **specialization of collinear genes** (Pouchkina-Stantcheva et al., 2007; Welch et al., 2009); **functional haploidization** (Arkhipova & Meselson, 2000; Welch & Meselson, 2000; Gladyshev & Meselson, 2008); **unidentifiable homological pairs of chromosomes** (Welch & Meselson, 1998); **genome structure incompatible with meiosis** (Flot et al., 2013); **absence of vertically transferable transposons** (Arkhipova

				& Meselson, 2000, 2005); **allele sharing due to horizontal gene transfer** (Debortoli et al., 2016)
Arthropoda: Ostracoda: **Darwinulidae**	YES	Judson & Normark, 1996	**Vertically transferable transposons** (Schön & Arkhipova, 2006); **rare males** (Rossetti & Martens, 1996; Smith et al., 2006; Schön et al., 2009); **uncertain Meselson effect** (Schön et al., 1998); **slow speed of molecular evolution** (Gandolfi et al., 2001; Schön & Martens, 2003); **high homozygosity** (Martens, 1998); **low morphological and genetic diversity** (Rossetti & Martens, 1996, 1998; Schön et al., 1998)	**Large and diverse clade** (Martens et al., 2008; Martens & Savatenalinton, 2011); **monophyletic** (Schön et al., 2003, 2009; Yamaguchi & Endo, 2003; Wysocka et al., 2006; Yu et al., 2006; Yamada, 2007; Tinn & Oakley, 2008); **rare males spanandric** (Rossetti & Martens, 1996; Smith et al., 2006; Schön et al., 2009); **frequency of hypothetical males would be so low they would be effectively asexual** (Birky, 2010); **probably apomixis** (Butlin et al., 1998); **absence of automixis** (Schön et al., 1998); **absence of polyploidy** (Rossi et al., 1998); **absence of fossil males since the end of Permian /Triassic?/** (Molostovskaya, 2000; Martens et al., 2003); **millions of years old fossils classifiable into recent species and genera** (Straub, 1952; Abushik, 1990; Martens et al., 1997; Martens et al., 1998a; Martens et al., 2003; Smith et al., 2006); **very old according to the molecular clock** (Martens et al., 2005); **domesticated transposons** (Schön & Arkhipova, 2006); **genetic homogeneity proven not to be the consequence of automixis or recombination** (Schön & Martens, 2003); **unidentifiable homological pairs of chromosomes** (Tétart, 1978); **absence of hybridization among lineages** (Rossi et al., 1998)
Arthropoda: Acari: Oribatidae: **Nanhermanniidae, Malaconothridae, Trhypochthoniidae, Camisiidae and majority of Nothridae; Brachychthoniidae and Lohmanniidae**	YES	Judson & Normark, 1996	**Meselson effect not observed** (Schaefer et al., 2006); **rare males** (Palmer & Norton, 1990, 1991; Norton et al., 1993); **possibly revolution of sexuality** (Domes et al., 2007b)	**Large and diverse clades** (Norton et al., 1993; Subías, 2004; Norton & Behan-Pelletier, 2009); **asexual radiations** (Palmer & Norton, 1991); **rare males spanandric** (Taberly, 1988; Palmer & Norton, 1990, 1991, 1992; Norton et al., 1993); **Meselson effect not observed because of automixis with terminal fusion of gametes** (Taberly, 1987; Wrensch, Kethley & Norton, 1994; Schaefer et al., 2006) **and mostly inverted meiosis** (Wrensch et al., 1994; Heethoff, 2004; Laumann et al., 2008); **very old** (Krivolutsky & Druk, 1986; Maraun et al., 2003, 2004; Heethoff et al., 2007; Laumann et al., 2007) **+ not very good colonizers** (Skubala & Gulvik, 2005; Domes et al., 2007c; Cianciolo, 2009) **and biogeographic distribution corresponds to continental drift** (Hammer & Wallwork, 1979; Heethoff, 2004; Heethoff

				et al., 2007); **absence of sexual clades closely related to asexual lineages** (Norton et al., 1993; Maraun et al., 2004); **absence of recombination** (Palmer & Norton, 1992; Heethoff, 2004; Schaefer et al., 2006); **reevolution of sexuality is exceptional and uncertain** (Domes et al., 2007b)
Arthropoda: Acari: Endeostigmata: **Nematalycidae, Proteonematalycidae; Oehserchestidae, Granjeanicidae; *Alicorhagia, Stigmalychus***	YES	Judson & Normark, 1996	**Poorly understood** (Walter, 2009); **rare males** (Norton et al., 1993)	**Rare males are spanandric**; **analogies with asexual Oribatidae (frequency of males, their spanandric character, ecology, distribution, population genetics etc.)** (Norton et al., 1993)
Arthropoda: Acari: Trombidiformes: **Lordalycidae; Pomerantziidae**	YES	Judson & Normark, 1996	**Poorly understood** (Walter et al., 2009); **rare males** (Norton et al., 1993)	**Rare males are spanandric**; **analogies with asexual Oribatidae (frequency of males, their spanandric character, ecology, distribution, population genetics etc.)** (Norton et al., 1993)
Arthropoda: Phasmatodea: ***Timema*: *Timema tahoe*, *Timema monikensis*, *Timema genevieve***	YES	Sandoval, Carmean & Crespi, 1998	**Rare males** (Vickery & Sandoval, 2001; Law & Crespi, 2002a); **no signs of haploidization** (Schwander & Crespi, 2009a)	**Obligatory parthenogenesis** (Sandoval & Vickery, 1996; Sandoval et al., 1998; Law & Crespi, 2002a, b; Schwander & Crespi, 2009a); **apomixis** (Crespi & Sandoval, 2000; Law & Crespi, 2002a; Schwander & Crespi, 2009a; Schwander, Henry & Crespi, 2011); **asexual females do not mate with sexual males** (Sandoval et al., 1998); **rare males spanandric** (Law & Crespi, 2002a; Schwander & Crespi, 2009a); **degeneration of sexual traits in females** (Schwander et al., 2013); **recombination, haploid gametes and meiosis never observed** (Schwander et al., 2010, 2011); **degradation of chromosome pairing in *Timema genevieve*** (Schwander & Crespi, 2009a); **very old** (Law & Crespi, 2002a, b; Schwander et al., 2011); **extinct or undiscovered mother species improbable** (Sandoval et al., 1998); **Meselson effect** (Schwander et al., 2011); **slower mutation speed and more deleterious mutations in asexual lineages** (Schwander et al., 2011; Henry, Schwander & Crespi, 2012)
Mollusca: Bivalvia: ***Lasaea* except *Lasaea australis* and *Lasaea colmani***	YES	Ó Foighil & Smith, 1995	**Polyphyletic asexual lineages probably originated in hybridizations** (Thiriot-Quivreux, 1992; Ó Foighil & Smith, 1995; Taylor & Ó Foighil, 2000; Li et al., 2013); **sperm still able to bind to an egg and penetrate it** (Ó Foighil,	**Pseudogamous self-fertilizing hermaphrodites /autogynogenesis/** (Ó Foighil & Eernisse, 1988; Ó Foighil & Thiriot-Quiévreux, 1991, 1999; Ó Foighil & Smith, 1995; Taylor & Ó Foighil, 2000); **no outcrossing** (Ó Foighil, 1988); **male genetic material from sperm never transmit**

			1987); **the age estimated from biogeography could be an artefact if they are shown up to be good colonizers in ecological timescales** (Taylor & Ó Foighil, 2000)	**to offspring** (Ó Foighil & Thiriot-Quiévreux, 1991); **minimal male reproductive allocation, sperm are few and degenerated** (Ó Foighil, 1985; Beauchamp, 1986; McGrath & Ó Foighil, 1986); **no specialized sexual structures** (Ó Foighil & Eernisse, 1988); **almost certainly apomixis** (Ó Foighil & Thiriot-Quiévreux, 1991); **meiosis and segregation of chromosomes never observed** (Ó Foighil & Eernisse, 1988; Thiriot-Quivreux et al., 1988; Ó Foighil & Thiriot-Quiévreux, 1991); **adaptation and differentiation in asexuality** (Ó Foighil & Eernisse, 1988; Ó Foighil & Smith, 1996; Taylor & Ó Foighil, 2000; Li et al., 2013) **including ecological specializations** (Crisp & Standen, 1988; Tyler-Walters & Davenport, 1990); **polyploidy** (Ó Foighil & Smith, 1995); **variable count of chromosomes, frequent non-pairing chromosomes, impossibility of meiosis** (Thiriot-Quivreux et al., 1988; Ó Foighil & Thiriot-Quiévreux, 1991, 1999); **very old** (Ó Foighil & Smith, 1995, 1996; Ó Foighil & Jozefowicz, 1999; Li et al., 2013); **absence of cosmopolitan lineages, no rapid colonization** (Taylor & Ó Foighil, 2000); **comparable substitution speeds of sexual and asexual lineages** (Ó Foighil & Smith, 1995; Li et al., 2013); **colonization of large areas by rafting in direct-developing asexuals, long time ago when the ocean currents were different** (Ó Foighil & Jozefowicz, 1999); **repeated transitions between sexuality and asexuality, transfer of male genetic material or parallel neutral evolution highly improbable** (Ó Foighil & Smith, 1995); **undiscovered sexual lineages improbable** (Ó Foighil & Smith, 1995; Taylor & Ó Foighil, 2000)
Polypodiophyta: Vittariaceae: ***Vittaria appalachiana***	YES	Farrar, 1978	**Retrotransposons** (Docking et al., 2006); **rarely observed sporophytes** (Farrar, 1978; Caponetti et al., 1982)	**Exclusively asexual gametophyte** (Farrar, 1967); **asexual reproduction by gemmae and fission of thallus** (Farrar, 1974); **monophyletic** (Farrar, 1978, 1985, 1990); **rare sporophytes always abortive** (Farrar, 1978; Caponetti et al., 1982); **very old** (Docking et al., 2006) **+ most probably sister to some central- or south-American species** (Farrar, 1985) **+ sporophytes do not tolerate freezing, very limited migration and colonization, geographical distribution is relict of that of its mother species** (Farrar, 1978; Parks & Farrar, 1984; Farrar, 1990, 1998); **internally genetically uniform but disparate populations** (Farrar, 1978, 1985);

				**absence of gene flow** (Farrar, 1990); **adaptations in asexual state** (Farrar, 1985, 1990); **transposons probably domesticated** (Schön & Arkhipova, 2006)
Refused and contested ancient asexuals				
Polypodiophyta: Hymenophyllaceae: ***Trichomanes intricatum***	NO	Judson & Normark, 1996	**Overestimated age** (Ebihara et al., 2008)	**Obligately asexual** (Farrar, 1990, 1992)
Chordata: Lissamphibia: ***Ambystoma***	NO	Spolsky, Phillips & Uzzell, 1992	**Transfer of paternal genetic information from sperm in gynogenesis** (Hedges et al., 1992; Spolsky et al., 1992; Bogart et al., 2007)	**Gynogenetic clade old up to 3.5 MY** (Bi & Bogart, 2010; Bogart et al., 2007)
Chordata: Teleostei: ***Poeciliopsis* lineage MO/II**	NO	Judson & Normark, 1996	**Overestimated age** (Mateos & Vrijenhoek, 2002)	**Old obligately asexual lineage** (Quattro et al., 1992)
Arthropoda: Hemiptera: ***Trama***	NO	Moran, 1992	**Not obligately parthenogenetic – sexual populations** (Verma, 1969; Blackman, De Boise & Czylok, 2001)	**Obligately parthenogenetic populations** (Moran, 1992; Normark, 1999; Blackman, Spence & Normark, 2000)
Arthropoda: Hemiptera: ***Neotrama***	NO	Moran, 1992	**Not obligately parthenogenetic – sexual populations** (Verma, 1969; Normark & Moran, 2000)	**Obligately parthenogenetic populations** (Moran, 1992; Normark, 1999; Blackman et al., 2000)
Arthropoda: Hemiptera: ***Aspidiotus nerii***	NO	Provencher et al., 2005	**Uncertain age** (Provencher et al., 2005)	**Old obligately asexual lineage** (Provencher et al., 2005)
Arthropoda: Hemiptera: ***Rhopalosiphum padi***	NO	Simon et al., 1996	**Repeated generation of asexual lineages, hybridization, production of fertile males, gene flow, asexual lineages too young** (Simon et al., 1999; Delmotte et al., 2001; Delmotte et al., 2003)	**Old obligately asexual lineages** (Simon et al., 1996; Simon et al., 1999; Delmotte et al., 2001); **possibly Meselson effect** (Halkett et al., 2005)

Arthropoda: Hemiptera: **Adelgidae**	NO	Schwander & Crespi, 2009b	**Insufficient information** (Havill et al., 2007)	**Possibly old obligately asexual lineages** (Havill et al., 2007)
Arthropoda: Coleoptera: ***Aramigus***	NO	Judson & Normark, 1996	**Overestimated age** (Normark & Lanteri, 1998)	**Old obligately asexual lineage** (Normark, 1996)
Arthropoda: Coleoptera: ***Calligrapha***	NO	Gomez-Zurita et al., 2006	**Uncertain age** (Gomez-Zurita et al., 2006), **possible gene flow** (Montelongo & Gomez-Zurita, 2015)	**Old obligately asexual lineages** (Gomez-Zurita et al., 2006)
Arthropoda: Coleoptera: ***Otiorhynchus***	NO	Schwander & Crespi, 2009b	**Insufficient information** (Stenberg & Lundmark, 2004)	**Possibly old obligately asexual lineages** (Stenberg & Lundmark, 2004)
Arthropoda: Coleoptera: ***Naupactus leucoloma***	NO	Mayr, 1963	**Not obligately parthenogenetic – sexual populations** (Lanteri & Marvaldi, 1995)	**Obligately parthenogenetic populations** (Mayr, 1963)
Arthropoda: Phasmatodea: ***Bacillus atticus***	NO	Normark, Judson & Moran, 2003	**Uncertain age** (Mantovani et al., 2001)	**Old obligately asexual lineage** (Mantovani et al., 2001)
Arthropoda: Lepidoptera: **Naryciinae**	NO	Schwander & Crespi, 2009b	**Numerous recent transfers to asexuality, overestimated age** (Elzinga et al., 2013)	**Old obligately asexual lineages** (Grapputo et al., 2005)
Arthropoda: Ostracoda: ***Heterocypris incongruens***	NO	Chaplin & Hebert, 1997	**Overestimated age** (Tétart, 1978; Chaplin & Hebert, 1997; Martens et al., 1998b)	**Old obligately asexual lineage** (Chaplin & Hebert, 1997; Butlin et al., 1998)
Arthropoda: Ostracoda: ***Eucypris virens***	NO	Butlin et al., 1998	**Overestimated age** (Tétart, 1978; Martens et al., 1998b; Bode et al., 2010), **possible gene flow** (Schön et al., 2000)	**Old obligately asexual lineage** (Butlin et al., 1998; Bode et al., 2010)

Arthropoda: Anostraca: ***"Artemia parthenogenetica"***	NO	Judson & Normark, 1996	**Overestimated age** (Hebert et al., 2002), **not monophyletic** (Nascetti et al., 2003; Baxevanis et al., 2006; Muñoz et al., 2010)	**Old obligately asexual lineage** (Perez et al., 1994)
Arthropoda: Acari: ***Protogamasellus***	NO	Norton et al., 1993	**Arrhenotoky** (Afifi et al., 1986)	**Obligately parthenogenetic** (Norton et al., 1993)
Arthropoda: Acari: ***Gamasellodes***	NO	Norton et al., 1993	**Arrhenotoky** (Afifi et al., 1986)	**Obligately parthenogenetic** (Norton et al., 1993)
Arthropoda: Acari: ***Geholaspis***	NO	Norton et al., 1993	**Large proportion of males in some populations** (Gwiazdowicz & Klemt, 2004)	**Obligately parthenogenetic** (Norton et al., 1993)
Arthropoda: Acari: ***Trachytes***	NO	Norton et al., 1993	**Sexual** (Bloszyk & Szymkowiak, 1996; Bloszyk et al., 2004)	**Obligately parthenogenetic** (Norton et al., 1993)
Mollusca: Gastropoda: **Thiaridae**	NO	Morrison, 1954	**No larger lineage is obligately parthenogenetic – sexual populations** (Heller & Farstey, 1990)	**Obligately parthenogenetic populations** (Morrison, 1954)
Mollusca: Gastropoda: ***Campeloma parthenum***	NO	Normark et al., 2003	**Repeated transfers to asexuality, uncertain age of asexual lineages, possibility of hybridization** (Johnson & Bragg, 1999; Johnson, 2006)	**Old obligately asexual lineages** (Johnson & Bragg, 1999); **Meselson effect** (Johnson, 2006)
Mollusca: Gastropoda: ***Potamopyrgus antipodarum***	NO	Neiman, Jokela & Lively, 2005	**Unreliable age estimation** (Neiman et al., 2005); **obligatory asexuality questioned** (Neiman & Lively, 2005; Neiman et al., 2012)	**Old obligately asexual lineages** (Neiman & Lively, 2004; Neiman et al., 2005)
Gastrotricha: **Chaetonida**	NO	Stanley, 1979	**Facultative parthenogenesis** (Thorp & Covich, 1991; Ricci & Balsamo, 2000; Weiss, 2001)	**Obligately parthenogenetic** (Pilato, 1979; Stanley, 1979)

Tardigrada: ***Echiniscus testudo***	NO	-	**Overestimated age** (Jorgensen, et al., 2007)	**Old obligately asexual lineage** (Miller et al., 1999)
Nematoda: Heteroderidae: ***Meloidogyne***	NO	Judson & Normark, 1996	**Overestimated age** (Trudgill & Blok, 2001; Lunt, 2008)	**Old obligately asexual lineage** (Triantaphyllou, 1981; Castagnonesereno et al., 1993; Tigano et al., 2005)
Platyhelmintes: Turbellaria: ***Schmidtea polychroa***	NO	-	**Overestimated age** (Pongratz et al., 2003)	**Old obligately asexual lineage** (Pongratz et al., 2003)
Cnidaria: **Myxozoa**	NO	-	**Evidence of sexual process** (Morris, 2012)	**Sexual process never observed**
**Placozoa**	NO	Schurko, Neiman & Logsdon, 2009	**Evidence of sexual process** (Signorovitch et al., 2005)	**Sexual process never observed**
Glomeromycota: **Glomales**	NO	Judson & Normark, 1996	**The evidence of recombination and sexual process** (Vandenkoornhuyse et al., 2001; Gandolfi et al., 2003; Croll & Sanders, 2009; Halary et al., 2011); **meiotic genes** (Corradi & Lildhar, 2012; Tisserant et al., 2013)	**Very old obligately asexual lineage** (Remy et al., 1994; Rosendahl & Taylor, 1997; Stukenbrock & Rosendahl, 2005; Croll & Sanders, 2009); **vegetative incompatibility** (Giovannetti et al., 2003)
Basidiomycota: **Lepiotaceae**	NO	Judson & Normark, 1996	**The evidence of recombination** (Mueller, 2002; Doherty et al., 2003; Mueller et al., 2005; Mikheyev et al., 2006); **formation of sexual structures** (Mueller, 2002; Mueller et al., 2005); **absence of Meselson effect** (Mueller et al., 1998)	**Old obligately asexual lineages** (Chapela et al., 1994; Hinkle et al., 1994; Mueller et al., 1998)
Basidiomycota: **Tricholomataceae**	NO	Judson & Normark, 1996	**Evidence of recombination** (Mueller, 2002; Mueller et al., 2005; Mikheyev et al., 2006); **formation of sexual structures** (Mueller, 2002; Mueller et al., 2005); **ants are able to change among related strains of fungus** (Villesen et al., 2004)	**Old obligately asexual lineages** (Chapela et al., 1994; Hinkle et al., 1994; Mueller et al., 1998)

Ascomycota: **Ophiostomatales**	NO	Normark et al., 2003	**Uncertain age – horizontal transfer among bark beetles** (Farrell et al., 2001)	**Old obligately asexual lineages** (Farrell et al., 2001)
Ascomycota: ***Aspergillus fumigatus***	NO	-	**Evidence of sexual process** (O'Gorman et al., 2009)	**Sexual process never observed**
Ascomycota: ***Candida albicans***	NO	Schurko et al., 2009	**Evidence of recombination, probably facultative sexual** (Graser et al., 1996; Tibayrenc, 1997; Hull & Johnson, 1999; Tzung et al., 2001; Odds et al., 2007); **transposons** (Goodwin & Poulter, 2000)	**Parasexual cycle** (Bennett & Johnson, 2003; Forche et al., 2008); **clonal** (Graser et al., 1996; Tibayrenc, 1997)
Zygomycota: **Microsporidia (*Encephalitozoon cuniculi*)**	NO	Schwander & Crespi, 2009b	**Repeated recent loss of sexuality, overestimated age** (Ironside, 2007; Haag et al., 2013); **meiotic genes** (Biderre et al., 1999; Ramesh et al., 2005; Cuomo et al., 2012)	**Old obligately asexual lineages** (Tay et al., 2005)
Excavata: Trichomonadida: ***Trichomonas vaginalis***	NO	Schurko et al., 2009	**Meiotic genes, transposons** (Carlton et al., 2007; Malik et al., 2008); **evidence for recombination and sexual process** (Drmota & Kral, 1997; Hampl et al., 2001)	**Clonal** (Tibayrenc et al., 1990; Tibayrenc et al., 1991)
Excavata: **Diplomonadida (*Giardia intestinalis*)**	NO	Normark et al., 2003	**Evidence of sexual process** (Bernander et al., 2001; Morrison et al., 2007); **meiotic genes** (Ramesh et al., 2005; Malik et al., 2008); **population genetics signs of sex** (Cooper et al., 2007; Andersson, 2012)	**Obligately parthenogenetic** (Dacks & Roger, 1999); **domesticated transposons** (Arkhipova & Morrison, 2001)
Excavata: **Kinetoplastea (*Trypanosoma cruzi*, *Trypanosoma brucei*)**	NO	Schurko et al., 2009	**Meiotic genes, transposons** (Berriman et al., 2005; El-Sayed et al., 2005; Weedall & Hall, 2015); **evidence for recombination and sexual process** (MacLeod et al., 2005); **meiotic cycle and haploid gametes** (Peacock et al., 2011, 2014)	**Clonal** (Tibayrenc et al., 1990, 1991); **parasexual process** (Gaunt et al., 2003), **few or no recombination** (Oliveira et al., 1998)
Excavata: **Heterolobosea (*Naegleria gruberi*)**	NO	-	**Meiotic genes** (Fritz-Laylin et al., 2010)	**Sexual process never observed**

Excavata: **Jakobida**	NO	-	**Genes for plasmogamy and karyogamy** (Speijer et al., 2015)	**Sexual process never observed**
Excavata: **Malawimonadida**	NO	-	**Genes for plasmogamy and karyogamy** (Speijer et al., 2015)	**Sexual process never observed**
Alveolata: Dinoflagellata: ***Symbiodinium***	NO	-	**Meiotic genes** (Chi et al., 2014)	**Sexual process never observed**
Alveolata: **Apicomplexa (*Toxoplasma gondii*)**	NO	Schurko et al., 2009	**Evidence for recombination and sexual process** (Tibayrenc et al., 1990; Howe & Sibley, 1995; Grigg et al., 2001; Heitman, 2006; Khan et al., 2007); **meiotic genes** (Weedall & Hall, 2015)	**Clonal** (Tibayrenc et al., 1990, 1991; Howe & Sibley, 1995); **old** (Sibley & Boothroyd, 1992; Khan et al., 2007)
Stramenopila: **Eustigmatophyceae (*Nannochloropsis oceanica*)**	NO	-	**Related species sexual** (Radakovits et al., 2012)	**Haploid, sex process never observed, few mitotic genes** (Pan et al., 2011)
Stramenopila: **Parmales - Bolidophyceae**	NO	-	**Haploid and diploid stages of one group** (Kessenich et al., 2014)	**Sexual process never observed**
Rhizaria: **Foraminifera (*Reticulomyxa filosa*)**	NO	-	**Meiotic genes** (Glöckner et al., 2014)	**Sexual process never observed**
Chromista: **Cryptophyta (*Chroomonas acuta*; *Proteomonas sulcata*)**	NO	-	**Haploid gametes, syngamy** (Kugrens & Lee, 1988); **haploid and diploid stages** (Hill & Wetherbee, 1986)	**Sexual process never observed**
Chlorophyta: **Glaucophyta (*Cyanophora paradoxa*)**	NO	-	**Genes for plasmogamy and karyogamy** (Speijer et al., 2015)	**Sexual process never observed**

Chlorophyta: Prasinophyceae: ***Ostreococcus tauri***	NO	-	**Evidence of sexual process** (Grimsley et al., 2010)	**Sexual process never observed**
Chlorophyta: **Trebouxiophyceae (*Chlorella variabilis*)**	NO	-	**Evidence of sexual process** (Blanc et al., 2010); **meiotic genes** (Fucikova et al., 2015)	**Sexual process never observed**
Amoebozoa: **Entamoebida (*Entamoeba histolytica*)**	NO	Normark et al., 2003	**Evidence of sexual process** (Blanc et al., 1989); **meiotic genes** (Ramesh et al., 2005)	**Obligately parthenogenetic** (Dacks & Roger, 1999)
Opisthokonta: Ichthyosporea: ***Pseudoperkinsus tapestis***	NO	-	**Evidence of sexual process** (Marshall & Berbee, 2010)	**Sexual process never observed**
Opisthokonta: **Choanoflagelata (*Monosiga brevicolis*; *Salpingoeca rosetta*)**	NO	-	**Meiotic genes** (Carr et al., 2010); **retrotransposons** (Carr et al., 2008); **evidence of sexual process** (Levin & King, 2013)	**Sexual process never observed**
Opisthokonta: **Filasterea (*Capsaspora owczarzaki*)**	NO	-	**Meiotic genes** (Suga et al., 2013)	**Sexual process never observed**
Newly proposed ancient asexuals				
Ophistokonta: **Cristidiscoidea (*Fonticula alba*)**	?	Speijer et al., 2015	**Poorly understood**	**Sexual process never observed; genes for plasmogamy and karyogamy absent** (Speijer et al., 2015)
Ophistokonta: **Ichthyosporea (*Sphaeroforma arctica*)**	?	Speijer et al., 2015	**Poorly understood**	**Sexual process never observed; genes for plasmogamy and karyogamy absent** (Speijer et al., 2015)

**Apusomonadida (*Thecamonas trahens*)**	?	Speijer et al., 2015	**Poorly understood**	**Sexual process never observed; genes for plasmogamy and karyogamy absent** (Speijer et al., 2015)
Alveolata: Ciliata: **amicronucleate ciliates**	?	Speijer et al., 2015	**Poorly understood**	**Clonal, unable to undergo meiosis, lineages very old /?/** (Doerder, 2014); **"auto-recombination" of macronuclei** (Nowacki et al., 2008, 2011)
**Breviatea, Ancyromonadida, Mantamonadida, Rigifilida, Collodictyonida, Telonemia, Centrohelida, Palpitomonadea, Katablepharida, Picozoa**	??	Speijer et al., 2015	**Extremely poorly understood**	**Sexual process never observed**

Table S2: Sexual controls

List of sister or closely related, ecologically comparable, sexual taxa of the ancient asexuals included in our study. In those individual cases in which the phylogenetic relations between the sexual and asexual lineages were not entirely clear, we used the closest possible comparable clades, i.e. clades proven to be closely related and broadly comparable in terms of their ecology—aquatic for aquatic AAs, benthic for benthic AAs, terrestrial for terrestrial AAs, parasitic for parasitic AAs etc. In polyphyletic AA groups, monophyletic AA lineages and their sexual controls are identified.

Ancient asexual taxon	*Sexual control*	*Reasoning*
**Bdelloidea**	**Monogononta**	**Bdelloidea are monophyletic within Rotifera** (Melone & Ricci, 1995; Wallace et al., 1996; Welch & Meselson, 2000). **Phylogenetic relationships of the clades within Rotifera are unclear. Monogononta are ecologically comparable and closely related to Bdelloidea** (Garey et al., 1996; Welch, 2000; Herlyn et al., 2003; Garcia-Varela & Nadler, 2006; Sorensen & Giribet, 2006; Witek et al., 2008; Min & Park, 2009; Fontaneto & Jondelius, 2011; Lasek-Nesselquist, 2012).
**Darwinuloidea**	**Cypridoidea**	**Darwinuloidea consist of a single recent family Darwinulidae and are monophyletic within Ostracoda** (Schön et al., 2003, 2009; Yamaguchi & Endo, 2003; Wysocka et al., 2006; Yu et al., 2006; Yamada, 2007; Tinn & Oakley, 2008). **Phylogenetic relationships of the clades within Ostracoda are unclear. Cypridoidea are ecologically comparable and closely related to Darwinuloidea** (Scott, 1961; Maddocks, 1976; Martens et al., 1998a, b; Schön et al., 2003, 2009; Yamaguchi & Endo, 2003; Liebau, 2005; Wysocka et al., 2006; Yu et al., 2006; Tinn & Oakley, 2008).
**Oribatidae - Nanhermanniidae, Malaconothridae, Trhypochthoniidae, Camisiidae, larger part of Nothridae**	**Brachypylina**	**Nanhermanniidae, Malaconothridae, Trhypochthoniidae, Camisiidae and Nothridae are included into taxon Desmonomata, which is of unclear monophyly within Oribatidae. Phylogenetic relationships of the clades within Oribatidae are quite unclear. All clades within Oribatidae are ecologically comparable. Besides several isolated sexual species, Desmonomata are probably related to sexual crown group Brachypylina** (Norton et al., 1993; Norton, 1994; Judson & Normark, 1996; Normark et al., 2003; Domes et al., 2007a, b; Heethoff et al., 2009; Norton & Behan-Pelletier, 2009; Dabert et al., 2010).
**Oribatidae - Brachychthoniidae**	**Mesoplophoridae**	**Brachychthoniidae are probably monophyletic within Enarthronota. Phylogenetic relationships within Oribatidae are quite unclear. All clades within Oribatidae are ecologically comparable. Mesoplophoridae are closely related to Brachychthoniidae** (Norton et al., 1993; Norton, 1994; Judson & Normark, 1996; Normark et al., 2003; Domes et al., 2007a, b; Heethoff et al., 2009; Norton & Behan-Pelletier, 2009; Dabert et al., 2010).
**Oribatidae - Lohmanniidae**	**Remaining lineages of Enarthronota**	**Lohmanniidae are probably monophyletic within Enarthronota. Phylogenetic relationships within Oribatidae are quite unclear. All clades within Oribatidae are ecologically comparable. Lohmanniidae are basal lineage of Enarthronota and thus they can be compared only with its remaining lineages**

		(Norton et al., 1993; Norton, 1994; Judson & Normark, 1996; Normark et al., 2003; Domes et al., 2007a, b; Heethoff et al., 2009; Norton & Behan-Pelletier, 2009; Dabert et al., 2010).
**Endeostigmata - Nematalycidae and Proteonematalycidae**	**Micropsammidae**	**The sister group of Nematalycidae and Proteonematalycidae is Micropsammidae, which is also ecologically comparable** (Norton et al., 1993; Walter et al., 2009).
**Endeostigmata - Grandjeanicidae and Oehserchestidae**	**Terpnacaridae**	**The sister group of Grandjeanicidae and Oehserchestidae is Terpnacaridae, which is also ecologically comparable** (Norton et al., 1993; Walter et al., 2009).
**Endeostigmata – genera *Alicorhagia* and *Stigmalychus* (crown group of Alicorhagiidae)**	***Epistomalycus*/ Oribatidae + Astigmata**	**Genera Alicorhagia and Stigmalychus constitute the crown group of Alicorhagiidae. It is possible to compare them with the basal sexual genus of Alicorhagiidae (Epistomalycus). Alternatively, they can be compared with closely related groups Oribatidae + Astigmata** (Norton et al., 1993; Walter et al., 2009). **All mentioned clades are ecologically comparable. Both options were evaluated in the study.**
**Trombidiformes - Lordalycidae**	**Sphaerolichidae + Prostigmata**	**Lordalycidae are closely related to Sphaerolichidae and Prostigmata, which are also ecologically comparable** (Norton et al., 1993; Walter et al., 2009).
**Trombidiformes - Pomerantziidae**	**Pterygosomatoidea, Raphignathoidea, Cheyletoidea and Tetranychoidea/ Stigmocheylidae, Pseudocheylidae, Heterostigmata and Paratydelidae**	**Pomerantziidae are either basal in the clade including Pterygosomatoidea, Raphignathoidea, Cheyletoidea and Tetranychoidea or the clade including Stigmocheylidae, Pseudocheylidae, Heterostigmata and Paratydelidae** (Norton et al., 1993; Walter et al., 2009). **All mentioned clades are ecologically comparable. Both options were evaluated in the study.**
Vittaria appalachiana	***Vittaria linneata*/ *Vittaria graminifolia***	***Vittaria appalachiana* is a monophyletic clade** (Farrar, 1978, 1985, 1990). **Possible mother or sister lineages are *Vittaria linneata*** (Gastony, 1977), ***Vittaria graminifolia*** (Crane, 1997) **or (most probably) some yet unidentified neotropical species** (Farrar, 1985, 1990); **moreover, the origin of *Vittaria appalachiana* in interspecific hybridization cannot be ruled out** (Ebihara et al., 2009). **All mentioned clades are ecologically comparable. All options were evaluated in the study.**
Timema monikensis, Timema tahoe, Timema genevieve	***Timema cristinae, Timema bartmani, Timema podura***	**Phylogenetic relationships within the genus *Timema* are clear. Sister sexual lineage of *Timema monikensis* is *Timema cristinae*, sister sexual lineage of *Timema tahoe* is *Timema bartmani* and sister sexual lineage of *Timema genevieve* is *Timema podura*. *Timema monikensis* could even be internal lineage of recent *Timema cristinae*** (Law & Crespi, 2002a, b). **All mentioned species are ecologically comparable.**

**The majority of lineages of genus *Lasaea***	***Lasaea australis*, *Lasaea colmani***	**Only two diploid Australian lineages of genus *Lasaea*, *Lasaea australis* and *Lasaea colmani*, are sexual** (Ó Foighil & Smith, 1995; Ó Foighil & Thiriot-Quievreux, 1999). **But the asexual lineages of genus Lasaea almost certainly are not monophyletic and originated at least two times independently** (Thiriot-Quivreux, 1992; Ó Foighil & Smith, 1995; Taylor & Ó Foighil, 2000; Li et al., 2013). **Some of them are closer to *Lasaea australis*, whereas others to *Lasaea colmani*. Moreover, both sexual species probably do not have basal position within respective clades** (Taylor & Ó Foighil, 2000; Li et al., 2013). **This leaves us only with the option to compare all sexual with all asexual lineages of the genus. All mentioned lineages are ecologically comparable.**

Supplemental Materials and Methods: Details on determination of environmental heterogeneity

Biotic heterogeneity

We define biotically highly heterogeneous environments as those in which selective pressures affecting the offspring differ profoundly from those that previously affected their parents because of the coadaptation (or rather counter-adaptation) of interacting organisms. Thus, the most biotically heterogeneous environments are the habitats with a high degree of competition, predation, and parasitism. Biotic heterogeneity has both temporal (the coadaptation of interacting organisms) and spatial (e.g. the migration of the offspring to the areas with new competitors, predators, and parasites) dimensions. Changes in the biotic heterogeneity are essentially unpredictable, with the exception of some ecological cycles (e. g. host-predator or host-parasite cycles). In the latter case, environments with unpredictable changes were considered more biotically heterogeneous.

For example, the environment of organisms that live in tight association with other organisms is biotically very heterogeneous. This applies especially to predators and parasites that are forced to respond to the evolutionary counter-moves of their prey and hosts (Dawkins & Krebs, 1979). The more specific relationship with prey and hosts they have, the stronger the selective pressures of counter-adapting prey and hosts affect them (Dawkins & Krebs, 1979). Therefore, it is expected that organisms that use non-specific predatory strategies, e.g., filtering (especially if they filter both living organisms and dead organic matter), are under relatively weak selective pressure from their prey. Their environment is consequently biotically relatively homogenenous in this regard. On the other hand, the environment of organisms that are themselves under strong selective pressure of predators and parasites is highly biotically heterogeneous (Dawkins & Krebs, 1979). The environemnt of organisms that are not under strong selective pressures of predators and parasites for various reasons is biotically more homognenous in this regard (e.g. the environment of Darwinulidae, see Schön et al., 2009 or Bruvo et al., 2011). Another important component of environmental biotic heterogeneity is competition. The environments with complex ecosystems that are characterized by high a degree of competition, predation and parasitism among their inhabitants (e.g. ancient lakes, see Martens, 1998; Martens & Schön, 2000; Schön & Martens, 2004) are highly biotically heterogeneous for them. On the other hand, the environments with a low degree of competition, predation and parasitism (especially extreme habitats, e.g. the

environments of extremely high temperatures, see Tobler, 2007, or, for photosynthetic organisms, poorly lit environments, see Farrar, 1978, 1998), are biotically very homogeneous for their inhabitants. The vast majority of environments on Earth are thus somewhat biotically heterogeneous. In spite of that, we can find important exceptions. This factor of environmental heterogeneity is considerably weakened especially in extreme, ephemeral and marginal habitats that cannot sustain complex ecosystems because of their extreme conditions, rapid unpredictable changes, low carrying capacity and/or insufficient energy sources (see e.g. Bell, 1982).

An important factor that affects the biotic heterogeneity of environment the organisms experience is the way of life practiced by aquatic organisms. On the average, lesser biotic heterogeneity is experienced by benthic (or sedentary) organisms in comparison with planktonic aquatic organisms. The reason is that the latter are subject to fast and effective transmission of parasites and pathogens, especially viruses (see e.g. Suttle et al., 1990; Bratbak et al., 1993; Fuhrman, 1999; Wommack & Colwell, 2000; Suttle, 2005, 2007), because of the character of their environment—mixing of water masses lead to frequent encounters of various individuals (Emiliani, 1993a, b). Crucial difference of the resulting risks for benthic and planktonic organisms was pointed out by Emiliani (1982; 1993a). Emiliani (1993a) documented that benthic representatives of Foraminifera have lower risk of extinction in comparison with planktonic ones. The average length of existence of their benthic species was 20 million years, whereas planktonic species lasted only about 7 million years. The most probable explanation of this pattern is higher susceptibility of planktonic organisms to extinction caused by lethal parasitic, especially viral, infections. This finding gave rise to the viral theory of background extinctions (Emiliani 1993a, b). Evidence for the lower risks arising for benthic organisms from parasites and pathogens was also supported by other ecological studies. For example, Filippini et al. (2006) observed lower prevalence of individuals infected by viruses and consequent mortality among benthic bacteria (~0.03 %) in comparison with bacteria from water column (~6 %). Moreover, this pattern held despite much larger abundance of viruses in the sediment of studied temperate lake. Fisher et al. (2003) and Bettarel et al. (2006) came to very similar conclusions on the basis of studies of temperate oxbow lake and freshwater habitats in tropical Africa, respectively (but see Danovaro et al., 2008 for somewhat contrasting results from marine benthic sediment). Putting aside the limitations of parasite and pathogen transmission among benthic organisms, the protection of benthic organisms against viruses might be further enhanced by the

adsorption of viruses into organic and inorganic particles of sediment and the aggregation of benthic organisms (Filippini et al. 2006; Fisher et al., 2003). Whereas the extinction of whole species due to viral infection (eventually infection by another parasite or pathogen, e.g. fungus) is possible only under very limited conditions (see e.g. Buckwold, 1994, or de Castro & Bolker, 2005), local extinctions caused by pathogen or parasitic infections are probably quite common (Emiliani, 1982, 1993a; de Castro & Bolker 2005). The planktonic way of life thus probably considerably increases the selective pressures of parasites and pathogens and consequently the biotic heterogeneity of the environment.

The argument for lower biotic heterogeneity of benthic (or sedentary) organisms is possible to extend also to organisms that inhabit soil. From the viewpoint of the viral theory of background extinctions (Emiliani, 1993a, b), soil represents an environment that considerably impedes the spread of pathogens and parasites. Interactions with parasites and pathogens are very limited both in intensity and frequency due to the tortuous, i.e., multidimensional, character of soil matrix—it is best described as semi-discontinuous network of pores filled with air and/or water, or water films surrounding solid particles (Lavelle & Spain, 2003; for more information about the character of soil environment see also Wallwork, 1970; Coleman et al., 2004, or Paul, 2007). These features of soil matrix limit both the passive spread of parasites and pathogens and the frequency of encounters among their transmitters and organisms in general. The tortuous character of soil environments was stressed as a factor that limits the passive spread of viruses in benthic sediment by Fisher et al. (2003), whereas Murphy & Tate (1996) emphasized its limitations on the spread of bacteria. These observations are in agreement with Drake et al. (1998), who observed a negative correlation between the concentration of viral particles and sediment grain size. The sieving effect of the soil for organisms of various sizes is also commented by Paul (2007). Moreover, the tortuous character of soil impedes also active dispersion of organisms, e.g. when searching for prey (Elliott et al., 1980). Only a few larger organisms are able to effectively move larger distances within soil or even create their own habitats; movements of most organisms are locally constrained (Lavelle & Spain, 2003). Direct encounters between organisms, even organisms of the same species, are thus relatively infrequent. This leads, together with the limited spread of pheromones (Karasawa & Hijii, 2008), for example, to frequent transitions to indirect fertilisation with the help of deposited spermatophores (see e.g. Wallwork, 1970,

or Lavelle & Spain, 2003). The pattern of spatial autocorrelation of genetic lineages in soil communities, for example in rotifers (Robeson et al., 2011), further supports the limited dispersal abilities of soil organisms. The genetic diversity of various Bdelloidea lineages in soil is correlated only on small spatial scales (up to 54-133 m). Operational taxonomic units identified by Robeson et al. (2011) almost did not overlap above this distance. Habitats that distanced only tens to hundreds of meters were thus inhabited overwhelmingly by separate genetic lineages. Moreover, rotifer communities differed to a certain degree even in the smallest investigated distance of 16 cm (Robeson et al., 2011)[1].

Taken together, the tortuous character of the soil affects all soil organisms at various scales not only in terms of the reduced spread of parasites and pathogens, but also lower frequencies of encounters with predators and competitors. This leads to an overall reduction of biotic pressures in soil, which is further supported by the striking evolutionary stasis of many lineages of soil inhabiting organisms (Pilato, 1979). Moreover, species richness and population sizes, including parasites, predators and competitors, markedly decreases with the depth of soil horizon (Lavelle & Spain, 2003; Paul, 2007). Deep soil horizons are therefore even more abiotically homogeneous. The specific character of soil environment does not imply its general spatial homogeneity. On the contrary, soil is often spatially heterogeneous, especially on a larger scale (see e.g. Lavelle & Spain, 2003; Coleman et al., 2004, or Paul, 2007). It is the tortuous and multidimensional character of soil matrix that reduces biotic pressures affecting its inhabitants and makes this environment biotically very homogeneous.

The biotic heterogeneity of the environment the organisms experience might be reduced by the presence of durable resting stages. Organisms may get rid of parasites and pathogens, survive unfavourable environmental conditions, or colonize new habitats with naïve parasites, predators, pathogens and competitors in these stages (as do, for example, Bdelloidea—see Wilson, 2011). The geographical trend of decreasing biotic heterogeneity with increasing latitude might be expected on a global scale. Species diversity and ecosystem complexity decrease with distance from equator (Tokeshi, 1999). These events are coupled with a decreasing intensity

[1] High genetic diversity of Bdelloidea in gene cox1 is not very surprising in the light of severe DNA breaks that originate during anhydrobiosis, following repairs of these breaks and consequent intensive horizontal gene transfer (see e.g. Gladyshev et al., 2008).

of parasitization, abundance, prevalence and a relative diversity of parasites (Rohde, 1986; Rohde & Heap, 1998). An analogous trend of decreasing biotic heterogeneity with increasing depth might be expected in deeper parts of the water column for the same reasons (see e.g. Etter et al., 2005).

Abiotic heterogeneity

Abiotically highly heterogeneous environments are defined as those that are highly variable regarding changes of abiotic factors. They are diverse, unstable, and have unequally distributed resources. Again, the abiotic heterogeneity of the environment has both spatial (in the sense of variability) and temporal (in the sense of instability) dimensions. The offspring thus usually inhabit an environment different from that of their parents due to their dispersal in time and/or space. Changes in the abiotic environment could be predictable (e.g. cyclical) or unpredictable, and their intensity and frequency vary on different timescales. We are interested in ecological timescales in this study so we consider short-term unpredictably changing environments as the most abiotically heterogeneous.

Temporally and spatially highly changeable ephemeral and marginal habitats are especially abiotically heterogeneous environments (see e.g. Pejler, 1995). However, most of the surface terrestrial habitats are considerably abiotically heterogeneous. On the contrary, sheltered habitats such as caves, ground water reservoirs or soil environment are greatly abiotically homogenenous. Such environments protect their inhabitants from solar radiation and buffer short-term fluctuations in outer environment (e.g. changes of temperature and humidity), protecting their inhabitants from the direct impacts of such changes (Wallwork, 1970; Farrar, 1978, 1990, 1998; Krivolutsky & Druk, 1986; Lavelle & Spain, 2003; Devetter & Scholl, 2014). Most changes in soil matrix are much slower in comparison with surface habitats (Lavelle & Spain, 2003). The abiotic homogeneity of soil environment further increases with the depth of the soil horizon. For example, there is a specific depth of soil horizon in each geographical region under which the temperature is perienally stable, depending on its latitude, altitude and other climatic factors (Wallwork, 1970; Lavelle & Spain, 2003; Coleman et al., 2004; Paul, 2007). The buffering effect of soil on moisture fluctuations also increases with depth (see e.g. Quesada et

al., 2004). Moreover, soils of certain biomes (especially forest soils) are temporally abiotically more homogeneous than soils of other biomes (see e.g. Siepel, 1994, 1996).

Regarding aquatic environments, freshwater habitats and coastal areas are the most abiotically heterogeneous (Sheldon, 1996). The decrease of abiotic heterogeneity with increasing depth is also expected—water masses buffer surface environmental changes in a similar way to soil (see e.g. Etter et al., 2005). Certain extreme environments that are temporally stable (e.g. hot springs or subsurface cavities) are also very abiotically homogeneous (Bell, 1982), but this does not apply to all environments referred to as extreme.

In a similar way to the biotic heterogeneity of the environment, also the abiotic one might be reduced in the populations of organisms producing durable resting stages. Such an adaptation enables them to survive unfavourable fluctuations of an abiotic environment and promotes colonization of new habitats (see e.g. Wilson, 2011). On the other hand, mobility probably does not strongly affect the abiotic environmental heterogeneity that the organisms experience. Mobile organisms might hypothetically experience more different abiotic conditions in their life, but they can also easily stick with those most suitable for them. The geographical trend of increasing abiotic heterogeneity with increasing latitude and altitude might be expected to occur on global scale (Hörandl, 2006, 2009; Vrijenhoek & Parker, 2009). However, it is noteworthy that such a trend might be countered by an opposite trend of the decreasing biotic heterogeneity mentioned above in its effects on sexual and asexual species (and *vice versa*). All the expectations mentioned above need not apply absolutely, but may serve as useful leads in judging the environmental heterogeneity of various organisms if their peculiarities are taken into account.

Supplemental review of AA ecology

Bdelloidea

Bdelloidea is clade of hundreds of μm sized rotifers associated with semi-terrestrial, freshwater and soil environments (Ricci & Balsamo, 2000; Welch et al., 2009). Members of the ancient asexual group Bdelloidea inhabit most freshwater surface habitats, but are sometimes found also in brackish water and marginally in seawater (De Smet, 2002). Among freshwater environments, Bdelloidea dominate in biotically homogeneous marginal habitats with a reduced number of parasites, predators and competitors in contrast to its sexual control Monogononta (Ricci, 1987; Pejler, 1995; Ricci & Balsamo, 2000; Welch et al., 2009). They are exclusively benthic or sedentary (Koste & Shiel, 1986; Ricci & Balsamo, 2000), which indicates reduced biotic heterogeneity in contrast with sexual control. Monogononta are not only sedentary, but also obligately or facultatively planktonic and several of their species are clearly marine (Pejler, 1995). Concerning the biotically and abiotically homogeneous soil environment, Bdelloidea far outnumbers their sexual control there (Pejler, 1995). Bdelloidea overwhelmingly dominate among soil rotifers and rotifers associated with mosses (Bryophyta) and lichens (Pejler, 1995; Scholl & Devetter, 2013; Devetter & Scholl, 2014), reaching up to 95 % prevalence (Donner, 1975). Moreover, Bdelloidea are almost exclusive among rotifers in high mountain soil (at least in Himalayas—Devetter M., pers. comm. 2015). Abiotically homogeneous groundwater reservoirs are inhabited both by Monogononta and Bdelloidea. However, these habitats are poorly explored and observed species were probably transported there recently (Pejler, 1995). Bdelloidea are almost completely absent from biotically heterogeneous ancient lakes (e.g. Baikal) with complex ecosystems and intensive biotic interactions (predators, parasites and competitors). Sexual Monogononta overwhelmingly dominates here (Martens & Schön, 2000; Schön & Martens, 2004).

Anhydrobiosis, i.e. drying into the state of durable resting stages, is the key ability of Bdelloidea from the ecological viewpoint. It enables Bdelloidea to avoid unpredictable and unfavourable conditions in its surrounding environment. Bdelloidea often inhabit objectively abiotically very heterogeneous environments, but activate only under the most suitable conditions (Pilato, 1979; Ricci, 2001). Anhydrobiosis thus makes them experience only favourable conditions and makes their environment subjectively highly abiotically homogeneous. Moreover, anhydrobiosis enables them to get rid of parasites (Wilson & Sherman, 2010; Wilson, 2011) and

effectively disperse in space and time (Caceres & Soluk, 2002), which may also reduce the biotic heterogeneity of environment they experience. Anhydrobiosis is also tightly connected to other specific characteristics of Bdelloidea—exceptionally intensive horizontal gene transfer (Gladyshev, Meselson & Arkhipova, 2008; Debortoli et al., 2016), frequent repairs of DNA double-strand breaks and exceptional resistance to radiation (Gladyshev & Meselson, 2008) and starving (Ricci & Perletti, 2006). Intensive horizontal gene transfer is probably the reason for the relatively large genetic differences among clades of soil Bdelloidea that inhabit environments that distance from tens to hundreds of meters, as observed by Robeson et al. (2011). This alternative mode of genetic exchange was also proposed as an explanation for their ancient asexuality, substituting for their ability of genetic exchange during sexual processes (Boschetti et al., 2011; Gladyshev et al., 2008; Debortoli et al., 2016; Schwander, 2016)[2]. However, Bdelloidea are extremely phenotypically conservative (Pilato, 1979) despite their intensive horizontal gene transfers, which supports the primacy of homogeneous environment in the long-term maintenance of their asexuality. Sexual Monogononta does not have the ability of anhydrobiosis. They only produce mictic eggs at the end of the season, durable resting stages that serve for the survival of predictable periods of unfavourable conditions (Ricci, 2001). The character of the environment that they subjectively experience is thus much more biotically and abiotically heterogeneous.

It is noteworthy that Bdelloidea are ecologically more successful in thermally extreme environments. Both Monogononta and Bdelloidea are found in polar regions (Dartnall, 1983; Pejler, 1995; Sohlenius & Bostrom, 2005; Jungblut et al., 2012). However, Bdelloidea—especially *Philodina gregaria*—reaches much higher population densities and form growths sized up to several m^2 in these habitats (Dartnall, 1983; Pejler, 1995). Bdelloidea also reach much larger abundances than Monogononta in polar habitats (Janiec, 1996). These habitats are biotically very homogeneous because of reduced biotic interactions. They are also experienced as

[2] The role of horizontal gene transfer in bdelloid rotifers as a substitution for sexual processes remains intensively discussed. On the one hand, it was proposed that bdelloids in fact experience rare sex (Signorovitch et al., 2015; 2016), but on the other that the observed genetic exchange is best explained by intensive horizontal gene transfer (Debortoli et al., 2016; Flot et al., 2016). Due to the strong evidence supporting the absence of amfimixis in Bdelloidea (see table S1), we incline to think that Bdelloidea do not experience standard sexual processes (nevertheless, see Signorovitch et al., 2015; 2016; Debortoli et al., 2016; Flot et al., 2016). In either case, the rate of horizontal gene transfer in bdelloid rotifers is extraordinary high and probably tightly connected to their long-term survival in asexual state (Schwander, 2016).

very abiotically homogeneous by Bdelloidea because of their ability to survive adverse conditions in anhydrobiotic state. Bdelloidea are also more successful in comparison with their sexual control at high temperatures. Issel (1900, 1901) observed *Philodina roseola* in Italian hot springs at temperatures up to 46 °C. He concluded that *Philodina roseola* is specialized to high temperatures and dominates over sparse members of Monogononta above 40 °C. Pax and Wulfert (1941) reached similar conclusions in their research of central European hot springs. This pattern is further supported by environmental sequencing of hot springs in Alaska (McDermott & Skorupa, 2011). Despite the limitations of this study—for example, the authors could not distinguish the sequences of active living individuals from sequences of inactive individuals, dead specimens or contamination—the sequences of Bdelloidea, specifically *Philodinidae*, overwhelmingly dominated in springs with temperatures above 40 °C. The habitats of hot springs are abiotically stable and thus abiotically very homogeneous. Moreover, the number of competitors, predators and parasites decreases at high temperatures, which makes these environments biotically very homogeneous.

Bdelloidea and Monogononta predominantly feed by filtering surrounding water for small organic particles. Rarely, some sedentary representatives graze nutrients from nearby surfaces or drill through algal cell walls and suck its cytoplasm. However, there are no active predators among Bdelloidea in contrast to Monogononta (Ricci & Balsamo, 2000). The only single exception seems to be *Abrotrocha carnivora* (Ricci et al., 2001). None of two compared groups comprise parasites.

Taken together, Bdelloidea dominate over their sexual control in marginal habitats with reduced biotic heterogeneity. Anhydrobiosis shields its representatives from unfavourable conditions of their abiotic environment and consequent selection. Bdelloidea thus experience even abiotically homogeneous environment. Moreover, anhydrobiosis protects them to a certain degree even from the unfavourable consequences of intensive biotic pressures—it enables Bdelloidea to get rid of parasites and escape parasites, predators and competitors in time and space. Anhydrobiosis thus reduces even the experienced biotic heterogeneity of their environment. Bdelloidea dominate over their sexual control among rotifers inhabiting biotically and abiotically homogeneous soil environment. Bdelloidea are also ecologically very successful in biotically homogeneous polar regions with a low amount of biotic interactions (an environment which is for them also abiotically homogeneous due to anhydrobiosis) and abiotically stable hot springs

with reduced biotic pressures (abiotically and biotically homogeneous environments). Bdelloidea thus predominate over their sexual control even in objectively homogeneous environments. Cyclically sexual Monogononta dominate over Bdelloidea in water column and have numerous planktonic and sea representatives. Some of their species are predatory and thus experience stronger biotic pressures. They do not have the ability to undergo anhydrobiosis and thus live in subjectively much more heterogeneous environment than Bdelloidea. Monogononta also overwhelmingly prevail over Bdelloidea in ancient lakes with intensive biotic interactions. In conclusion, Bdelloidea are associated with biotically and abiotically more homogeneous environments than their sexual control Monogononta.

Darwinuloidea

Darwinulidae is the only extant member of superfamily Darwinuloidea. It is a group of hundreds of µm sized ostracods (Schön et al., 2009). Darwinulidae are present in most aquatic habitats including marine (see e.g. Martens et al., 1998a). In a similar way to Bdelloidea, Darwinulidae are also predominantly associated with biotically homogeneous marginal and semi-terrestrial habitats or springs and biotically and abiotically homogeneous interstitial and soil (Schön et al., 1998; Pinto et al., 2005; Pieri et al. 2009; Schön et al., 2009). However, the sexual control of Darwinulidae, Cypridoidea, is also present in the same environments and Darwinulidae does not seem to prevail there over its sexual control. Some representatives of Cypridoidea are planktonic (Pokorný, 1965), whereas all representatives of Bdelloidea are exclusively benthic or sedentary (Schön et al. 1998, 2009; Pinto et al. 2005; Pieri et al. 2009). This indicates a reduced abiotic heterogeneity of their environment.

Darwinulidae are able to survive unfavourable conditions (drought, low temperatures etc.) in an inactive state of torpor (Carbonel et al., 1988). This ability may be superficially analogical to anhydrobiosis of Bdelloidea in its ecological consequences, but does not enable Darwinulidae to survive such severe conditions. In any case, the environment is also experienced as subjectively abiotically homogeneous by Darwinulidae at least to some degree. The ability to undergo torpor may also outweigh the absence of durable eggs that are present in the sexual control (Horne, 1993). However, numerous representatives of Cypridoidea are also capable of torpor and this ability is thus not exclusive to AA Darwinulidae in contrast to Bdelloidea-Monogononta (Delorme & Donald, 1969;

Carbonel et al., 1988; Retrum et al., 2011). Moreover, even representatives unable to enter a state of torpor are able to survive short periods of drought (Retrum et al., 2011). A Darwinulidae-specific brooding pouch is also an important part of resistance to unfavourable conditions of biotic and abiotic environments. It protects offspring from fluctuations of abiotic environmental factors and predators and also facilitates the dispersal abilities of Darwinulidae (Martens, 1998). Its presence may decrease the experienced biotic and abiotic heterogeneity of the environment.

Darwinulidae are not predominantly associated with permanent bodies of water. However, the individuals that inhabit them prefer hypoxic depths with lower biotic pressures (Rossi et al., 2002; Smith et al., 2006; Schön et al., 2009). Such depths are also optimal for dispersion with the help of water birds, but the depth preference of Darwinulidae does not seem to be selected primarily for this reason. Despite the fact that Darwinulidae have numerous preadaptations hypothetically promoting its dispersal (see e.g. Delorme & Donald, 1969; Martens, 1998; Frisch et al., 2007; Retrum et al., 2011), they are probably not good colonizers (Malmqvist et al., 1997; Martens et al., 2008b). Another explanation, for example, the absence of predators, predators and competitors in hypoxic areas and consequently reduced biotic heterogeneity, seems more plausible. Darwinulidae are also nearly absent from ancient lakes with strong biotic pressures, which are dominated by sexual ostracods (Schön & Martens, 2004). A similar pattern also applies to groundwater (Schön & Martens, 2004; Martens et al., 2008a). These environments are highly biotically heterogeneous.

Darwinulidae are not more prevalent or ecologically successful in thermally extreme environments in comparison with their sexual control. The lower limits of their activity lie around 0 °C, which is similar to Cypridoidea (McLay, 1978; Tudorancea et al., 1979; Külköylüoğlu & Vinyard, 2000; Bunbury & Gajewski, 2009). The upper limits of their activity are inferior to those of Cypridoidea. Some species of Cypridoidea are specialized to high temperatures and activate even at 54 °C (Külköylüoğlu et al., 2003). Other Cypridoidea representatives were observed as ecologically successful at temperatures around 50 °C (Moniez, 1893; Brues, 1932; Klie, 1939; Jana & Sarkar, 1971; Wickstrom & Castenholz, 1985). Darwinulidae only rarely activate at temperatures above 35 °C (Brues, 1932; Reeves et al., 2007) and their upper thermal limit of activity lies around 40-50 °C (Menzel, 1923; Ponyi, 1992). They

are thus not associated with thermally more extreme biotically or abiotically homogeneous environments in comparison with their sexual control.

Another characteristic of Darwinulidae is that they are highly resilient to starvation (Rossi et al., 2002) and only rarely predated (Ranta, 1979). Moreover, ostracods in general are rarely and weakly parasitized (Schön et al., 2009; Bruvo et al., 2011), which may be an effective preadaptation to their frequent transitions to asexuality—young asexual lineages are common also in Cypridoidea. All of this indicates that they experience reduced biotic heterogeneity of their environment. The pattern of geographical parthenogenesis, i.e. increasing number of asexual lineages with latitude and altitude, is also often commented in ostracods. The distribution in higher latitudes and altitudes is associated with increased abiotic heterogeneity and decreased biotic heterogeneity. However, this pattern does not have general applicability in the compared taxa (Bell, 1982: 265-266; Schwander & Crespi, 2009). It applies only to certain species of Cyprididae that consist of sexual and asexual lineages (Horne et al., 1998).

Environmental correlates of AA Darwinulidae and its sexual control Cypridoidea are similar to Bdelloidea-Monogononta, but their differences are not nearly as distinct. Biotically and abiotically very homogeneous environments are inhabited both by the AA group and its sexual control in many ways. There are only few absolute differences between the compared groups. One reason for this "fogginess" might be the large disproportion in the number of species in the two compared clades. Sexual Cypridoidea comprise more than 1500 described species, whereas Darwinulidae are comprised by only about 40 (Martens et al., 1998a, b; Rossetti et al., 2011). Another possible reason is frequent transition to asexuality in certain species of Cypridoidea and consequent presence of many young asexual lineages (Martens, 1998). Such young asexual lineages may have transient advantage over sexual species in numerous environments that are also associated with the AA lineage (Martens, 1998).

Taken together, Darwinulidae tend to be associated with biotically homogeneous marginal habitats in a similar way to Bdelloidea-Monogononta. Moreover, they also experience such habitats as subjectively abiotically homogeneous because of their ability to undergo torpor. However, the same environments are equally inhabited by the sexual control Cypridoidea that is also capable of torpor. Darwinulidae thus does not seem to be associated with abiotically more homogeneous environments than their sexual

control. The same pattern applies to the association with abiotically and biotically homogeneous soil habitats or the absence of predatory and parasitic lifestyle and lower overall parasitization that mark more biotically homogeneous environment—it is characteristic for both compared groups. However, Darwinulidae are exclusively benthic or sessile in contrast to their sexual control, which is associated with a reduction of biotic heterogeneity. They are also nearly absent from the biotically complex environments of ancient lakes and are sparse in groundwater reservoirs that are both biotically heterogeneous. Species of Darwinulidae that inhabit stable water bodies such as lakes and rivers prefer hypoxic depths with reduced biotic stresses. Moreover, Darwinulidae are only under a weak pressure of predators and are resilient to starvation. All of that is associated with reduced biotic heterogeneity. In conclusion, ostracods are associated with markedly homogeneous environments in general. Darwinulidae in particular are associated with biotically more homogeneous environments in comparison with their sexual control. However, there is no clear difference in the abiotic heterogeneity of the environments Darwinulidae and their sexual control are associated with.

Oribatidae

Oribatidae is a diverse group of hundreds of μm sized mites (Norton & Behan-Pelletier, 2009). AA representatives of mites from the clade Oribatidae are predominantly associated with soil in contrast to their sexual controls. Moreover, their proportion increases with the depth of the soil horizon (Krivolutsky & Druk, 1986; Norton & Palmer, 1991; Karasawa & Hijii, 2008; Maraun et al., 2009; Devetter & Scholl, 2014). Soil is a biotically and abiotically more homogeneous environment in comparison with surface habitats. The upper layers of soil are abiotically relatively more changeable and characterized by more complex biotic interactions; biotic and abiotic heterogeneity further decreases with the depth of the soil horizon. This indicates an association of AA Oribatidae with biotically and abiotically homogeneous environments. On the other hand, sexual lineages of Oribatidae predominate in biotically and abiotically more heterogeneous arboreal habitats (Karasawa & Hijii, 2008; Maraun et al., 2009). Beyond that, AA representatives of Oribatidae are preferentially associated with abiotically more stable forest soils rather than changeable grassland soils (Krivolutsky & Druk, 1986; Siepel, 1994; but see also Devetter & Scholl, 2014 that proved stronger effect of seasonality in contrast to substrate and vegetation cover). There are almost no typical parasites and predators among AA Oribatidae, which may indicate a lower biotic

heterogeneity of their environment. They are saprophages, fungivores, or feed on algae and microorganisms. However, the same applies also to their sexual controls (Norton & Behan-Pelletier, 2009).

In the context of abovementioned characteristics, it is rather surprising that AA Oribatidae were not proven to be predominantly associated with biotically more homogeneous environments characterized by a reduced number of competitors and predators (Cianciolo & Norton, 2006). However, the study of Cianciolo and Norton (2006) might examine too narrow spectrum of their predators and parasites. Other experiments proved that AA representatives of Oribatidae are superior to sexual ones in abiotically homogenenous environment with unlimited unstructured resources. Sexual Oribatidae were more succesfull in the presence of structured resources (Domes et al., 2007c; Maraun et al., 2012).

The possible geographical parthenogenesis of Oribatidae, i.e. a higher proportion of asexual lineages in higher latitudes and altitudes characterized by higher abiotic heterogeneity and lesser biotic heterogeneity, remains questionable. Some studies supported this pattern (Behan-Pelletier, 1997), but other evidence is very unclear (Norton & Palmer, 1991; Cianciolo & Norton, 2006).

Taken together, AA lineages of Oribatidae are predominantly associated with biotically and abiotically homogeneous soil environment in contrast to their sexual controls. Moreover, the proportion of AA lineages increases with the depth of the soil horizon that is associated with further reduction of biotic and abiotic heterogeneity. They are less prevalent in arboreal environments that are more abiotically changeable and characterized by stronger biotic pressures, i.e. abiotically and biotically more heterogeneous. AA Oribatidae are also preferentially associated with abiotically stable forest soils in contrast to more changeable grassland soils. There are no typical predators and parasites among AA Oribatidae, but the same also applies to their sexual controls. A direct association with more biotically homogeneous environments in soil remains questionable. However, they are more successful in environments with unstructured resources in contrast to sexual representatives. The geographical parthenogenesis of Oribatidae, potentially associated with increase of abiotic heterogeneity and decrease of biotic heterogeneity with latitude and altitude, remains an open question. In conclusion, AA Oribatidae seems to be affected mainly by abiotic conditions of environment, but generally inhabit both biotically and abiotically more homogeneous environments in comparison with their sexual controls.

Other ancient asexual mites

Endeostigmata is a group of hundreds of μm sized mites predominantly associated with soil and especially extreme soil habitats—deep soil or sand horizons, microbial crusts, extremely salty habitats, seashores and desert soils (Walter, 2009). Such habitats are expected to be biotically more homogeneous. AA Endeostigmata tend to be associated with deep soil horizons in contrast to their sexual controls. All of their sexual controls are associated with abiotically and biotically more heterogenenous environments, especially abiotically relatively changeable upper parts of the soil with more biotic interactions. The only exception from this rule is the clade comprising of Grandjeanicidae and Oechserchestidae that do not exhibit any differences in heterogeneity of their environment in comparison with their sexual control (Norton et al., 1993; Walter, 2001, 2009; Neher et al., 2009; Norton & Behan-Pelletier, 2009; Oconnor, 2009; Darby et al., 2011). A predatory lifestyle is very rare among AA Endeostigmata and they are never parasitic. They are mostly saprotrophs, or feed on fungi and microorganisms, which makes their environment less biotically heterogeneous. However, this is also true for their sexual controls among Endeostigmata (Walter, 2001, 2009; Neher et al., 2009). A group consisting of genera *Alicorhagia* and *Stigmalychus* is an exception from this pattern, because it is much less phenotypically variable than any possible sexual control. Some of these controls are large lineages outside of Endeostigmata that comprise even typical predators and parasites (Norton et al., 1993; Walter, 2001, 2009; Neher et al., 2009; Norton & Behan-Pelletier, 2009; Oconnor, 2009; Darby et al., 2011). Taken together, their ecological patterns are similar to those of Oribatidae. They are just even less explored.

Trombidiformes is another diverse clade of hundreds of μm sized mites (Walter et al., 2009). Ecological patterns of AA lineages among Trombidiformes are analogical to those of Oribatidae and Endeostigmata (Kethley, 1989; Bochkov & Walter, 2007; Neher et al., 2009; Walter et al., 2009; Darby et al., 2011). However, they are also poorly explored. These AA mites are also predominately associated with biotically and abiotically homogeneous deep soil horizons and are not typically predators and parasites, which reduces the biotic heterogeneity of their environment (Norton et al., 1993; Neher et al., 2009; Walter et al., 2009; Darby et al., 2011). Their sexual controls from clade Prostigmata (and Sphaerolychidae in case of Lordalycidae), both alone and in any

combination (their phylogenetic position is unclear), are much more phenotypically disparate and comprise even typical predators and parasites (Neher et al., 2009; Norton et al., 1993; Walter et al., 2009; Darby et al., 2011).

Taken together, all abovementioned mite groups are poorly explored. However, if we can judge anything from the current knowledge of their ecology, it is that their AA lineages seem to be associated with biotically and abiotically more homogeneous environments. They are associated with soil and, in contrast to their sexual controls, especially deep soil horizons. Moreover, Trombidiformes, as well as *Alicorhagia* and *Stigmalychus*, are much less phenotypically variable than any of their sexual controls that comprise even predators and parasites and inhabit more biotically and abiotically heterogeneous environments. With the exception of Oehserchestidae that doesn't inhabit environments of different heterogeneity than their sexual control, we can conclude that AA mite lineages are always associated with more biotically and abiotically homogeneous environments than their sexual controls.

Vittaria

Vittaria appalachiana is an mm sized fern (Polypodiophyta) from the family Vittariaceae that forms growths up to several m^2 large (Farrar, 1978; Farrar & Mickel, 1991). It is unique among ferns by abandoning the stage of sexual gametophyte and surviving as purely an asexual sporophyte. *Vittaria appalachiana* is rather a group of related asexual lineages as opposed to a single species—individual populations are genetically and phenotypically variable (Farrar, 1978, 1985, 1990). *Vittaria appalachiana* is associated almost exclusively with caves, crevices and rock excesses that buffer fluctuations of outer temperature and humidity (Farrar, 1978, 1985, 1990). Populations of *Vittaria appalachiana* thus inhabit geologically and ecologically stable habitats (Farrar, 1998) that are characterized by low abiotic heterogeneity. Moreover, extremely low levels of light in its habitats lead to a strong reduction of interspecific competition (Farrar, 1978, 1998) and lower biotic heterogeneity. *Vittaria appalachiana* also inhabit higher latitudes in comparison with any proposed sexual control (Farrar, 1998, 1978; Farrar & Mickel, 1991), which could lead to a reduction of biotic heterogeneity. A possible co-occurring increase of abiotic heterogeneity with latitude probably does not affect *Vittaria appalachiana* strongly because of its buffering habitats. The association of *Vittaria appalachiana* with biotically homogeneous environments is further supported by the fact that individual plants are highly susceptible to parasitization and competition (as well as antibiotics and

sterilisation), as was observed during attempts to grow them in a laboratory (Caponetti et al., 1982). Its sexual controls do not exhibit these characteristics. Sporophytes of all proposed sexual controls grow epiphytically on decaying wood or trees in lower latitudes. Their habitats are thus more exposed to abiotic conditions of environment, biotically and abiotically more heterogeneous (Farrar, 1978, 1990; Farrar & Mickel, 1991).

Taken together, caves and rock overhangs inhabited by *Vittaria appalachiana* in contrast to all proposed sexual controls are geologically and ecologically stable, maintain stable level of humidity and protect their inhabitants from fluctuations of temperature. These habitats are thus abiotically very homogeneous. Extremely low levels of light in these habitats also strongly reduce any interspecific competition. This leads, as well as higher latitudes inhabited by *Vittaria appalachiana*, to lower biotic heterogeneity of its environment in comparison with any sexual control. Moreover, the individuals of *Vittaria appalachiana* are highly susceptible to infections and are not good competitors. This also points to their association with biotically homogeneous environments. In conclusion, *Vittaria appalachiana* is associated with more biotically and abiotically homogeneous environment than any of its proposed sexual controls.

Timema

Timema is a genus of cm sized herbivorous phasmatodean (Phasmatodea) insects that inhabit chaparral vegetation of western pars of North America (Vickery, 1993; Sandoval et al., 1998). Sexual, young sexual and AA lineages of the phasmatodean (Phasmatodea) genus *Timema* do not differ much in their ecology. Two of three AA species have narrower food spectra in comparison with their sexual controls. This could hypothetically lead to decreased competition and consequently a biotically more homogeneous environment. However, a third AA species, *Timema tahoe*, share the same host plant as its sexual control *Timema bartmani* (Law & Crespi, 2002b). None of the AA species are more specialized in comparison with their sexual control. In fact, they are markedly similar in terms of their phenotype (Sandoval et al., 1998). The areas of AA *Timema* species are usually geographically separated from areas of other members of the genera, mostly by a great distance (Sandoval et al., 1998; Law & Crespi, 2002b). This may lead to decreased interspecific competition with other members of the genera (especially species with the same or overlapping food niche)

and consequentially a decrease of the biotic heterogeneity of their environment. However, the areas of AA *Timema monikensis* and its sexual control *Timema chumash* overlap (Law & Crespi, 2002a), casting doubt on this possibility. On the other hand, young asexual species of the genera usually border and partially overlap their areas with sexual species of *Timema*, including their sister species (Law & Crespi, 2002b). Young asexual species of *Timema* also inhabit higher latitudes in comparison with their sexual relatives, which may indicate abiotically more heterogeneous but biotically more homogeneous environment. However, this pattern does not apply to AA species of *Timema* (Law & Crespi 2002a, b). Two of three AA species have markedly northern areas in comparison with their sexual controls. However, other species of *Timema*, including sexual ones, are distributed into even higher latitudes (Law & Crespi, 2002b).

Taken together, it doesn't seem that the environment inhabited by AA members of *Timema* is biotically or abiotically more homogeneous or heterogeneous in comparison with their sexual controls. Some indices, e.g. the separation of areas populated by AA members of the genera from areas populated by other members, point to reduced interspecific competition and possibly a reduced biotic heterogeneity of their environment. The distribution of AA timemas in higher latitudes in comparison with sexual controls may have similar consequences. However, possible indices of differences among the environmental heterogeneity of AA members of *Timema* and their sexual controls are highly unclear. In conclusion, available evidence is insufficient to identify any consistent differences in biotic and abiotic heterogeneity of the environment of AA members of *Timema* and their sexual controls.

Lasaea

Lasaea is a genus of mm sized intertidal bivalves (Bivalvia) (Morton et al., 1957). Its AA representatives have global distribution, whereas sexual controls are limited only to the shores of Australia and Tasmania (Ó Foighil & Smith, 1995; Ó Foighil & Thiriot-Quievreux, 1999; Taylor & Foighil, 2000). AA representatives of *Lasaea* are thus distributed to higher latitudes with hypothetically a reduced biotic heterogeneity and an increased abiotic heterogeneity. However, they are present also in lower latitudes. The main reason for this geographical pattern is thus probably not the hypothetical difference in environmental heterogeneity, but the excellent dispersal ability of directly developing AA members of *Lasaea* (Ó Foighil, 1989). However, one of two sexual lineages of

this genus, *Lasaea colmani*, also has direct development (Ó Foighil & Smith, 1995). It is thus difficult to make any conclusions on the basis of the association between their ancient asexuality with their direct development.

Lasaea is not associated with environments of a distinctly homogeneous character. It is associated with areas of tidal zone between the mean high water spring and low water spring tides. Its individuals are associated especially with shallow crevices and shaded stone recesses, empty barnacles and tufts of tidal vegetation (Morton et al., 1957). This boundary ecosystem is abiotically rather changeable, which may lead to decreased competition, predation and parasitization. However, *Lasaea* do not greatly experience reduced biotic pressures. Its individuals are associated with numerous marine plants, isopods (Isopoda), springtails (Collembola), mites (Acari), beetles (Coleoptera), pseudoscorpions (Pseudoscorpiones) and gastropods (Gastropoda). They are also accompanied by other bivalves in the lower parts of tidal zone (Morton et al., 1957). Amenzalous filamentous cyanobacteria and green filamentous algae (e.g. *Entocladia*) often grow on their shells and erode them (Morton et al., 1957). Species of genus *Lasaea* feed by filtering detritus and are not typical predators and parasites. However, these characteristics applies both to their AA and sexual lineages (Morton et al., 1957).

It is noteworthy that both AA and sexual lineages of *Lasaea* are not able to breath atmospheric oxygen in contrast to numerous tidal invertebrates. They tightly close their shells in unfavourable conditions (e.g. drought) and become inactive. Individuals of *Lasaea* are able to survive up to several days in this inactive state (according to Morton et al., 1957, up to 12 days). This ability primarily serves for their survival in the changeable conditions of tidal zone. However, it may also enable *Lasaea* to activate only under favourable conditions and consequently make its environment subjectively more homogeneous. Thus, it may be a preadaptation analogical to the anhydrobiosis of Bdelloidea or the torpor of Darwinulidae. However, thorough exploration of the hypothetical differences between AA and sexual lineages of Darwinulidae is needed to verify this hypothesis.

Taken together, the situation in genus *Lasaea* is the most complicated of all the studied AA groups from the viewpoint of present comparative study. *Lasaea* is highly understudied in this regard. Published literature does not offer enough information to investigate possible ecological differences among its AA lineages and sexual controls. In conclusion, available evidence is insufficient

to identify any consistent differences in the biotic and abiotic heterogeneity of the environment of AA members of *Lasaea* and their sexual controls.

References

Abushik A. 1990. *Manual of the microfauna of the USSR*. Leningrad, USSR: VSE-GEI, NEDRA.

Adl SM, Simpson AG, Lane CE, Lukeš J, Bass D, Bowser SS, Brown MW, Burki F, Dunthorn M, Hampl V, Heiss A, Hoppenrath M, Lara E, le Gall L, Lynn DH, McManus H, Mitchell EAD, Mozley-Stanridge SE, Parfrey LW, Pawlowski J, Rueckert S, Shadwick L, Schoch CL, Smirnov A, Spiegel F. 2012. The revised classification of eukaryotes. *Journal of Eukaryotic Microbiology* 59: 429-514.Afifi A, Hassan M, Nawar M. 1986. Notes on the biology and feeding habits of *Protogamasellus minutus* (Hafez, El-Badry & Nasr (Acari: Gamasida: Ascidae). *Bulletin of the Entomological Society of Egypt* 66: 251-259.

Andersson J. 2012. Double peaks reveal rare diplomonad sex. *Trends in Parasitology* 28: 46-52.

Arkhipova I, Meselson M. 2000. Transposable elements in sexual and ancient asexual taxa. *Proceedings of the National Academy of Sciences of the United States of America* 97: 14473-14477.

Arkhipova I, Meselson M. 2005. Diverse DNA transposons in rotifers of the class Bdelloidea. *Proceedings of the National Academy of Sciences of the United States of America* 102: 11781-11786.

Arkhipova I, Morrison H. 2001. Three retrotransposon families in the genome of *Giardia lamblia*: Two telomeric, one dead. *Proceedings of the National Academy of Sciences of the United States of America* 98: 14497-14502.

Barraclough TG, Fontaneto D, Ricci C, Herniou EA. 2007. Evidence for inefficient selection against deleterious mutations in cytochrome oxidase I of asexual bdelloid rotifers. *Molecular Biology and Evolution* 24: 1952-1962.

Baxevanis A, Kappas I, Abatzopoulos T. 2006. Molecular phylogenetics and asexuality in the brine shrimp *Artemia*. *Molecular Phylogenetics and Evolution* 40: 724-738.

Beauchamp K. 1986. Reproductive ecology of the brooding, hermaphroditic clam *Lasaea subviridis*. *Marine Biology* 93: 225-235.

Behan-Pelletier V. 1997. Oribatid mites of the Yukon. In: H.V. Danks HV, Downes JA, eds. *Insects of the Yukon*. Ottawa: Biological Survey of Canada (Terrestrial Arthropods), 115-149.

Bell G. 1982. *The masterpiece of nature: the evolution and genetics of sexuality*. London, UK: Croom Helm.

Bennett R, Johnson A. 2003. Completion of a parasexual cycle in *Candida albicans* by induced chromosome loss in tetraploid strains. *Embo Journal* 22: 2505-2515.

Bernander R, Palm J, Svard S. 2001. Genome ploidy in different stages of the *Giardia lamblia* life cycle. *Cellular Microbiology* 3: 55-62.

Berriman M, Ghedin E, Hertz-Fowler C, Blandin G, Renauld H, Bartholomeu D, Lennard N, Caler E, Hamlin N, Haas B, Bohme W, Hannick L, Aslett M, Shallom J, Marcello L, Hou L, Wickstead B, Alsmark U, Arrowsmith C, Atkin R, Barron A, Bringaud F, Brooks K, Carrington M, Cherevach I, Chillingworth T, Churcher C, Clark L, Corton C, Cronin A, Davies R, Doggett J, Djikeng A, Feldblyum T, Field M, Fraser A, Goodhead I, Hance Z, Harper D, Harris B, Hauser H, Hostetter J, Ivens A, Jagels K, Johnson D, Johnson J, Jones K, Kerhornou A, Koo H, Larke N, Landfear S, Larkin C, Leech V, Line A, Lord A, MacLeod A, Mooney P, Moule S, Martin D, Morgan G, Mungall K, Norbertczak H, Ormond D, Pai G, Peacock C, Peterson J, Quail M, Rabbinowitsch E, Rajandream M, Reitter C, Salzberg S, Sanders M, Schobel S, Sharp S, Simmonds M, Simpson A, Talton L, Turner C, Tait A, Tivey A, Van Aken S, Walker D, Wanless D, Wang S, White B, White O, Whitehead S, Woodward J, Wortman J, Adams M, Embley T, Gull K, Ullu E, Barry J, Fairlamb A, Opperdoes F, Barret B, Donelson J, Hall N, Fraser C, Melville S, El-Sayed N. 2005. The genome of the African trypanosome *Trypanosoma brucei*. *Science* 309: 416-422.

Bettarel Y, Bouvy M, Dumont C, Sime-Ngando T. 2006. Virus-bacterium interactions in water and sediment of West African inland aquatic systems. Applied and Environmental Microbiology 72: 5274-5282.

Biderre C, Mathis A, Deplazes P, Weber R, Metenier G, Vivares C. 1999. Molecular karyotype diversity in the microsporidian *Encephalitozoon cuniculi*. *Parasitology* 118: 439-445.

Bi K, Bogart JP. 2010. Time and time again: unisexual salamanders (genus *Ambystoma*) are the oldest unisexual vertebrates. *Bmc Evolutionary Biology* 10.

Birky C. 2010. Positively negative evidence for asexuality. *Journal of Heredity* 101: S42-S45.

Birky C, Wolf C, Maughan H, Herbertson L, Henry E. 2005. Speciation and selection without sex. *Hydrobiologia* 546: 29-45.

Blackman R, De Boise E, Czylok A. 2001. Occurrence of sexual morphs in *Trama troglodytes* von Heyden, 1837 (Hemiptera, Aphididae). *Journal of Natural History* 35: 779-785.

Blackman R, Spence JM, Normark B. 2000. High diversity of structurally heterozygous karyotypes and rDNA arrays in parthenogenetic aphids of the genus *Trama* (Aphididae: Lachninae). *Heredity* 84: 254-260.

Blanc D, Nicholls R, Sargeaunt P. 1989. Experimental production of new zymodemes of *Entamoeba histolytica* supports the hypothesis of genetic exchange. *Transactions of the Royal Society of Tropical Medicine and Hygiene* 83: 787-790.

Blanc G, Duncan G, Agarkova I, Borodovsky M, Gurnon J, Kuo A, Lindquist E, Lucas S, Pangilinan J, Polle J, Salamov A, Terry A, Yamada T, Dunigan D, Grigoriev I, Claverie J, Van Etten J. 2010. The *Chlorella variabilis* NC64A genome reveals adaptation to photosymbiosis, coevolution with viruses, and cryptic sex. *Plant Cell* 22: 2943-2955.

Bloszyk J, Adamski Z, Napierala A, Dylewska M. 2004. Parthenogenesis as a life strategy among mites of the suborder Uropodina (Acari: Mesostigmata). *Canadian Journal of Zoology-Revue Canadienne De Zoologie* 82: 1503-1511.

Bloszyk J, Szymkowiak P. 1996. *Trachytes kaliszewskii*, n. sp. (Acari: Uropodina), from the Great Basin (Utah, USA), with remarks on the habitats and distribution of the members of the genus *Trachytes*. *Great Basin Naturalist* 56: 59-72.

Bochkov A, Walter D. 2007. The life-cycle of *Pomerantzia philippina* sp. n. (Prostigmata: Pomerantziidae) described from the Philippines. *Acarina* 15: 159-170.

Bode S, Adolfsson S, Lamatsch D, Martins M, Schmit O, Vandekerkhove J, Mezquita F, Namiotko T, Rossetti G, Schön I, Butlin R, Martens K. 2010. Exceptional cryptic diversity and multiple origins of parthenogenesis in a freshwater ostracod. *Molecular Phylogenetics and Evolution* 54: 542-552.

Bogart JP, Bi K, Fu JZ, Noble DWA, Niedzwiecki J. 2007. Unisexual salamanders (genus *Ambystoma*) present a new reproductive mode for eukaryotes. *Genome* 50: 119-136.

Boschetti C, Pouchkina-Stantcheva N, Hoffmann P, Tunnacliffe A. 2011. Foreign genes and novel hydrophilic protein genes participate in the desiccation response of the bdelloid rotifer *Adineta ricciae*. *Journal of Experimental Biology* 214: 59-68.

Bratbak G, Egge JK, Heldal M. 1993. Viral mortality of the marine alga *Emiliania huxleyi* (Haptophyceae) and termination of algal blooms. Marine Ecology Progress Series 93: 39-48.

Brues C. 1932. Further studies on the fauna of North American hot springs. *Proceedings of the American Academy of Arts and Sciences* 67: 185-303.

Bruvo R, Adolfsson S, Symonova R, Lamatsch D, Schön I, Jokela J, Butlin R, Muller S. 2011. Few parasites, and no evidence for *Wolbachia* infections, in a freshwater ostracod inhabiting temporary ponds. *Biological Journal of the Linnean Society* 102: 208-216.

Buckwold VE. 1994. Viral-induced extinctions unlikely. *Nature* 368: 562.

Bunbury J, Gajewski K. 2009. Biogeography of freshwater ostracodes in the Canadian arctic archipelago. *Arctic* 62: 324-332.

Butlin R, Schön I, Martens K. 1998. Asexual reproduction in nonmarine ostracods. *Heredity* 81: 473-480.

Caceres CE, Soluk DA. 2002. Blowing in the wind: a field test of overland dispersal and colonization by aquatic invertebrates. *Oecologia* 131: 402-408.

Caponetti J, Whitten M, Beck M. 1982. Axenic culture and induction of callus and sporophytes of the Appalachian *Vittaria* gametophyte. *American Fern Journal* 72: 36-40.

Carbonel P, Colin J, Danielopol D, Loffler H, Neustrueva I. 1988. Paleoecology of limnic ostracodes: a review of some major topics. *Palaeogeography Palaeoclimatology Palaeoecology* 62: 413-461.

Carlton J, Hirt R, Silva J, Delcher A, Schatz M, Zhao Q, Wortman J, Bidwell S, Alsmark U, Besteiro S, Sicheritz-Ponten T, Noel C, Dacks J, Foster P, Simillion C, Van de Peer Y, Miranda-Saavedra D, Barton G, Westrop G, Muller S, Dessi D, Fiori P, Ren Q, Paulsen I, Zhang H, Bastida-Corcuera F, Simoes-Barbosa A, Brown M, Hayes R, Mukherjee M, Okumura C, Schneider R, Smith A, Vanacova S, Villalvazo M, Haas B, Pertea M, Feldblyum T, Utterback T, Shu C, Osoegawa K, de Jong P, Hrdy I, Horvathova L, Zubacova Z, Dolezal P, Malik S, Logsdon J, Henze K, Gupta A, Wang C, Dunne R, Upcroft J, Upcroft P,

White O, Salzberg S, Tang P, Chiu C, Lee Y, Embley T, Coombs G, Mottram J, Tachezy J, Fraser-Liggett C, Johnson P. 2007. Draft genome sequence of the sexually transmitted pathogen *Trichomonas vaginalis*. *Science* 315: 207-212.

Carr M, Leadbeater B, Baldauf S. 2010. Conserved Meiotic Genes Point to Sex in the Choanoflagellates. *Journal of Eukaryotic Microbiology* 57: 56-62.

Carr M, Nelson M, Leadbeater B, Baldauf S. 2008. Three families of LTR retrotransposons are present in the genome of the choanoflagellate *Monosiga brevicollis*. *Protist* 159: 579-590.

Castagnonesereno P, Piotte C, Uijthof J, Abad P, Wajnberg E, Vanlerberghemasutti F, Bongiovanni M, Dalmasso A. 1993. Phylogenetic relationships between amphimictic and parthenogenetic nematodes of the genus *Meloidogyne* as inferred from repetitive DNA analysis. *Heredity* 70: 195-204.

Chapela I, Rehner S, Schultz T, Mueller U. 1994. Evolutionary history of the symbiosis between fungus-growing ants and their fungi. *Science* 266: 1691-1694.

Chaplin J, Hebert P. 1997. *Cyprinotus incongruens* (Ostracoda): an ancient asexual? *Molecular Ecology* 6: 155-168.

Chi J, Parrow M, Dunthorn M. 2014. Cryptic Sex in *Symbiodinium* (Alveolata, Dinoflagellata) is Supported by an Inventory of Meiotic Genes. *Journal of Eukaryotic Microbiology* 61: 322-327.

Cianciolo JM. 2009. Asexual species of oribatid mites do not have a local-scale colonization advantage over sexual species. *Evolutionary Ecology Research* 11: 43-55.

Cianciolo JM, Norton RA. 2006. The ecological distribution of reproductive mode in oribatid mites, as related to biological complexity. *Experimental and Applied Acarology* 40: 1-25.

Coleman DA, Crossley DA, Hendrix PF. 2004. *Fundamentals of Soil Ecology*, *Second edition*. USA: Elsevier Academic Press.

Cooper M, Adam R, Worobey M, Sterling C. 2007. Population genetics provides evidence for recombination in *Giardia*. *Current Biology* 17: 1984-1988.

Corradi N, Lildhar L. 2012. Meiotic genes in the arbuscular mycorrhizal fungi: What for? *Communicative & Integrative Biology* 5: 187-189.

Crane E. 1997. A revised circumscription of the genera of the fern family Vittariaceae. *Systematic Botany* 22: 509-517.

Crespi B, Sandoval C. 2000. Phylogenetic evidence for the evolution of ecological specialization in *Timema* walking-sticks. *Journal of Evolutionary Biology* 13: 249-262.

Crisp D, Standen A. 1988. *Lasaea rubra* (Montagu) (Bivalvia: Erycinacea), an apomictic crevice-living bivalve with clones separated by tidal level preference. *Journal of Experimental Marine Biology and Ecology* 117: 27-45.

Croll D, Sanders I. 2009. Recombination in *Glomus intraradices*, a supposed ancient asexual arbuscular mycorrhizal fungus. *Bmc Evolutionary Biology* 9.

Cuomo C, Desjardins C, Bakowski M, Goldberg J, Ma A, Becnel J, Didier E, Fan L, Heiman D, Levin J, Young S, Zeng Q, Troemel E. 2012. Microsporidian genome analysis reveals evolutionary strategies for obligate intracellular growth. *Genome Research* 22: 2478-2488.

Dabert M, Witalinski W, Kazmierski A, Olszanowski Z, Dabert J. 2010. Molecular phylogeny of acariform mites (Acari, Arachnida): strong conflict between phylogenetic signal and long-branch attraction artifacts. *Molecular Phylogenetics and Evolution* 56: 222-241.

Dacks J, Roger A. 1999. The first sexual lineage and the relevance of facultative sex. *Journal of Molecular Evolution* 48: 779-783.

Danovaro R, Dell'Anno A, Corinaldesi C, Magagnini M, Noble R, Tamburini C, Weinbauer M. 2008. Major viral impact on the functioning of benthic deep-sea ecosystems. *Nature* 454: 1084-1087.

Darby B, Neher D, Housman D, Belnap J. 2011. Few apparent short-term effects of elevated soil temperature and increased frequency of summer precipitation on the abundance and taxonomic diversity of desert soil micro- and meso-fauna. *Soil Biology & Biochemistry* 43: 1474-1481.

Dartnall H. 1983. Rotifers of the Antarctic and Subantarctic. *Hydrobiologia* 104: 57-60.

Dawkins R, Krebs JR. 1979. Arms races between and within species. *Proceedings of the Royal Society B-Biological Sciences* 205: 489-511.

Debortoli N, Li X, Eyres I, Fontaneto D, Hespeels B, Tang CQ, Flot J, Van Doninck K. 2016. Genetic exchange among bdelloid rotifers is more likely due to horizontal gene transfer than to meiotic sex. *Current Biology* 26: 723-732.

de Castro F, Bolker B. 2005. Mechanisms of disease-induced extinction. *Ecology Letters* 8: 117-126.

Delmotte F, Leterme N, Bonhomme J, Rispe C, Simon JC. 2001. Multiple routes to asexuality in an aphid species. *Proceedings of the Royal Society B-Biological Sciences* 268: 2291-2299.

Delmotte F, Sabater-Muñoz B, Prunier-Leterme N, Latorre A, Sunnucks P, Rispe C, Simon JC. 2003. Phylogenetic evidence for hybrid origins of asexual lineages in an aphid species. *Evolution* 57: 1291-1303.

Delorme L, Donald D. 1969. Torpidity of freshwater ostracodes. *Canadian Journal of Zoology* 47: 997-999.

De Smet WH. 2002. Marine rotifera from the Crozet and Kerguelen Islands (Subantarctica), with the description of a new *Encentrum* (Monogononta: Dicranophoridae). *International Review of Hydrobiology* 87: 411-422.

Devetter M, Scholl K. 2014. Hydrobiont animals in floodplain soil: are they positively or negatively affected by flooding? *Soil Biology & Biochemistry* 69: 393-397.

Docking TR, Saade FE, Elliott MC, Schoen DJ. 2006. Retrotransposon sequence variation in four asexual plant species. *Journal of Molecular Evolution* 62: 375-387.

Doerder F. 2014. Abandoning sex: multiple origins of asexuality in the ciliate *Tetrahymena*. *Bmc Evolutionary Biology* 14.

Doherty K, Zweifel E, Elde N, McKone M, Zweifel S. 2003. Random amplified polymorphic DNA markers reveal genetic variation in the symbiotic fungus of leaf-cutting ants. *Mycologia* 95: 19-23.

Domes K, Althammer M, Norton RA, Scheu S, Maraun M. 2007a. The phylogenetic relationship between Astigmata and Oribatida (Acari) as indicated by molecular markers. *Experimental and Applied Acarology* 42: 159-171.

Domes K, Norton R, Maraun M, Scheu S. 2007b. Reevolution of sexuality breaks Dollo's law. *Proceedings of the National Academy of Sciences of the United States of America* 104: 7139-7144.

Domes K, Scheu S, Maraun M. 2007c. Resources and sex: soil re-colonization by sexual and parthenogenetic oribatid mites. *Pedobiologia* 51: 1-11.

Donner J. 1975. Randbiotope von Fliessgewässern als orte der Anpassung von Wasserorganismen an Bodenbedingungen, gezeigt an Rotatorien der Donau und Nebenflüsse. In: Müller P, ed. *Verhandlungen der Gesellschaft für Ökologie Wien 1975*. Dordrecht: Springer Science+Business Media B.V., 231-234.

Drake LA, Choi KH, Haskell AE, Dobbs FC. 1998. Vertical profiles of virus-like particles and bacteria in the water column and sediments of Chesapeake Bay, USA. *Aquatic Microbial Ecology* 16: 17-25.

Drmota T, Kral J. 1997. Karyotype of *Trichomonas vaginalis*. *European Journal of Protistology* 33: 131-135.

Ebihara A, Farrar D, Ito M. 2008. The sporophyte-less filmy fern of eastern North America *Trichomanes intricatum* (Hymenophyllaceae) has the chloroplast genome of an Asian species. *American Journal of Botany* 95: 1645-1651.

Ebihara A, Matsumoto S, Ito M. 2009. Hybridization involving independent gametophytes in the *Vandenboschia radicans* complex (Hymenophyllaceae): a new perspective on the distribution of fern hybrids. *Molecular Ecology* 18: 4904-4911.

Edgecombe GD, GiribetG, Dunn CW, Hejnol A, Kristensen RM, Neves RC, Rouse GW, Worsaae K, Sørensen MV. 2011. Higher-level metazoan relationships: recent progress and remaining questions. *Organisms Diversity & Evolution* 11: 151-172.

Elliott ET, Anderson RV, Coleman DC, Cole CV. 1980. Habitable pore space and microbial trophic interactions. *Oikos* 35: 327-335.

El-Sayed N, Myler P, Bartholomeu D, Nilsson D, Aggarwal G, Tran A, Ghedin E, Worthey E, Delcher A, Blandin G, Westenberger S, Caler E, Cerqueira G, Branche C, Haas B, Anupama A, Arner E, Aslund L, Attipoe P, Bontempi E, Bringaud F, Burton P, Cadag E, Campbell D, Carrington M, Crabtree J, Darban H, da Silveira J, de Jong P, Edwards K, Englund P, Fazelina G, Feldblyum T, Ferella M, Frasch A, Gull K, Horn D, Hou L, Huang Y, Kindlund E, Ktingbeil M, Kluge S, Koo H, Lacerda D,

Levin M, Lorenzi H, Louie T, Machado C, McCulloch R, McKenna A, Mizuno Y, Mottram J, Nelson S, Ochaya S, Osoegawa K, Pai G, Parsons M, Pentony M, Pettersson U, Pop M, Ramirez J, Rinta J, Robertson L, Salzberg S, Sanchez D, Seyler A, Sharma R, Shetty J, Simpson A, Sisk E, Tammi M, Tarteton R, Teixeira S, Van Aken S, Vogt C, Ward P, Wickstead B, Wortman J, White O, Fraser C, Stuart K, Andersson B. 2005. The genome sequence of *Trypanosoma cruzi*, etiologic agent of Chagas disease. *Science* 309: 409-415.

Elzinga J, Jokela J, Shama L. 2013. Large variation in mitochondrial DNA of sexual and parthenogenetic *Dahlica triquetrella* (Lepidoptera: Psychidae) shows multiple origins of parthenogenesis. *Bmc Evolutionary Biology* 13.

Emiliani C. 1982. Extinctive evolution: extinctive and competitive evolution combine into a unified model of evolution. *Journal of Teoretical Biology* 97: 13-33.

Emiliani C. 1993a. Extinction and viruses. *Biosystems* 31: 155-159.

Emiliani C. 1993b. Viral extinctions in deep-sea species. *Nature* 366: 217-218.

Etter RJ, Rex MA, Chase MR, Quattro JM. 2005. Population differentiation decreases with depth in deep-sea bivalves. *Evolution* 59: 1479-1491.

Farrar D. 1967. Gematophytes of four tropical fern genera reproducing independently of their sporophytes in the southern Appalachians. *Science* 155: 1266-1267.

Farrar D. 1974. Gemmiferous fern gametophytes–Vittariaceae. *American Journal of Botany* 61: 146-155.

Farrar D. 1978. Problems in the identity and origin of the Appalachian *Vittaria* gametophyte, a sporophyteless fern of the eastern United States. *American Journal of Botany* 65: 1-12.

Farrar D. 1985. Independent fern gametophytes in the wild. *Proceedings of the Royal Society of Edinburgh Section B-Biological Sciences* 86: 361-369.

Farrar D. 1990. Species and evolution in asexually reproducing independent fern gametophytes. *Systematic Botany* 15: 98-111.

Farrar D. 1992. *Trichomanes intricatum*: the independent *Trichomanes* gametophyte in the eastern United States. *American Fern Journal* 82: 68-74.

Farrar D. 1998. The tropical flora of rockhouse cliff formations in the eastern United States. *Journal of the Torrey Botanical Society* 125: 91-108.

Farrar D, Mickel J. 1991. *Vittaria appalachiana*: a name for the "Appalachian gametophyte". *American Fern Journal* 81: 69-75.

Farrell BD, Sequeira AS, O'Meara BC, Normark B, Chung JH, Jordal BH. 2001. The evolution of agriculture in beetles (Curculionidae: Scolytinae and Platypodinae). *Evolution* 55: 2011-2027.

Filippini M, Buesing N, Bettarel Y, Sime-Ngando T, Gessner MO. 2006. Infection paradox: high abundance but low impact of freshwater benthic viruses. *Applied and Environmental Microbiology* 72: 4893-4898.

Fisher UR, Wieltschnig C, Kirschner AKT, Velimirov B. 2003. Does virus-induced lysis contribute significantly to bacterial mortality in the oxygenated sediment layer of shallow oxbow lakes? *Applied and Environmental Microbiology* 69: 5281-5289.

Flot J, Debortoli N, Hallet B, Van Doninck K. 2016. Response to Signorovitch *et al. Current Biology* 26: R755.

Flot J, Hespeels B, Li X, Noel B, Arkhipova I, Danchin E, Hejnol A, Henrissat B, Koszul R, Aury J, Barbe V, Barthelemy R, Bast J, Bazykin G, Chabrol O, Couloux A, Da Rocha M, Da Silva C, Gladyshev E, Gouret P, Hallatschek O, Hecox-Lea B, Labadie K, Lejeune B, Piskurek O, Poulain J, Rodriguez F, Ryan J, Vakhrusheva O, Wajnberg E, Wirth B, Yushenova I, Kellis M, Kondrashov A, Welch D, Pontarotti P, Weissenbach J, Wincker P, Jaillon O, Van Doninck K. 2013. Genomic evidence for ameiotic evolution in the bdelloid rotifer *Adineta vaga*. *Nature* 500: 453-457.

Fontaneto D, Jondelius U. 2011. Broad taxonomic sampling of mitochondrial cytochrome c oxidase subunit I does not solve the relationships between Rotifera and Acanthocephala. *Zoologischer Anzeiger* 250: 80-85.

Forche A, Alby K, Schaefer D, Johnson A, Berman J, Bennett R. 2008. The parasexual cycle in *Candida albicans* provides an alternative pathway to meiosis for the formation of recombinant strains. *Plos Biology* 6: 1084-1097.

Frisch D, Green A, Figuerola J. 2007. High dispersal capacity of a broad spectrum of aquatic invertebrates via waterbirds. *Aquatic Sciences* 69: 568-574.

Fritz-Laylin L, Prochnik S, Ginger M, Dacks J, Carpenter M, Field M, Kuo A, Paredez A, Chapman J, Pham J, Shu S, Neupane R, Cipriano M, Mancuso J, Tu H, Salamov A, Lindquist E, Shapiro H, Lucas S, Grigoriev I, Cande W, Fulton C, Rokhsar D, Dawson S. 2010. The Genome of *Naegleria gruberi* Illuminates Early Eukaryotic Versatility. *Cell* 140: 631-642.

Fucikova K, Pazoulova M, Rindi F. 2015. Meiotic genes and sexual reproduction in the green algal class Trebouxiophyceae (chlorophyta). *Journal of Phycology* 51: 419-430.

Fuhrman JA. 1999. Marine viruses and their biogeochemical and ecological effects. *Nature* 399: 541-548

Gandolfi A, Bonilauri P, Rossi V, Menozzi P. 2001. Intraindividual and intraspecies variability of ITS1 sequences in the ancient asexual *Darwinula stevensoni* (Crustacea: Ostracoda). *Heredity* 87: 449-455.

Gandolfi A, Sanders I, Rossi V, Menozzi P. 2003. Evidence of recombination in putative ancient asexuals. *Molecular Biology and Evolution* 20: 754-761.

Garcia-Varela M, Nadler SA. 2006. Phylogenetic relationships among Syndermata inferred from nuclear and mitochondrial gene sequences. *Molecular Phylogenetics and Evolution* 40: 61-72.

Garey J, Near T, Nonnemacher M, Nadler S. 1996. Molecular evidence for Acanthocephala as a subtaxon of Rotifera. *Journal of Molecular Evolution* 43: 287-292.

Gastony G. 1977. Chromosomes of the independently reproducing Appalachian gametophyte: a new source of taxonomic evidence. *Systematic Botany* 2: 43-48.

Gaunt M, Yeo M, Frame I, Stothard J, Carrasco H, Taylor M, Mena S, Veazey P, Miles G, Acosta N, de Arias A, Miles M. 2003. Mechanism of genetic exchange in American trypanosomes. *Nature* 421: 936-939.

Giovannetti M, Sbrana C, Strani P, Agnolucci M, Rinaudo V, Avio L. 2003. Genetic diversity of isolates of *Glomus mosseae* from different geographic areas detected by vegetative compatibility testing and biochemical and molecular analysis. *Applied and Environmental Microbiology* 69: 616-624.

Gladyshev E, Arkhipova I. 2007. Telomere-associated endonuclease-deficient Penelope-like retroelements in diverse eukaryotes. *Proceedings of the National Academy of Sciences of the United States of America* 104: 9352-9357.

Gladyshev E, Meselson M. 2008. Extreme resistance of bdelloid rotifers to ionizing radiation. *Proceedings of the National Academy of Sciences of the United States of America* 105: 5139-5144.

Gladyshev E, Meselson M, Arkhipova I. 2007. A deep-branching clade of retrovirus-like transposons in bdelloid rotifers. *Gene* 390: 136-149.

Gladyshev E, Meselson M, Arkhipova I. 2008. Massive horizontal gene transfer in bdelloid rotifers. *Science* 320: 1210-1213.

Glöckner G, Hulsmann N, Schleicher M, Noegel A, Eichinger L, Gallinger C, Pawlowski J, Sierra R, Euteneuer U, Pillet L, Moustafa A, Platzer M, Groth M, Szafranski K, Schliwa M. 2014. The Genome of the Foraminiferan *Reticulomyxa filosa*. *Current Biology* 24: 11-18.

Gomez-Zurita J, Funk D, Vogler A. 2006. The evolution of unisexuality in *Calligrapha* leaf beetles: molecular and ecological insights on multiple origins via interspecific hybridization. *Evolution* 60: 328-347.

Goodwin T, Poulter R. 2000. Multiple LTR-retrotransposon families in the asexual yeast *Candida albicans*. *Genome Research* 10: 174-191.

Grapputo A, Kumpulainen T, Mappes J. 2005. Phylogeny and evolution of parthenogenesis in Finnish bagworm moth species (Lepidoptera: Psychidae: Naryciinae) based on mtDNA-markers. *Annales Zoologici Fennici* 42: 141-160.

Graser Y, Volovsek M, Arrington J, Schonian G, Presber W, Mitchell T, Vilgalys R. 1996. Molecular markers reveal that population structure of the human pathogen *Candida albicans* exhibits both clonality and recombination. *Proceedings of the National Academy of Sciences of the United States of America* 93: 12473-12477.

Grigg M, Bonnefoy S, Hehl A, Suzuki Y, Boothroyd J. 2001. Success and virulence in *Toxoplasma* as the result of sexual recombination between two distinct ancestries. *Science* 294: 161-165.

Grimsley N, Pequin B, Bachy C, Moreau H, Piganeau G. 2010. Cryptic sex in the smallest eukaryotic marine green alga. *Molecular Biology and Evolution* 27: 47-54.

Gwiazdowicz D, Klemt J. 2004. Mesostigmatic mites (Acari, Gamasida) in selected microhabitats of the Biebrza National Park (NE Poland). *Biological Letters* 41.

Haag K, Sheikh-Jabbari E, Ben-Ami F, Ebert D. 2013. Microsatellite and single-nucleotide polymorphisms indicate recurrent transitions to asexuality in a microsporidian parasite. *Journal of Evolutionary Biology* 26: 1117-1128.

Halary S, Malik S-B, Lildhar L, Slamovits C, Hijri M, Corradi N. 2011. Conserved meiotic machinery in *Glomus* spp., a putatively ancient asexual fungal lineage. *Genome Biology and Evolution* 3: 950-958.

Halkett F, Plantegenest M, Prunier-Leterme N, Mieuzet L, Delmotte F, Simon J. 2005. Admixed sexual and facultatively asexual aphid lineages at mating sites. *Molecular Ecology* 14: 325-336.

Hammer M, Wallwork J. 1979. A review of the world distribution of oribatid mites (Acari: Cryptostigmata) in relation to continental drift. *Biologiske Skrifter Kongelige Danske Videnskabernes Selskab* 22: 5-31.

Hampl V, Pavlicek A, Flegr J. 2001. Construction and bootstrap analysis of DNA fingerprinting-based phylogenetic trees with the freeware program FreeTree: application to trichomonad parasites. *International Journal of Systematic and Evolutionary Microbiology* 51: 731-735.

Havill N, Foottit R, von Dohlen C. 2007. Evolution of host specialization in the Adelgidae (Insecta: Hemiptera) inferred from molecular phylogenetics. *Molecular Phylogenetics and Evolution* 44: 357-370.

Hebert P, Remigio E, Colbourne J, Taylor D, Wilson C. 2002. Accelerated molecular evolution in halophilic crustaceans. *Evolution* 56: 909-926.

Hedges SB, Bogart JP, Maxson LR. 1992. Ancestry of unisexual salamanders. *Nature* 356: 708-710.

Heethoff M. 2004. Genetic diversity and evolutionary age of parthenogenetic oribatid mites (Acari: Oribatida). Unpublished D. Phil. Thesis, Technischen Universität Darmstadt.

Heethoff M, Domes K, Laumann M, Maraun M, Norton RA, Scheu S. 2007. High genetic divergences indicate ancient separation of parthenogenetic lineages of the oribatid mite *Platynothrus peltifer* (Acari, Oribatida). *Journal of Evolutionary Biology* 20: 392-402.

Heethoff M, Norton R, Scheu S, Maraun M. 2009. Parthenogenesis in oribatid mites (Acari, Oribatida): evolution without sex. In: Schön I, Martens K, van Dijk P, eds. *Lost Sex: The Evolutionary Biology of Parthenogenesis*. Dordrecht: Springer Science+Business Media B.V., 241-257.

Heitman J. 2006. Sexual reproduction and the evolution of microbial pathogens. *Current Biology* 16: R711-R725.

Heller J, Farstey V. 1990. Sexual and parthenogenetic populations of the freshwater snail *Melanoides tuberculata* in Israel. *Israel Journal of Ecology* 37: 75-87.

Henry L, Schwander T, Crespi B. 2012. Deleterious mutation accumulation in asexual *Timema* stick insects. *Molecular Biology and Evolution* 29: 401-408.

Herlyn H, Piskurek O, Schmitz J, Ehlers U, Zischler H. 2003. The syndermatan phylogeny and the evolution of acanthocephalan endoparasitism as inferred from 18S rDNA sequences. *Molecular Phylogenetics and Evolution* 26: 155-164.

Hill D, Wetherbee R. 1986. Proteomonas sulcata gen. et sp. nov. (Cryptophyceae), a cryptomonad with two morphologically distinct and alternating forms. *Phycologia* 25: 521-543.

Hinkle G, Wetterer J, Schultz T, Sogin M. 1994. Phylogeny of the attine ant fungi based on analysis of small subunit ribosomal RNA gene sequences. *Science* 266: 1695-1697.

Horne D, Baltanás A, Paris G. 1998. Geographical distribution of reproductive modes. In: Martens K, ed. Sex and Parthenogenesis: Evolutionary Ecology of *Reproductive Modes in Non-Marine Ostracds*. Leiden: Backhuys. 77-99.

Horne F. 1993. Survival strategy to escape desiccation in a freshwater ostracod. *Crustaceana* 65: 53-61.

Howe D, Sibley L. 1995. *Toxoplasma gondii* comprises three clonal lineages: correlation of parasite genotype with human disease. *Journal of Infectious Diseases* 172: 1561-1566.

Hörandl E. 2006. The complex causality of geographical parthenogenesis. *New Phytologist* 171: 525-538.

Hörandl E. 2009. Geographical parthenogenesis: opportunities for asexuality. In: Schön I, Martens K, van Dijk P, eds. *Lost Sex: The Evolutionary Biology of Parthenogenesis*. Dordrecht: Springer Science+Business Media B.V., 161-186.

Hsu W. 1956a. Oogenesis in *Habrotrocha tridens* (Milne). *Biological bulletin* 111: 364-374.

Hsu W. 1956b. Oogenesis in the Bdelloidea rotifer *Philodina roseola* Ehrenberg. *Cellule* 57: 283-296.

Hull C, Johnson A. 1999. Identification of a mating type-like locus in the asexual pathogenic yeast *Candida albicans*. *Science* 285: 1271-1275.

Hur JH, Van Doninck K, Mandigo ML, Meselson M. 2009. Degenerate tetraploidy was established before bdelloid rotifer families diverged. *Molecular Biology and Evolution* 26: 375-383.

Ironside J. 2007. Multiple losses of sex within a single genus of microsporidia. *Bmc Evolutionary Biology* 7.

Issel R. 1900. Saggio sulla fauna termale italiana, Nota I. *Atti della Reale Accademia delle scienze di Torino* 36: 53-73.

Issel R. 1901. Saggio sulla fauna termale italiana, Nota II. *Atti della Reale Accademia delle scienze di Torino* 36: 265-277.

Jana B, Sarkar H. 1971. The limnology of “Swetganga”—A thermal spring of Bakreswar, West Bengal, India. *Hydrobiologia* 37: 33-47.

Janiec K. 1996. The comparison of freshwater invertebrates of Spitsbergen (Arctic) and King George Island (Antarctic). *Polish Polar Research* 17: 173-202.

Johnson S. 2006. Geographic ranges, population structure, and ages of sexual and parthenogenetic snail lineages. *Evolution* 60: 1417-1426.

Johnson S, Bragg E. 1999. Age and polyphyletic origins of hybrid and spontaneous parthenogenetic *Campeloma* (Gastropoda: Viviparidae) from the southeastern United States. *Evolution* 53: 1769-1781.

Jorgensen A, Mobjerg N, Kristensen R. 2007. Molecular study of the tardigrade *Echiniscus testudo* (Echiniscidae) reveals low DNA sequence diversity over a large geographical area. *Journal of Limnology* 66: 77-83.

Judson OP, Normark B. 1996. Ancient asexual scandals. *Trends in Ecology & Evolution* 11: A41-A46.

Jungblut AD, Vincent WF, Lovejoy C. 2012. Eukaryotes in Arctic and Antarctic cyanobacterial mats. *Fems Microbiology Ecology* 82: 416-428.

Karasawa S, Hijii N. 2008. Vertical stratification of oribatid (Acari: Oribatida) communities in relation to their morphological and life-history traits and tree structures in a subtropical forest in Japan. *Ecological Research* 23: 57-69.

Kessenich C, Ruck E, Schurko A, Wickett N, Alverson A. 2014. Transcriptomic insights into the life history of Bolidophytes, the sister lineage to diatoms. *Journal of Phycology* 50: 977-983.

Kethley J. 1989. Occurrence of *Pomerantzia kethleyi* (Acari: Prostigmata: Pomerantziidae) in Illinois and Minnesota. *Great Lakes Entomologist* 22: 101-101.

Khan A, Fux B, Su C, Dubey J, Darde M, Ajioka J, Rosenthal B, Sibley L. 2007. Recent transcontinental sweeps of *Toxoplasma gondii* driven by a single monomorphic chromosome. *Proceedings of the National Academy of Sciences of the United States of America* 104: 14872-14877.

Klie V. 1939. Zur Kenntnis von *Cypris balnearia* Moniez (Ostracoda*). Zoologische Anzeiger* 126: 298-302.

Koste W, Shiel RJ. 1986. Rotifera from Australian inland waters. I. Bdelloidea (Rotifera: Digononta). *Australian Journal of Marine and Freshwater Research* 37: 765-792.

Krivolutsky D, Druk A. 1986. Fossil oribatid mites. *Annual Review of Entomology* 31: 533-545.

Kugrens P, Lee R. 1988. Ultrastructure of fertilization in a cryptomonad. *Journal of Phycology* 24: 385-393.

Külköylüoğlu O, Meisch C, Rust R. 2003. *Thermopsis thermophila* n. gen. n. sp. from hot springs in Nevada, USA (Crustacea, Ostracoda). *Hydrobiologia* 499: 113-123.

Külköylüoğlu O, Vinyard G. 2000. Distribution and ecology of freshwater Ostracoda (Crustacea) collected from springs of Nevada, Idaho, and Oregon: A preliminary study. *Western North American Naturalist* 60: 291-303.

Lanteri A, Marvaldi A. 1995. *Graphognathus* Buchanan a new synonym of *Naupactus* Dejean and systematics of the *N. Leucoloma* species group (Coleoptera: Curculionidae). *The Coleopterists Bulletin* 49: 206-228.

Lasek-Nesselquist E. 2012. A mitogenomic re-evaluation of the bdelloid phylogeny and relationships among the Syndermata. *Plos One* 7.

Laumann M, Bergmann P, Heethoff M. 2008. Some remarks on the cytogenetics of oribatid mites. *Soil Organisms* 80: 223-232.

Laumann M, Norton R, Weigmann G, Scheu S, Maraun M, Heethoff M. 2007. Speciation in the parthenogenetic oribatid mite genus Tectocepheus (Acari, Oribatida) as indicated by molecular phylogeny. *Pedobiologia* 51: 111-122.

Lavelle P, Spain AV. 2003. *Soil Ecology*. New York, USA: Kluwer Academic Publishers.

Law J, Crespi B. 2002a. Recent and ancient asexuality in *Timema* walkingsticks. *Evolution* 56: 1711-1717.

Law J, Crespi B. 2002b. The evolution of geographic parthenogenesis in *Timema* walking-sticks. *Molecular Ecology* 11: 1471-1489.

Levin T, King N. 2013. Evidence for Sex and Recombination in the Choanoflagellate *Salpingoeca rosetta*. *Current Biology* 23: 2176-2180.

Liebau A. 2005. A revised classification of the higher taxa of the Ostracoda (Crustacea). *Hydrobiologia* 538: 115-137.

Li J, Ó Foighil D, Park J. 2013. Triton's trident: cryptic Neogene divergences in a marine clam (*Lasaea australis*) correspond to Australia's three temperate biogeographic provinces. *Molecular Ecology* 22: 1933-1946.

Lunt D. 2008. Genetic tests of ancient asexuality in root knot nematodes reveal recent hybrid origins. *Bmc Evolutionary Biology* 8.

McLay C. 1978. Comparative observations on the ecology of four species of ostracods living in a temporary freshwater puddle. *Canadian Journal of Zoology* 56: 663-375.

MacLeod A, Tweedie A, McLellan S, Taylor S, Cooper A, Sweeney L, Turner C, Tait A. 2005. Allelic segregation and independent assortment in *T. brucei* crosses: Proof that the genetic system is Mendelian and involves meiosis. *Molecular and Biochemical Parasitology* 143: 12-19.

Maddocks R. 1976. Quest for the ancestral podocopid: numerical cladistic analysis of ostracode appendages, a preliminary report. *Abhandlungen und Verhandlungen des Naturwissenschaftlichen Vereins in Hamburg* 18: 39-53.

Malik S, Pightling A, Stefaniak L, Schurko A, Logsdon J. 2008. An Expanded Inventory of Conserved Meiotic Genes Provides Evidence for Sex in *Trichomonas vaginalis*. *Plos One* 3.

Malmqvist B, Meisch C, Nilsson A. 1997. Distribution patterns of freshwater Ostracoda (Crustacea) in the Canary Islands with regards to habitat use and biogeography. *Hydrobiologia* 347: 159-170.

Mantovani B, Passamonti M, Scali V. 2001. The mitochondrial cytochrome oxidase II gene in *Bacillus* stick insects: ancestry of hybrids, androgenesis, and phylogenetic relationships. *Molecular Phylogenetics and Evolution* 19: 157-163.

Maraun M, Erdmann G, Schulz G, Norton RA, Scheu S, Domes K. 2009. Multiple convergent evolution of arboreal life in oribatid mites indicates the primacy of ecology. *Proceedings of the Royal Society B-Biological Sciences* 276: 3219-3227.

Maraun M, Heethoff M, Scheu S, Norton RA, Weigmann G, Thomas RH. 2003. Radiation in sexual and parthenogenetic oribatid mites (Oribatida, Acari) as indicated by genetic divergence of closely related species. *Experimental and Applied Acarology* 29: 265-277.

Maraun M, Heethoff M, Schneider K, Scheu S, Weigmann G, Cianciolo J, Thomas RH, Norton RA. 2004. Molecular phylogeny of oribatid mites (Oribatida, Acari): evidence for multiple radiations of parthenogenetic lineages. *Experimental and Applied Acarology* 33: 183-201.

Maraun M, Norton RA, Ehnes RB, Scheu S, Erdmann G. 2012. Positive correlation between density and parthenogenetic reproduction in oribatid mites (Acari) supports the structured resource theory of sexual reproduction. *Evolutionary Ecology Research* 14: 311-323.

Marshall W, Berbee M. 2010. Population-level analyses indirectly reveal cryptic sex and life history traits of *Pseudoperkinsus tapetis* (Ichthyosporea, Opisthokonta): a unicellular relative of the animals. *Molecular Biology and Evolution* 27: 2014-2026.

Martens K. 1998. Sex and ostracods: a new synthesis. In: Martens K, ed. *Sex and Parthenogenesis: Evolutionary Ecology of Reproductive Modes in Non-Marine Ostracds*. Leiden: Backhuys, 295-321.

Martens K, Horne D, Griffiths H. 1998a. Age and diversity of non-marine ostracods. In: Martens K, ed. *Sex and Parthenogenesis: Evolutionary Ecology of Reproductive Modes in Non-Marine Ostracds*. Leiden: Backhuys, 37-55.

Martens K, Rossetti G, Baltanás A. 1998b. Reproductive modes and taxonomy. In: Martens K, ed. *Sex and Parthenogenesis: Evolutionary Ecology of Reproductive Modes in Non-Marine Ostracds*. Leiden: Backhuys, 197-214.

Martens K, Rossetti G, Butlin R, Schön I. 2005. Molecular and morphological phylogeny of the ancient asexual Darwinulidae (Crustacea, Ostracoda). *Hydrobiologia* 538: 153-165.

Martens K, Rossetti G, Fuhrmann R. 1997. Pleistocene and recent species of the family Darwinulidae Brady & Norman, 1889 (Crustacea, Ostracoda) in Europe. *Hydrobiologia* 357: 99-116.

Martens K, Rossetti G, Horne D. 2003. How ancient are ancient asexuals? *Proceedings of the Royal Society B-Biological Sciences* 270: 723-729.

Martens K, Savatenalinton S. 2011. A subjective checklist of the recent, free-living, non-marine Ostracoda (Crustacea). *Zootaxa*: 1-79.

Martens K, Schön I. 2000. The importance of habitat stability for the prevalence of sexual reproduction. In: Minoura K, ed. *Lake Baikal: a Mirror in Time and Space for Understanding Global Change Processes. Yokohama Symposium 1998*. Amsterdam: Elsevier, 324-330.

Martens K, Schön I, Meisch C, Horne D. 2008. Global diversity of ostracods (Ostracoda, Crustacea) in freshwater. *Hydrobiologia* 595: 185-193.

Mateos M, Vrijenhoek RC. 2002. Ancient versus reticulate origin of a hemiclonal lineage. *Evolution* 56: 985-992.

Maynard Smith J. 1986. Evolution: contemplating life without sex. *Nature* 324: 300-301.

Mayr E. 1963. *Animal species and Evolution*. Harvard University Press: Cambridge.

McDermott T, Skorupa D. 2011. *Microbiology of Serpentine Hot Springs, Alaska*. Montana State University: Bozeman.

McGrath D, Ó Foighil D. 1986. Population dynamics and reproduction of hermaphroditic *Lasaea rubra* (Montagu) (Bivalvia, Galeommatacea). *Ophelia* 25: 209-219.

McLay C. 1978. Comparative observations on the ecology of four species of ostracods living in a temporary freshwater puddle. *Canadian Journal of Zoology* 56: 663-375.

Melone G, Ricci C. 1995. Rotatory apparatus in Bdelloids. *Hydrobiologia* 313: 91-98.

Menzel R. 1923. Beitrage zur kenntnis der Mikrofauna von Niederiandisch-Ostidien V. Moosbewohnende Ostracoden aus dem Urwald von Tjibodas. *Treubia* 3: 193-196.

Mikheyev AS, Mueller UG, Abbot P. 2006. Cryptic sex and many-to-one colevolution in the fungus-growing ant symbiosis. *Proceedings of the National Academy of Sciences of the United States of America* 103: 10702-10706.

Miller W, Claxton S, Heatwole H. 1999. Tardigrades of the Australian Antarctic territories: Males in the genus Echiniscus (Tardigrada: Heterotardigrada). *Zoologischer Anzeiger* 238: 303-309.

Min GS, Park JK. 2009. Eurotatorian paraphyly: revisiting phylogenetic relationships based on the complete mitochondrial genome sequence of *Rotaria rotatoria* (Bdelloidea: Rotifera: Syndermata). *Bmc Genomics* 10.

Molostovskaya I. 2000. The evolutionary history of Late Permian Darwinulocopina Sohn, 1988 (Ostracoda) from the Russian Plate. *Hydrobiologia* 419: 125-130.

Moniez R. 1893. Description d'une nouvelle espèce de Cypris vivant dans les eaux thermales du Hammam-Meskhoutine. *Bulletin de la Société zoologique de France* 18: 140-142.

Montelongo T, Gomez-Zurita J. 2015. Nonrandom Patterns of Genetic Admixture Expose the Complex Historical Hybrid Origin of Unisexual Leaf Beetle Species in the Genus *Calligrapha*. *American Naturalist* 185: 113-134.

Moran N. 1992. The evolution of aphid life cycles. *Annual Review of Entomology* 37: 321-348.

Morris D. 2012. A new model for myxosporean (Myxozoa) development explains the endogenous budding phenomenon, the nature of cell within cell life stages and evolution of parasitism from a cnidarian ancestor. *International Journal For Parasitology* 42: 829-840.

Morrison H, McArthur A, Gillin F, Aley S, Adam R, Olsen G, Best A, Cande W, Chen F, Cipriano M, Davids B, Dawson S, Elmendorf H, Hehl A, Holder M, Huse S, Kim U, Lasek-Nesselquist E, Manning G, Nigam A, Nixon J, Palm D, Passamaneck N, Prabhu A, Reich C, Reiner D, Samuelson J, Svard S, Sogin M. 2007. Genomic minimalism in the early diverging intestinal parasite *Giardia lamblia*. *Science* 317: 1921-1926.

Morrison J. 1954. The relationships of old and new world melanians. *Proceedings of the United States National Museum* 103: 357-394.

Morton JE, Boney AD, Corner EDS. 1957. The adaptations of *Lasaea rubra* (Montagu), a small intertidal lamellibranch. *Journal of the Marine Biological Association of the United Kingdom* 36: 383-405.

Mueller U. 2002. Ant versus fungus versus mutualism: ant–cultivar conflict and the deconstruction of the attine ant–fungus symbiosis. *American Naturalist* 160: S67-S98.

Mueller U, Gerardo N, Aanen D, Six D, Schultz T. 2005. The evolution of agriculture in insects. *Annual Review of Ecology Evolution and Systematics* 36: 563-595.

Mueller U, Rehner SA, Schultz TR. 1998. The evolution of agriculture in ants. *Science* 281: 2034-2038.

Muñoz J, Gómez A, Green A, Figuerola J, Amat F, Rico C. 2010. Evolutionary origin and phylogeography of the diploid obligate parthenogen *Artemia parthenogenetica* (Branchiopoda: Anostraca). *Plos One* 5.

Murphy SL, Tate RL. 1996. Bacterial movement through soil. In: Stozsky G & Bollag JM, ed. *Soil Biochemistry Vol. 9.* New York: Marcel Dekker, 253-286.

Nascetti G, Bondanelli P, Aldinucci A, Cimmaruta R. 2003. Genetic structure of bisexual and parthenogenetic populations of *Artemia* from Italian brackish-hypersaline waters. *Oceanologica Acta* 26: 93-100.

Neher D, Lewins S, Weicht T, Darby B. 2009. Microarthropod communities associated with biological soil crusts in the Colorado Plateau and Chihuahuan deserts. *Journal of Arid Environments* 73: 672-677.

Neiman M, Jokela J, Lively C. 2005. Variation in asexual lineage age in *Potamopyrgus antipodarum*, a New Zealand snail. *Evolution* 59: 1945-1952.

Neiman M, Larkin K, Thompson A, Wilton P. 2012. Male offspring production by asexual *Potamopyrgus antipodarum*, a New Zealand snail. *Heredity* 109: 57-62.

Neiman M, Lively C. 2004. Pleistocene glaciation is implicated in the phylogeographical structure of *Potamopyrgus antipodarum*, a New Zealand snail. *Molecular Ecology* 13: 3085-3098.

Neiman M, Lively C. 2005. Male New Zealand mud snails (*Potamopyrgus antipodarum*) persist in copulating with asexual and parasitically castrated females. *American Midland Naturalist* 154: 88-96.

Normark B. 1996. Phylogeny and evolution of parthenogenetic weevils of the *Aramigus tessellatus* species complex (Coleoptera: Curculionidae: Naupactini): Evidence from mitochondrial DNA sequences. *Evolution* 50: 734-745.

Normark B. 1999. Evolution in a putatively ancient asexual aphid lineage: recombination and rapid karyotype change. *Evolution* 53: 1458-1469.

Normark B, Judson O, Moran N. 2003. Genomic signatures of ancient asexual lineages. *Biological Journal of the Linnean Society* 79: 69-84.

Normark B, Lanteri A. 1998. Incongruence between morphological and mitochondrial-DNA characters suggests hybrid origins of parthenogenetic weevil lineages (genus *Aramigus*). *Systematic Biology* 47: 475-494.

Normark B, Moran NA. 2000. Opinion – Testing for the accumulation of deleterious mutations in asexual eukaryote genomes using molecular sequences. *Journal of Natural History* 34: 1719-1729.

Norton R. 1994. Evolutionary aspects of oribatid mite life histories and consequences for the origin of the Astigmata. In: Houck M, ed. *Mites. Ecological and Evolutionary Analyses of Life-History Patterns*. New York: Chapman & Hall, 99-135.

Norton R, Behan-Pelletier V. 2009. Suborder Oribatida. In: Krantz G, Walter D, eds. *A manual of acarology*. Lubbock: Texas Tech University Press, 430-564.

Norton R, Kethley J, Johnston D, O'Connor B. 1993. Phylogenetic perspectives on genetic systems and reproductive modes of mites. In: Wrensch D, Ebbert M, eds. *Evolution and diversity of sex ratio in insects and mites*. New York: Chapman and Hall, 8-99.

Norton R, Palmer S. 1991. The distribution, mechanisms and evolutionary significance of parthenogenesis in oribatid mites. In: Schuster R, Murphy P, eds. *The Acari: Reproduction, Development and Life-history Strategies*. London: Chapman and Hall, 107-136.

Nowacki M, Shetty K, Landweber L, Chakravarti A, Green E. 2011. RNA-Mediated Epigenetic Programming of Genome Rearrangements. *Annual Review of Genomics and Human Genetics, Vol 12* 12: 367-389.

Nowacki M, Vijayan V, Zhou Y, Schotanus K, Doak T, Landweber L. 2008. RNA-mediated epigenetic programming of a genome-rearrangement pathway. *Nature* 451: 153-158.

Oconnor B. 2009. Cohort Astigmatina. In: Krantz G, Walter D, eds. *A manual of acarology*. Lubbock: Texas Tech University Press, 565-657.

Odds F, Bougnoux M, Shaw D, Bain J, Davidson A, Diogo D, Jacobsen M, Lecomte M, Li S, Tavanti A, Maiden M, Gow N, d'Enfert C. 2007. Molecular phylogenetics of *Candida albicans*. *Eukaryotic Cell* 6: 1041-1052.

O'Gorman C, Fuller H, Dyer P. 2009. Discovery of a sexual cycle in the opportunistic fungal pathogen *Aspergillus fumigatus*. *Nature* 457: 471-474.

Oliveira R, Broude N, Macedo A, Cantor C, Smith C, Pena S. 1998. Probing the genetic population structure of *Trypanosoma cruzi* with polymorphic microsatellites. *Proceedings of the National Academy of Sciences of the United States of America* 95: 3776-3780.

Ó Foighil D. 1985. Fine structure of *Lasaea subviridis* and *Mysella tumida* sperm (Bivalvia, Galeommatacea). *Zoomorphology* 105: 125-132.

Ó Foighil D. 1987. Cytological evidence for self-fertilization in *Lasaea subviridis* (Galeommatacea: Bivalvia). *International Journal of Invertebrate Reproduction and Development* 12: 83-89.

Ó Foighil D. 1988. Random mating and planktotrophic larval development in the brooding hermaphroditic clam *Lasaea australis* (Lamarck, 1818). *Veliger* 31: 214-221.

Ó Foighil D. 1989. Planktotrophic larval development is associated with a restricted geographic range in *Lasaea*, a genus of brooding, hermaphroditic bivalves. *Marine Biology* 103: 349-358.

Ó Foighil D, Eernisse D. 1988. Geographically widespread, non-hybridizing, sympatric strains of the hermaphroditic, brooding clam *Lasaea* in the northeastern Pacific Ocean. *Biological Bulletin* 175: 218-229.

Ó Foighil D, Jozefowicz C. 1999. Amphi-Atlantic phylogeography of direct-developing lineages of *Lasaea*, a genus of brooding bivalves. *Marine Biology* 135: 115-122.

Ó Foighil D, Smith M. 1995. Evolution of asexuality in the cosmopolitan marine clam *Lasaea*. *Evolution* 49: 140-150.

Ó Foighil D, Smith M. 1996. Phylogeography of an asexual marine clam complex, *Lasaea*, in the northeastern Pacific based on cytochrome oxidase III sequence variation. *Molecular Phylogenetics and Evolution* 6: 134-142.

Ó Foighil D, Thiriot-Quiévreux C. 1991. Ploidy and pronuclear interaction in northeastern Pacific *Lasaea* clones (Mollusca: Bivalvia). *Biological Bulletin* 181: 222-231.

Ó Foighil D, Thiriot-Quievreux C. 1999. Sympatric Australian *Lasaea* species (Mollusca: Bivalvia) differ in their ploidy levels, reproductive modes and developmental modes. *Zoological Journal of the Linnean Society* 127: 477-494.

Palmer S, Norton R. 1990. Further experimental proof of thelytokous parthenogenesis in oribatid mites (Acari: Oribatida: Desmonomata). *Experimental and Applied Acarology* 8: 149-159.

Palmer S, Norton R. 1991. Taxonomic, geographic and seasonal distribution of thelytokous parthenogenesis in the Desmonomata (Acari: Oribatida). *Experimental & Applied Acarology* 12: 67-81.

Palmer S, Norton R. 1992. Genetic diversity in thelytokous oribatid mites (Acari; Acariformes: Desmonomata). *Biochemical Systematics and Ecology* 20: 219-231.

Pan K, Qin J, Li S, Dai W, Zhu B, Jin Y, Yu W, Yang G, Li D. 2011. Nuclear monoploidy and asexual propagation of *Nannochloropsis oceanica* (eustigmatophyceae) as revealed by its genome sequence. *Journal of Phycology* 47: 1425-1432.

Parks J, Farrar D. 1984. A first report of the fern genus *Vittaria* in New York. *Rhodora* 86.

Paul EA. 2007. *Soil Microbiology, Ecology and Biochemistry*. USA: Academic Press.

Pax F, Wulfert K. 1941. Die Rädertiere der deutschen Thermen. *Lotos* 88: 246-262.

Peacock L, Bailey M, Carrington M, Gibson W. 2014. Meiosis and Haploid Gametes in the Pathogen *Trypanosoma brucei*. *Current Biology* 24: 181-186.

Peacock L, Ferris V, Sharma R, Sunter J, Bailey M, Carrington M, Gibson W. 2011. Identification of the meiotic life cycle stage of *Trypanosoma brucei* in the tsetse fly. *Proceedings of the National Academy of Sciences of the United States of America* 108: 3671-3676.

Pejler B. 1995. Relation to habitat in rotifers. *Hydrobiologia* 313: 267-278.

Perez M, Valverde J, Batuecas B, Amat F, Marco R, Garesse R. 1994. Speciation in the *Artemia* genus: mitochondrial DNA analysis of bisexual and parthenogenetic brine shrimps. *Journal of Molecular Evolution* 38: 156-168.

Pieri V, Martens K, Stoch F, Rossetti G. 2009. Distribution and ecology of non-marine ostracods (Crustacea, Ostracoda) from Friuli Venezia Giulia (NE Italy). *Journal of Limnology* 68: 1-15.

Pilato G. 1979. Correlations between cryptobiosis and other biological characteristics in some soil animals. *Italian Journal of Zoology* 46: 319-332.

Pinto R, Rocha C, Martens K. 2005. On new terrestrial ostracods (Crustacea, Ostracoda) from Brazil, primarily from Sao Paulo State. *Zoological Journal of the Linnean Society* 145: 145-173.

Poinar GO, Ricci C. 1992. Bdelloid rotifers in Dominican amber: evidence for parthenogenetic continuity. *Experientia* 48: 408-410.

Pokorný V. 1965. *Principles of Zoological Micropalaeontology: Vol. 2 International Series of Monographs on Earth Sciences*. Oxford: Pergamon Press.

Pongratz N, Storhas M, Carranza S, Michiels N. 2003. Phylogeography of competing sexual and parthenogenetic forms of a freshwater flatworm: patterns and explanations. *Bmc Evolutionary Biology* 3.

Ponyi H. 1992. Studies of the crustacean fauna of a Hungarian hot spring (Héviz Spa). *Zoologischen Jahrbüchern abhteilung für Systematik Ökologie und Geographie* 119: 397-404.

Pouchkina-Stantcheva NN, McGee BM, Boschetti C, Tolleter D, Chakrabortee S, Popova AV, Meersman F, Macherel D, Hincha DK, Tunnacliffe A. 2007. Functional divergence of former alleles in an ancient asexual invertebrate. *Science* 318: 268-271.

Provencher L, Morse G, Weeks A, Normark B. 2005. Parthenogenesis in the *Aspidiotus nerii* complex (Hemiptera: Diaspididae): a single origin of a worldwide, polyphagous lineage associated with *Cardinium* bacteria. *Annals of the Entomological Society of America* 98: 629-635.

Quattro J, Avise J, Vrijenhoek R. 1992. An ancient clonal lineage in the fish genus *Poeciliopsis* (Atheriniformes: Poeciliidae). *Proceedings of the National Academy of Sciences of the United States of America* 89: 348-352.

Quesada CA, Miranda AC, Hodnett MG, Santos AJB, Miranda HS, Breyer LM. 2004. Seasonal and depth variation of soil moisture in a burned open savanna (campo sujo) in central Brazil. *Ecological Applications* 14: 33-41.

Radakovits R, Jinkerson R, Fuerstenberg S, Tae H, Settlage R, Boore J, Posewitz M. 2012. Draft genome sequence and genetic transformation of the oleaginous alga *Nannochloropis gaditana*. *Nature Communications* 3.

Ramesh M, Malik S, Logsdon J. 2005. A phylogenomic inventory of meiotic genes: evidence for sex in *Giardia* and an early eukaryotic origin of meiosis. *Current Biology* 15: 185-191.

Ranta E. 1979. Population biology of *Darwinula stevensoni* (Crustacea, Ostracoda) in an oligotrophic lake. *Annales Zoologici Fennici* 16: 28-35.

Reeves J, De Deckker P, Halse S. 2007. Groundwater Ostracods from the arid Pilbara region of northwestern Australia: distribution and water chemistry. *Hydrobiologia* 585: 99-118.

Remy W, Taylor T, Hass H, Kerp H. 1994. Four hundred-million-year-old vesicular arbuscular mycorrhizae. *Proceedings of the National Academy of Sciences of the United States of America* 91: 11841-11843.

Retrum J, Hasiotis S, Kaesler R. 2011. Neoichnological experiments with the freshwater ostracode *Heterocypris incongruens*: implications for reconstructing aquatic settings. *Palaios* 26: 509-518.

Ricci C. 1987. Ecology of bdelloids: how to be successful. *Hydrobiologia* 147: 117-127.

Ricci C. 2001. Dormancy patterns in rotifers. *Hydrobiologia* 446: 1-11.

Ricci C, Balsamo M. 2000. The biology and ecology of lotic rotifers and gastrotrichs. *Freshwater Biology* 44: 15-28.

Ricci C, Melone G, Walsh EJ. 2001. A carnivorous bdelloid rotifer, *Abrochtha carnivora* n. sp. *Invertebrate Biology* 120: 136-141.

Ricci C, Perletti F. 2006. Starve and survive: stress tolerance and life-history traits of a bdelloid rotifer. *Functional Ecology* 20: 340-346.

Robeson MS, King AJ, Freeman KR, Birky CW, Martin AP, Schmidt SK. 2011. Soil rotifer communities are extremely diverse globally but spatially autocorrelated locally. *Proceedings of the National Academy of Sciences of the United States of America* 108: 4406-4410.

Rohde K. 1986. Differences in species diversity of Monogenea between the Pacific and Atlantic Oceans. *Hydrobiologia* 137(1): 21-28.

Rohde K, Heap M. 1998. Latitudinal differences in species and community richness and in community structure of metazoan endo- and ectoparasites of marine teleost fish. *International Journal For Parasitology* 28(3): 461-474.

Rosendahl S, Taylor J. 1997. Development of multiple genetic markers for studies of genetic variation in arbuscular mycorrhizal fungi using AFLP(TM). *Molecular Ecology* 6: 821-829.

Rossetti G, Martens K. 1996. Redescription and morphological variability of *Darwinula stevensoni* (Brady & Robertson, 1870) (Crustacea, Ostracoda). *Bulletin Koninklijk Belgisch Instituut voor Natuurwetenschappen* 66: 73-92.

Rossetti G, Martens K. 1998. Taxonomic revision of the recent and Holocene representatives of the family Darwinulidae (Crustacea, Ostracoda), with a description of three new genera. *Bulletin Koninklijk Belgisch Instituut voor Natuurwetenschappen* 68: 55-110.

Rossetti G, Pinto R, Martens K. 2011. Description of a new genus and two new species of Darwinulidae (Crustacea, Ostracoda), from Christmas Island (Indian Ocean) with some considerations on the morphological evolution of ancient asexuals. *Belgian Journal of Zoology* 141: 55-74.

Rossi V, Schön I, Butlin R, Menozzi P. 1998. Clonal genetic diversity. In: Martens K, ed. *Sex and Parthenogenesis: Evolutionary Ecology of Reproductive Modes in Non-Marine Ostracds*. Leiden: Backhuys, 257-274.

Rossi V, Todeschi E, Gandolfi A, Invidia M, Menozzi P. 2002. Hypoxia and starvation tolerance in individuals from a riverine and a lacustrine population of *Darwinula stevensoni* (Crustacea: Ostracoda). *Archiv Fur Hydrobiologie* 154: 151-171.

Sandoval C, Carmean D, Crespi B. 1998. Molecular phylogenetics of sexual and parthenogenetic *Timema* walking-sticks. *Proceedings of the Royal Society B-Biological Sciences* 265: 589-595.

Sandoval C, Vickery V. 1996. *Timema douglasi* (Phasmatoptera: Timematodea), a new parthenogenetic species from southwestern Oregon and northern California, with notes on other species. *Canadian Entomologist* 128: 79-84.

Schaefer I, Domes K, Heethoff M, Schneider K, Schön I, Norton RA, Scheu S, Maraun M. 2006. No evidence for the 'Meselson effect' in parthenogenetic oribatid mites (Oribatida, Acari). *Journal of Evolutionary Biology* 19: 184-193.

Scholl K, Devetter M. 2013. Soil rotifers new to Hungary from the Gemenc floodplain (Duna-Drava National Park, Hungary). *Turkish Journal of Zoology* 37: 406-412.

Schön I, Arkhipova I. 2006. Two families of non-LTR retrotransposons, *Syrinx* and *Daphne*, from the Darwinulid ostracod, *Darwinula stevensoni*. *Gene* 371: 296-307.

Schön I, Butlin R, Griffiths H, Martens K. 1998. Slow molecular evolution in an ancient asexual ostracod. *Proceedings of the Royal Society of London Series B-Biological Sciences* 265: 235-242.

Schön I, Gandolfi A, Di Masso E, Rossi V, Griffiths H, Martens K, Butlin R. 2000. Persistence of asexuality through mixed reproduction in *Eucypris virens* (Crustacea, Ostracoda). *Heredity* 84: 161-169.

Schön I, Martens K. 2003. No slave to sex. *Proceedings of the Royal Society of London Series B-Biological Sciences* 270: 827-833.

Schön I, Martens K. 2004. Adaptive, pre-adaptive and non-adaptive components of radiations in ancient lakes: a review. *Organisms Diversity & Evolution* 4: 137-156.

Schön I, Martens K, Van Doninck K, Butlin R. 2003. Evolution in the slow lane: molecular rates of evolution in sexual and asexual ostracods (Crustacea: Ostracoda). *Biological Journal of the Linnean Society* 79: 93-100.

Schön I, Rossetti G, Martens K. 2009. Darwinulid ostracods: ancient asexual scandals or scandalous gossip? In: Schön I, Martens K, van Dijk P, eds. *Lost Sex: The Evolutionary Biology of Parthenogenesis*. Dordrecht: Springer Science+Business Media B.V., 217-240.

Schurko A, Neiman M, Logsdon J. 2009. Signs of sex: what we know and how we know it. *Trends in Ecology & Evolution* 24: 208-217.

Schwander T. 2016. Evolution: The end of an ancient asexual scandal. *Current Biology* 26: R233–R235.

Schwander T, Crespi B. 2009a. Multiple direct transitions from sexual reproduction to apomictic parthenogenesis in *Timema* stick insects. *Evolution* 63: 84-103.

Schwander T, Crespi B. 2009b. Twigs on the tree of life? Neutral and selective models for integrating macroevolutionary patterns with microevolutionary processes in the analysis of asexuality. *Molecular Ecology* 18: 28-42.

Schwander T, Crespi B, Gries R, Gries G. 2013. Neutral and selection-driven decay of sexual traits in asexual stick insects. *Proceedings of the Royal Society B: Biological Sciences* 280.

Schwander T, Henry L, Crespi B. 2011. Molecular evidence for ancient asexuality in *Timema* stick insects. *Current Biology* 21: 1129-1134.

Schwander T, Vuilleumier S, Dubman J, Crespi B. 2010. Positive feedback in the transition from sexual reproduction to parthenogenesis. *Proceedings of the Royal Society B-Biological Sciences* 277: 1435-1442.

Scott H. 1961. Classification of Ostracoda. In: Moore R, Pitrat C, eds. *Treatise on Invertebrate Paleontology. Part Q. Arthropoda 3. Crustacea Ostracoda*. Lawrence: Geological Society of America and University of Kansas Press, 74-92.

Segers H. 2007. Annotated checklist of the rotifers (Phylum Rotifera), with notes on nomenclature, taxonomy and distribution. *Zootaxa*: 1-104.

Segers H. 2008. Global diversity of rotifers (Rotifera) in freshwater. *Hydrobiologia* 595: 49-59.

Sheldon P. 1996. Plus ça change—a model for stasis and evolution in different environments. *Palaeogeography, Palaeoclimatology, Palaeoecology* 127: 209–227.

Sibley L, Boothroyd J. 1992. Virulent strains of *Toxoplasma gondii* comprise a single clonal lineage. *Nature* 359: 82-85.

Siepel H. 1994. Life-history tactics of soil microarthropods. *Biology and Fertility of Soils* 18(4): 263-278.

Siepel H. 1996. Biodiversity of soil microarthropods: the filtering of species. *Biodiversity & Conservation* 5: 251-260.

Signorovitch A, Dellaporta S, Buss L. 2005. Molecular signatures for sex in the Placozoa. *Proceedings of the National Academy of Sciences of the United States of America* 102: 15518-15522.

Signorovitch A, Hur J, Gladyshev E, Meselson M. 2015. Allele sharing and evidence for sexuality in a mitochondrial clade of bdelloid rotifers. *Genetics* 200: 581-590.

Signorovitch A, Hur J, Gladyshev E, Meselson M. 2016. Evidence for meiotic sex in bdelloid rotifers. *Current Biology* 26: R754–R755.

Simon J, Leterme N, Latorre A. 1999. Molecular markers linked to breeding system differences in segregating and natural populations of the cereal aphid *Rhopalosiphum padi* L. *Molecular Ecology* 8: 965-973.

Simon J, MartinezTorres D, Latorre A, Moya A, Hebert P. 1996. Molecular characterization of cyclic and obligate parthenogens in the aphid *Rhopalosiphum padi* (L). *Proceedings of the Royal Society B-Biological Sciences* 263: 481-486.

Skubala P, Gulvik M. 2005. Pioneer oribatid mite communities (Acari, Oribatida) in newly exposed natural (glacier foreland) and anthropogenic (post-industrial dump) habitats. *Polish Journal of Ecology* 53: 395-407.

Smith R, Kamiya T, Horne D. 2006. Living males of the 'ancient asexual' Darwinulidae (Ostracoda: Crustacea). *Proceedings of the Royal Society B-Biological Sciences* 273: 1569-1578.

Sohlenius B, Bostrom S. 2005. The geographic distribution of metazoan microfauna on East Antarctic nunataks. *Polar Biology* 28: 439-448.

Sorensen MV, Giribet G. 2006. A modern approach to rotiferan phylogeny: combining morphological and molecular data. *Molecular Phylogenetics and Evolution* 40: 585-608.

Speijer D, Lukes J, Elias M. 2015. Sex is a ubiquitous, ancient, and inherent attribute of eukaryotic life. *Proceedings of the National Academy of Sciences of the United States of America* 112: 8827-8834.

Spolsky CM, Phillips CA, Uzzell T. 1992. Antiquity of clonal salamander lineages revealed by mitochondrial DNA. *Nature* 356: 706-708.

Stanley S. 1979. *Macroevolution, Pattern and Process*. Johns Hopkins University Press.

Stenberg P, Lundmark M. 2004. Distribution, mechanisms and evolutionary significance of clonality and polyploidy in weevils. *Agricultural and Forest Entomology* 6: 259-266.

Straub E. 1952. Mikropaläontologische Untersuchungen im Tertiär zwischen Ehingen und Ulm ad Donau. *Geoligisches Jahrbucher* 6: 433-523.

Stukenbrock E, Rosendahl S. 2005. Clonal diversity and population genetic structure of arbuscular mycorrhizal fungi (*Glomus* spp.) studied by multilocus genotyping of single spores. *Molecular Ecology* 14: 743-752.

Subías L. 2004. Listado sistemático, sinonímico y biogeográfico de los ácaros oribátidos (Acariformes, Oribatida) del mundo (1758-2002). *Graellsia* 60: 3-305.

Suga H, Chen Z, de Mendoza A, Sebe-Pedros A, Brown M, Kramer E, Carr M, Kerner P, Vervoort M, Sanchez-Pons N, Torruella G, Derelle R, Manning G, Lang B, Russ C, Haas B, Roger A, Nusbaum C, Ruiz-Trillo I. 2013. The *Capsaspora* genome reveals a complex unicellular prehistory of animals. *Nature Communications* 4.

Suttle CS. 2005. Viruses in the sea. *Nature* 437: 356-361.

Suttle CS. 2007. Marine viruses—major players in the global ecosystem. *Nature Reviews Microbiology* 5: 801-812.

Suttle CS, Chan AM, Cottrell MT. 1990. Infection of phytoplankton by viruses and reduction of primary productivity. *Nature* 347: 467-469.

Taberly G. 1987. Recherches sur la parthénogenèse thélytoque de deux espèces d'acariens oribates: *Trhypochthonius tectorum* (Berlese) et *Platynothrus peltifer* (Koch). III: Etude anatomique, histologique et cytologique des femelles parthénogénétiques. *Acarologia* 28: 389-403.

Taberly G. 1988. Recherches sur la parthénogenèse thélytoque de deux espèces d'Acariens Oribates: *Trhypochthonius tectorum* (Berlese) et *Platynothrus peltifer* (Koch). IV: Observations sur les mâles ataviques. *Acarologia* 29: 95-107.

Tay W, O'Mahony E, Paxton R. 2005. Complete rRNA gene sequences reveal that the microsporidium *Nosema bombi* infects diverse bumblebee (*Bombus* spp.) hosts and contains multiple polymorphic sites. *Journal of Eukaryotic Microbiology* 52: 505-513.

Taylor D, Ó Foighil D. 2000. Transglobal comparisons of nuclear and mitochondrial genetic structure in a marine polyploid clam (*Lasaea*, Lasaeidae). *Heredity* 84: 321-330.

Tétart J. 1978. Les garnitures chromosomiques des Ostracodes d’eau douce. *Travaux du laboratoire d'hydrobiologie de l'Université de Grenoble* 39: 113-140.

Thiriot-Quivreux C. 1992. Karyotype of *Lasaea australis*, a brooding bivalve species. *Australian Journal of Marine and Freshwater Research* 43: 403-408.

Thiriot-Quivreux C, Soyer J, Debovee F, Albert P. 1988. Unusual chromosome complement in the brooding bivalve *Lasaea consanguinea*. *Genetica* 76: 143-151.

Thorp J, Covich A. 1991. *Ecology and classification of North American freshwater invertebrates, v. 2*. San Diego: Academic Press.

Tibayrenc M. 1997. Are *Candida albicans* natural populations subdivided? *Trends in Microbiology* 5: 253-254.

Tibayrenc M, Kjellberg F, Arnaud J, Oury B, Breniere S, Darde M, Ayala F. 1991. Are eukaryotic microorganisms clonal or sexual? A population genetics vantage. *Proceedings of the National Academy of Sciences of the United States of America* 88: 5129-5133.

Tibayrenc M, Kjellberg F, Ayala F. 1990. A clonal theory of parasitic protozoa: the population structures of *Entamoeba*, *Giardia*, *Leishmania*, *Naegleria*, *Plasmodium*, *Trichomonas*, and *Trypanosoma* and their medical and taxonomical consequences. *Proceedings of the National Academy of Sciences of the United States of America* 87: 2414-2418.

Tigano M, Carneiro R, Jeyaprakash A, Dickson D, Adams B. 2005. Phylogeny of *Meloidogyne* spp. based on 18S rDNA and the intergenic region of mitochondrial DNA sequences. *Nematology* 7: 851-862.

Tinn O, Oakley T. 2008. Erratic rates of molecular evolution and incongruence of fossil and molecular divergence time estimates in Ostracoda (Crustacea). *Molecular Phylogenetics and Evolution* 48: 157-167.

Tisserant E, Malbreil M, Kuo A, Kohler A, Symeonidi A, Balestrini R, Charron P, Duensing N, Frey N, Gianinazzi-Pearson V, Gilbert L, Handa Y, Herr J, Hijri M, Koul R, Kawaguchi M, Krajinski F, Lammers P, Masclauxm F, Murat C, Morin E, Ndikumana S, Pagni M, Petitpierre D, Requena N, Rosikiewicz P, Riley R, Saito K, Clemente H, Shapiro H, Van Tuinen D, Becard G, Bonfante P, Paszkowski U, Shachar-Hill Y, Tuskan G, Young P, Sanders I, Henrissat B, Rensing S, Grigoriev I, Corradi N, Roux C, Martin F. 2013. Genome of an arbuscular mycorrhizal fungus provides insight into the oldest plant symbiosis. *Proceedings of the National Academy of Sciences of the United States of America* 110: 20117-20122.

Tobler M, Schlupp I, de Leon F, Glaubrecht M, Plath M. 2007. Extreme habitats as refuge from parasite infections? Evidence from an extremophile fish. *Acta Oecologica* 31: 270-275.

Tokeshi M. 1999. *Species Coexistence: Ecological and Evolutionary Perspectives*. Oxford, UK: Blackwell Science Ltd.

Triantaphyllou A. 1981. Oogenesis and the chromosomes of the parthenogenetic root-knot nematode *Meloidogyne incognita*. *Journal of Nematology* 13: 95-104.

Trudgill DL, Blok VC. 2001. Apomictic, polyphagous root-knot nematodes: exceptionally successful and damaging biotrophic root pathogens. *Annual Review of Phytopathology* 39: 53-77.

Tudorancea C, Green R, Huebner J. 1979. Structure, dynamics and production of the benthic fauna in Lake Manitoba. *Hydrobiologia* 64: 59-95.

Tyler-Walters H, Davenport J. 1990. The relationship between the distribution of genetically distinct inbred lines and upper lethal temperature in *Lasaea rubra*. *Journal of the Marine Biological Association of the United Kingdom* 70: 557-570.

Tzung K, Williams R, Scherer S, Federspiel N, Jones T, Hansen N, Bivolarevic V, Huizar L, Komp C, Surzycki R, Tamse R, Davis R, Agabian N. 2001. Genomic evidence for a complete sexual cycle in *Candida albicans*. *Proceedings of the National Academy of Sciences of the United States of America* 98: 3249-3253.

Vandenkoornhuyse P, Leyval C, Bonnin I. 2001. High genetic diversity in arbuscular mycorrhizal fungi: evidence for recombination events. *Heredity* 87: 243-253.

Verma K. 1969. A new subspecies of *Impatientinum impatiensae* (Shinji) and the male of *Protrama penecaeca* (Stroyan) from NW India (Homoptera: Aphididae). *Bulletin of entomology* 10: 102-103.

Vickery V. 1993. Revision of *Timema* (Scudder) (Phasmatoptera: Timematodea) including three new species. *Canadian Entomologist* 125: 657-692.

Vickery V, Sandoval C. 2001. Descriptions of three new species of *Timema* (Phasmatoptera: Timematodea: Timematidae) and notes on three other species. *Journal of Orthoptera Research* 10: 53-61.

Villesen P, Mueller U, Schultz T, Adams R, Bouck A. 2004. Evolution of ant-cultivar specialization and cultivar switching in *Apterostigma* fungus-growing ants. *Evolution* 58: 2252-2265.

Vrijenhoek R, Parker E. 2009. Geographical parthenogenesis: general purpose genotypes and frozen niche variation. In: Schön I, Martens K, van Dijk P, eds. *Lost Sex: The Evolutionary Biology of Parthenogenesis*: Dordrecht: Springer Science+Business Media B.V., 99-131.

Waggoner BM, Poinar GO. 1993. Fossil habrotrochid rotifers in Dominican amber. *Experientia* 49: 354-357.

Wallace R, Ricci C, Melone G. 1996. A cladistic analysis of pseudocoelomate (aschelminth) morphology. *Invertebrate Biology* 115: 104-112.

Wallwork, JA. 1970. *Ecology of soil animals*. London, UK: McGRAW-HILL Publishing Company Limited.

Walter D. 2001. Endemism and cryptogenesis in 'segmented' mites: a review of Australian Alicorhagiidae, Terpnacaridae, Oehserchestidae and Grandjeanicidae (Acari: sarcoptiformes). *Australian Journal of Entomology* 40: 207-218.

Walter D. 2009. Suborder Endeostigmata. In: Krantz G, Walter D, eds. *A manual of acarology*. Lubbock: Texas Tech University Press, 421-429.

Walter D, Lindquist E, Smith I, Cook D, Krantz G. 2009. Order Trombidiformes. In: Krantz G, Walter D, eds. *A manual of acarology*. Lubbock: Texas Tech University Press, 233-420.

Weedall G, Hall N. 2015. Sexual reproduction and genetic exchange in parasitic protists. *Parasitology* 142: S120-S127.

Weiss M. 2001. Widespread hermaphroditism in freshwater gastrotrichs. *Invertebrate Biology* 120: 308-341.

Welch D. 2000. Evidence from a protein-coding gene that acanthocephalans are rotifers. *Invertebrate Biology* 119: 17-26.

Welch D, Cummings MP, Hillis DM, Meselson M. 2004a. Divergent gene copies in the asexual class Bdelloidea (Rotifera) separated before the bdelloid radiation or within bdelloid families. *Proceedings of the National Academy of Sciences of the United States of America* 101: 1622-1625.

Welch D, Meselson M. 2000. Evidence for the evolution of bdelloid rotifers without sexual reproduction or genetic exchange. *Science* 288: 1211-1215.

Welch D, Ricci C, Meselson M. 2009. Bdelloid rotifers: progress in understanding the success of an evolutionary scandal. In: Schön I, Martens K, van Dijk P, eds. *Lost Sex: The Evolutionary Biology of Parthenogenesis*. Dordrecht: Springer Science+Business Media B.V., 259-279.

Welch D, Welch J, Meselson M. 2008. Evidence for degenerate tetraploidy in bdelloid rotifers. *Proceedings of the National Academy of Sciences of the United States of America* 105: 5145-5149.

Welch J, Welch D, Meselson M. 2004b. Cytogenetic evidence for asexual evolution of bdelloid rotifers. *Proceedings of the National Academy of Sciences of the United States of America* 101: 1618-1621.

Welch J, Meselson M. 1998. Karyotypes of bdelloid rotifers from three families. *Hydrobiologia* 387: 403-407.

Wickstrom C, Castenholz R. 1985. Dynamics of cyanobacterial and ostracod interactions in an Oregon hot spring. *Ecology* 66: 1024-1041.

Wilson C. 2011. Desiccation-tolerance in bdelloid rotifers facilitates spatiotemporal escape from multiple species of parasitic fungi. *Biological Journal of the Linnean Society* 104(3): 564-574.

Wilson C, Sherman P. 2010. Anciently asexual bdelloid rotifers escape lethal fungal parasites by drying up and blowing away. *Science* 327: 574-576.

Witek A, Herlyn H, Meyer A, Boell L, Bucher G, Hankeln T. 2008. EST based phylogenomics of Syndermata questions monophyly of Eurotatoria. *Bmc Evolutionary Biology* 8.

Wommack KE, Colwell RR. 2000. Virioplankton: Viruses in Aquatic Ecosystems. *Microbiology and Molecular Biology Reviews* 64: 69-114.

Wrensch D, Kethley J, Norton R. 1994. Cytogenetics of holokinetic chromosomes and inverted meiosis: keys to the evolutionary success of mites, with generalizations on eukaryotes. In: Houck M, ed. *Mites. Ecological and Evolutionary Analyses of Life-History Patterns*. New York: Chapman & Hall, 282-343.

Wysocka A, Konopa G, Wegrzyn G, Wrobel B. 2006. Genomic DNA hybridization as an attempt to evaluate phylogenetic relationships of Ostracoda. *Crustaceana* 79: 1309-1322.

Yamada S. 2007. Structure and evolution of podocopan ostracod hinges. *Biological Journal of the Linnean Society* 92: 41-62.

Yamaguchi S, Endo K. 2003. Molecular phylogeny of Ostracoda (Crustacea) inferred from 18S ribosomal DNA sequences: implication for its origin and diversification. *Marine Biology* 143: 23-38.

Yu N, Zhao M, Chen L, Yang P. 2006. Phylogenetic relationship of Podocopida (Ostracoda: Podocopa) based on 18S ribosomal DNA sequences. *Acta Oceanologica Sinica* 25: 99-108.
